# Disruption of GMNC-MCIDAS multiciliogenesis program is critical in choroid plexus carcinoma development

**DOI:** 10.1038/s41418-022-00950-z

**Published:** 2022-03-23

**Authors:** Qun Li, Zhiyuan Han, Navleen Singh, Berta Terré, Ryann M. Fame, Uzayr Arif, Thomas D. Page, Tasneem Zahran, Ahmed Abdeltawab, Yuan Huang, Ping Cao, Jun Wang, Hao Lu, Hart G. W. Lidov, Kameswaran Surendran, Lizhao Wu, James Q. Virga, Ying-Tao Zhao, Ulrich Schüller, Robert J. Wechsler-Reya, Maria K. Lehtinen, Sudipto Roy, Zhongmin Liu, Travis H. Stracker, Haotian Zhao

**Affiliations:** 1 Department of Oncology, Shanghai East Hospital, Tongji University School of Medicine, Shanghai, 200123 PR China; 2 Massachusetts General Hospital, Harvard Medical School, Boston, MA 02114 USA; 3 Department of Biomedical Sciences, New York Institute of Technology College of Osteopathic Medicine, Old Westbury, New York, NY 11568 USA; 4 Institute for Research in Biomedicine (IRB Barcelona), The Barcelona Institute of Science and Technology, C/ Baldiri Reixac 10, Barcelona, 08028 Spain; 5 Department of Pathology, Boston Children’s Hospital, Boston, MA 02115 USA; 6 Tumor Initiation and Maintenance Program, Sanford Burnham Prebys Medical Discovery Institute, 10901 North Torrey Pines Road, La Jolla, CA 92037 USA; 7 Institute of Molecular and Cell Biology, Proteos, 61 Biopolis Drive, Singapore, 138673 Singapore; 8 Pediatrics and Rare Diseases Group, Sanford Research, 2301 E 60th Street North, Sioux Falls, SD 57104 USA; 9 Department of Pathophysiology, College of Basic Medical Sciences, China Medical University, Shenyang, 110122 PR China; 10 Research Institute Children’s Cancer Center, University Medical Center Hamburg-Eppendorf, 20246 Hamburg, Germany; 11 Institute of Neuropathology, University Medical Center Hamburg-Eppendorf, 20246 Hamburg, Germany; 12 Department of Pediatric Hematology and Oncology, University Medical Center Hamburg-Eppendorf, 20246 Hamburg, Germany; 13 Department of Pediatrics, Yong Loo Lin School of Medicine, National University of Singapore, 1E Kent Ridge Road, Singapore, 119288 Singapore; 14 Department of Biological Sciences, National University of Singapore, 14 Science Drive 4, Singapore, 117543 Singapore; 15 Department of Cardio-vascular Surgery, Shanghai East Hospital, Tongji University School of Medicine, Shanghai, 200123 PR China; 16 The Institute of Biomedical Engineering & Nanoscience, Shanghai East Hospital, Tongji University School of Medicine, Shanghai, 200120 PR China; 17 Radiation Oncology Branch, Center for Cancer Research, National Cancer Institute, NIH, 9000 Rockville Pike, Building 10, Bethesda, MD 20892 USA; 18 Present Address: The Francis Crick Institute, 1 Midland Road, London, NW1 1AT UK; 19 Present Address: Explora Biolabs, 11175 Flintkote Avenue, Suite B, San Diego, CA 92121 USA

**Keywords:** Cancer models, Paediatric cancer, Cancer, Gene expression, CNS cancer

## Abstract

Multiciliated cells (MCCs) in the brain reside in the ependyma and the choroid plexus (CP) epithelia. The CP secretes cerebrospinal fluid that circulates within the ventricular system, driven by ependymal cilia movement. Tumors of the CP are rare primary brain neoplasms mostly found in children. CP tumors exist in three forms: CP papilloma (CPP), atypical CPP, and CP carcinoma (CPC). Though CPP and atypical CPP are generally benign and can be resolved by surgery, CPC is a particularly aggressive and little understood cancer with a poor survival rate and a tendency for recurrence and metastasis. In contrast to MCCs in the CP epithelia, CPCs in humans are characterized by solitary cilia, frequent *TP53* mutations, and disturbances to multiciliogenesis program directed by the GMNC-MCIDAS transcriptional network. GMNC and MCIDAS are early transcriptional regulators of MCC fate differentiation in diverse tissues. Consistently, components of the GMNC-MCIDAS transcriptional program are expressed during CP development and required for multiciliation in the CP, while CPC driven by deletion of *Trp53* and *Rb1* in mice exhibits multiciliation defects consequent to deficiencies in the GMNC-MCIDAS program. Previous studies revealed that abnormal NOTCH pathway activation leads to CPP. Here we show that combined defects in NOTCH and Sonic Hedgehog signaling in mice generates tumors that are similar to CPC in humans. NOTCH-driven CP tumors are monociliated, and disruption of the NOTCH complex restores multiciliation and decreases tumor growth. NOTCH suppresses multiciliation in tumor cells by inhibiting the expression of GMNC and MCIDAS, while *Gmnc-Mcidas* overexpression rescues multiciliation defects and suppresses tumor cell proliferation. Taken together, these findings indicate that reactivation of the GMNC-MCIDAS multiciliogenesis program is critical for inhibiting tumorigenesis in the CP, and it may have therapeutic implications for the treatment of CPC.

## Introduction

The choroid plexus (CP) in each brain ventricle consists of stromal vasculatures ensheathed by epithelia [1–3]. The CP is responsible for the synthesis and secretion of cerebrospinal fluid in the central nervous system. Recent studies revealed multiciliated cells (MCCs) in the CP of the mouse [1–3]. Unlike ependymal cells that form multiple motile cilia to drive cerebrospinal fluid flow within the central nervous system after birth, MCCs in the CP epithelia arise during embryogenesis, display increased motility of their multiple cilia until birth, and experience a gradual regression in the motility during postnatal life [3–5]. Tumors of the CP comprise ~20% of brain tumors diagnosed in children under 1 year of age [6, 7]. Research aimed at understanding the origin and molecular characteristics of CP carcinoma (CPC) is essential for developing new therapies to improve clinical outcomes [8–12].

MCCs on the epithelial lining of the brain ventricles, the airway, and reproductive tracts control fluid movement through the beating of multiple motile cilia on their apical surface. Multiciliogenesis is directed by a network of transcription factors that include two members of the Geminin family of coiled-coil containing nuclear proteins: Geminin Coiled-Coil Domain Containing (GMNC) and multi-ciliate differentiation and DNA synthesis associated cell cycle protein (MCIDAS) [13, 14]. GMNC and MCIDAS play sequential roles in the early steps of the MCC differentiation program that is triggered by NOTCH inhibition [15–20]. GMNC and MCIDAS activate the expression of downstream MCC factors, including forkhead box J1 (FOXJ1), v-myb avian myeloblastosis viral oncogene homolog (MYB), and cyclin O (CCNO), as well as TP53 family member *TAp73* [21–28].

GMNC and MCIDAS both transcriptionally regulate multiciliogenesis in ependymal cells through the E2F4/5-DP1 transcription factors [15, 16, 29]. In contrast, Geminin antagonizes the transcriptional functions of GMNC and MCIDAS. And Geminin and GMNC play antagonistic roles in the maintenance of the stem and ependymal cell populations in the adult neurogenic niche, respectively [30, 31]. CP epithelial cells are derived from neuroepithelial progenitors that express orthodenticle homeobox 2 (OTX2) and Growth differentiation factor 7 (GDF7). As these progenitors exit the cell cycle to undergo multiciliogenesis and differentiation, TAp73 is activated in MCCs, while aquaporin 1 (AQP1), transthyretin (TTR), and cytokeratins are upregulated in epithelial cells [32]. Our previous work showed that, in contrast to all other MCCs, *TAp73* was dispensable for multiciliogenesis in the CP, suggesting that its differentiation program may be distinct from other MCC types [26]. Therefore, further analysis of the molecular mechanisms governing multiciliogenesis in the CP, as well as the functional significance of these cilia, will be important to understand their role in the pathology of both ciliopathies and CP tumor development.

Examination of human CP tumors revealed abnormal NOTCH activity in a subset of tumors [33], and we demonstrated that sustained NOTCH1 expression in mice led to CP papilloma (CPP) that arose from monociliated progenitors in hindbrain roof plate [34, 35]. These progenitors proliferated in response to Sonic Hedgehog (SHH), but subsequently became quiescent after birth [34–36]. Here, we show that human CPC, and to a lesser extent CPP, display consistent defects in the GMNC-MCIDAS transcriptional program and amplifications of NOTCH pathway components. Using two distinct murine models, we found that CPCs in mice exhibit multiciliation defects and a deficient GMNC program. In addition, persistent NOTCH and SHH signals are sufficient to drive aggressive tumors in mice that resemble human CPC. These tumors display singular primary cilia resulting from the repression of the GMNC-MCIDAS multiciliation program by NOTCH. Biochemical or pharmacological disruption of the NOTCH complex restored multiciliation and suppressed tumor cell proliferation. Our findings indicate that the GMNC-MCIDAS transcriptional network is essential for MCC differentiation in the CP, and its activation can induce multiciliation and decrease CP tumor cell proliferation. These findings underscore the critical role of a compromised GMNC-MCIDAS multiciliogenesis program in CPC development and suggest that this could be exploited therapeutically to impair proliferation and promote tumor differentiation.

## Results

### CPCs in humans exhibit reduced multiciliation and a deficient GMNC-MCIDAS program

**Fig. 1:**
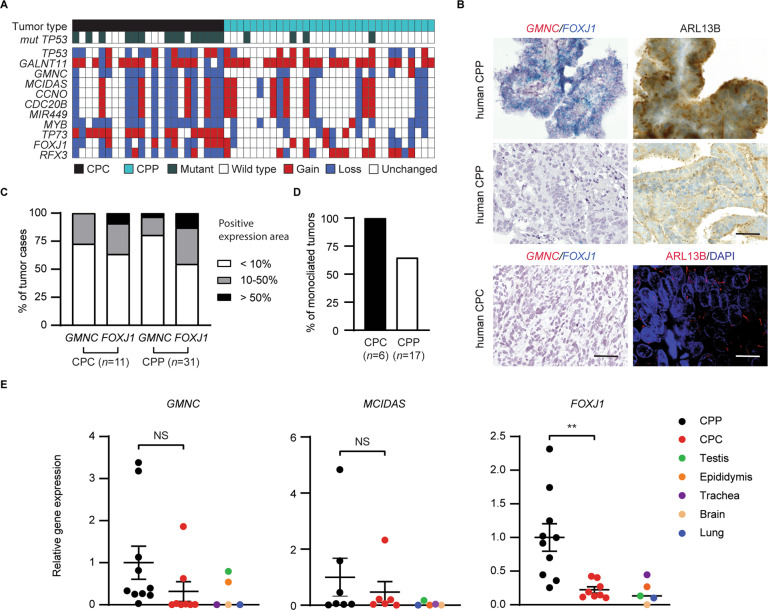
Multiciliation defects and GMNC program deficiencies in CP tumors. **A** Copy number analysis of human CPC (*n* = 23 tumors from 23 individuals) and CPP (*n* = 32 tumors from 32 individuals). **B** Representative images of human CP tumors that show *GMNC* and *FOXJ1* expression by RNAscope (left panels), and ARL13B expression by immunostaining (brown signals in upper right panels, red signals in lower right panel). DAPI staining (blue, lower right panel) labels nuclei. Scale bars, 25 µm. Results were obtained from at least three independent experiments. **C** Summary of *GMNC* and *FOXJ1* expression in human CP tumors shown in **B** (CPC: *n* = 11; CPP: *n* = 31). **D** Summary of cilia status in human CP tumors (CPC: *n* = 6; CPP: *n* = 11). **E** RT-qPCR analysis of the expression of *GMNC*, *MCIDAS* and *FOXJ1* in CP tumors and normal control tissues in humans (CPP: *n* = 10; CPC: *n* = 8; normal tissue: *n* = 1 for brain, trachea, lung, testis, and epididymis, respectively; mean ± s.e.m., student *t*-test, **P < 0.01). Data represent two independent experiments.

Most CP tumors in humans, especially CPCs, consist of monociliated tumor cells and frequently display large-scale genomic alterations [34, 37–39]. Analysis of published data revealed recurrent chromosomal changes that affect loci encompassing multiciliogenesis regulators, including *GMNC* on chromosome 3, that is lost in all hypodiploid CPCs, *MCIDAS*, *CCNO*, microRNA 449 (*MIR449*), and *CDC20B*, that are all located within the same locus of chromosome 5, and *MYB* on chromosome 6, that is lost in many CPCs (Fig. 1A). Conversely, *N*-acetyl galactosamine-type *O*-glycosylation enzyme *GALNT11*, a positive regulator of NOTCH signaling on chromosome 7, is gained in >80% CP tumors (Fig. 1A) [37–40]. In agreement, among 11 cases of human CPCs examined, most displayed significantly reduced or complete loss of *GMNC* expression, and *GMNC* expression was heterogeneous and only detected in a subpopulation of tumor cells (Fig. 1B, C). Decreased *FOXJ1* expression was also observed in the majority of samples (Fig. 1B, C). A similar trend of reduced *GMNC* and *FOXJ1* expression was observed in 31 human CPPs (Fig. 1B, C). Consistent with the requirement for *GMNC* and *FOXJ1* in MCC differentiation, analysis of cilia marker ADP-ribosylation factor-like 13b (ARL13B) in six CPCs found that all were monociliated, while 11 of 17 CPPs analyzed were monociliated (Fig. 1B, D). Accordingly, RT-qPCR analysis revealed low levels of *GMNC* and *MCIDAS* expression in most human CP tumors compared to normal tissues, while *FOXJ1* expression in CPCs was significantly lower than CPPs (Fig. 1E). In contrast, TAp73 expression in human CPC and CPP varied from significantly reduced to normal levels in similar proportions (Supplementary Fig. S1A). Moreover, analysis of a published dataset revealed differential expression of genes involved in ciliogenesis in human CP tumors, contributing to significant enrichment of the pathway (Supplementary Fig. S1B, C) [37]. Thus, CPCs in humans are characterized by multiciliation defects and deficiencies in the GMNC-MCIDAS program, as well as recurrent amplification of NOTCH regulators.

### Gmnc-Mcidas signaling is essential for generating multiciliated epithelia in the CP

**Fig. 2:**
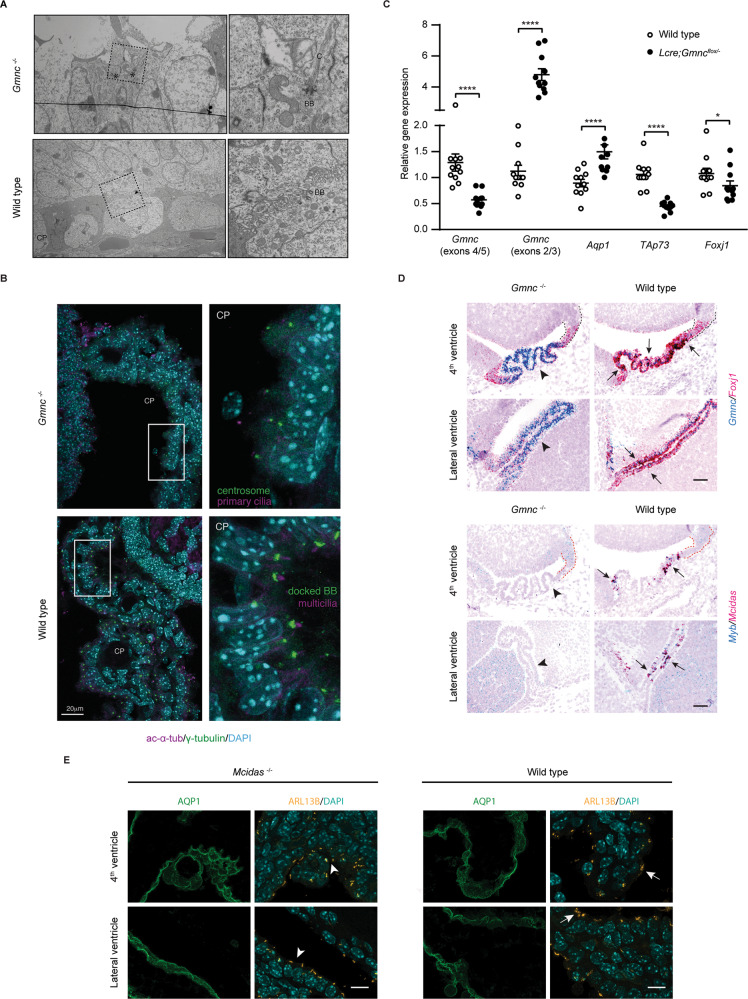
GMNC-MCIDAS transcriptional program is required for MCC formation in the CP. **A** Transmission electron micrographs are shown of CP epithelial cells in newborn *Gmnc*^−^^*/*−^ (asterisks) and wild type (arrow) mice. Boxed regions are magnified on the right. Notice that *Gmnc*^−^^*/*−^ CP epithelial cell exhibits single basal body and solitary cilia compared to wild type epithelial cell with multiple basal bodies. CP choroid plexus, C cilia, BB basal body. Images are representative of at least three independent experiments. **B** The expression of acetylated α-tubulin (ac-α-tub, magenta) and γ-tubulin (green) is shown in the CP epithelial cells in newborn *Gmnc*^−^^*/*−^ and wild type animals. Boxed regions are shown in higher magnification on the right. DAPI staining (cyan) labels nuclei. Scale bar, 20 µm. BB basal body. Images represent three independent experiments. **C** RT-qPCR analysis of the expression of *Gmnc* (primers/probe from exons 4/5 or 2/3), *Aqp1 TAp73*, and *Foxj1* in the CP from *Lcre;Gmnc*^*flox/−*^ and wild type mice at day P7 (*n* = 11 animals per genotype, mean ± s.e.m., paired *t*-test, ****P < 0.0001). Data represent three independent experiments. **D** Representative images of the expression of *Gmnc* and *Foxj1* (upper panel), *Mcidas* and *Myb* (lower panel) are shown at day E13.5 in roof plate (upper roof plate marked by dotted lines) and CP in the hindbrain and the lateral ventricle in *Gmnc*^−^^*/*−^ (arrowheads) and wild type (arrows) animals. Scale bars, 50 µm. Images represent at least three independent experiments. **E** The expression of ARL13B (yellow) and AQP1 (green) is shown in the CP epithelial cells in the hindbrain and lateral ventricles at day P7 in *Mcidas*^−^^*/*−^ (arrowheads^)^ and wild type (arrows) animals. DAPI staining (cyan) labels nuclei. Scale bars, 10 µm. Results were obtained from three independent experiments.

These results suggested that suppressing the MCC fate program controlled by GMNC and MCIDAS was a key step in the genesis of CP tumors. While GMNC was implicated in the formation of MCCs in the CP [15], a detailed examination of its role in the CP has not been carried out. The *Gmnc* conditional allele (*Gmnc*^*flox/flox*^) has two loxP sites located in introns 3 and 5 that allow Cre-mediated deletion of exons 4/5 to generate the null allele (*Gmnc*^−^^*/*−^) [15]. Using electron microscopy and immunostaining, we compared wild type CP to animals with a conditional deletion of *Gmnc* in the roof plate/CP by the *Lmx1a-Cre* transgene [41]. We found that both ependymal cells and the CP epithelium of *Gmnc*^−^^/−^ animals were comprised solely of monociliated cells, compared to wild type controls that exhibited multiple basal bodies and multiciliation (Fig. 2A, B; Supplementary Figs. S2A, B, S3A). RT-qPCR using primers from exons 4/5 revealed significantly reduced *Gmnc* levels in the CP from *Lmx1a-Cre;Gmnc*^*flox/−*^ (*Lcre;Gmnc*^*flox/−*^) mice at postnatal (P) day 7 (P7), consistent with efficient *Gmnc* disruption (Fig. 2C). Analysis of Ki-67 expression showed that both *Gmnc*^−^^/−^ and wild type CP epithelial cells became postmitotic and the expression of epithelial markers cytokeratins, TTR, and OTX2 was comparable between *Gmnc*^−^^/−^ and wild type CP, though *Aqp1* expression was significantly increased in *Gmnc*^−^^/−^ animals (Fig. 2C; Supplementary Fig. S3B–F).

*Gmnc* mRNA was detected in wild type CP epithelial cells adjoining the roof plate at embryonic (E) day 13.5 (E13.5), and *Gmnc* transcripts persisted in the epithelial cells of *Gmnc*^−^^/−^ CP (Fig. 2D). RT-qPCR with primers from exons 2/3 showed increased *Gmnc* levels, and further sequencing revealed a mutant transcript with exon 3 spliced to exon 6 (designated as *Gemc1*^*Δ4-5*^) (Fig. 2C; Supplementary Fig. S4A). The altered splicing causes a frame shift and stop codon after a few amino acids, generating a truncated *Gmnc* transcript lacking crucial functional domains. The expression of GMNC targets *Foxj1* and *TAp73* in the CP and ependyma was markedly reduced in *Gmnc*^−^^/−^ mice, whereas *Gmnc* overexpression stimulated *TAp73* expression (Fig. 2C, D; Supplementary Figs. S4B, D, S5A, C, S14, Supplementary Table 1). Together, these data establish that *Gmnc* is essential for MCC differentiation and the expression of *TAp73* and *Foxj1* in the CP epithelium.

We next examined critical components of the MCC transcriptional cascade, including *Mcidas*, *Myb*, and *Ccno*. All 3 genes were transiently upregulated in a subpopulation of epithelial cells next to the roof plate during development and their expression was lost in *Gmnc*^−^^/−^ mice (Fig. 2D; Supplementary Fig. S5B), indicating that GMNC activates the MCIDAS-dependent program in the CP, as has been shown in other MCC-containing tissues. Examination of CP epithelial cells in *Mcidas*^−^^/−^ mice revealed only solitary primary cilia, and OTX2 and AQP1 expression were similar to that of the wild type CP (Fig. 2E; Supplementary Fig. S6A, B). The expression of *Gmnc*, *Foxj1*, and *TAp73* remained unaltered by *Mcidas* loss, although *Mcidas* overexpression stimulated *TAp73* and *Ccno* expression (Supplementary Figs. S4C, D, S6C, D, S14, Supplementary Table 1). Taken together, these results indicate that MCIDAS plays a critical role in multiciliogenesis downstream of GMNC in the CP.

### Gmnc loss mediates cilia defects in CPCs with deficient Rb1/Trp53 signaling

**Fig. 3:**
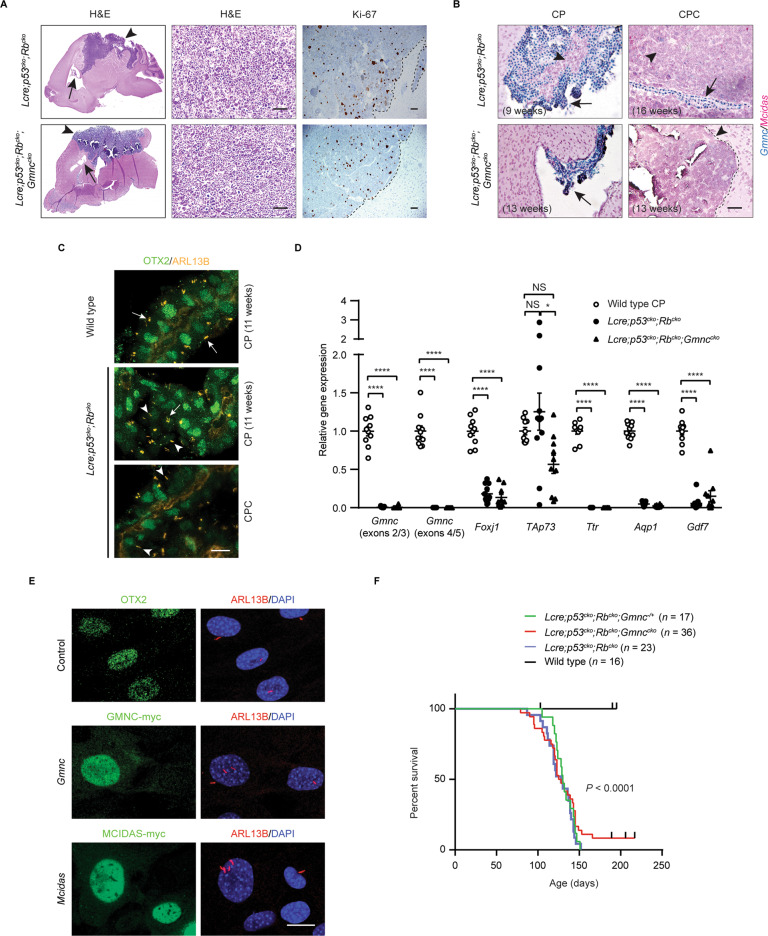
Disruption of GMNC-MCIDAS program mediates multiciliation defects in *Rb1/Trp53*-deficient CPC. **A** Hematoxylin and eosin (H&E) staining and Ki-67 expression are shown in CPC (arrowheads, boundary between tumor and unaffected brain region is marked by dotted lines) and the CP (arrows) from *Lcre;p53*^*cko*^*;Rb*^*cko*^ and *Lcre;p53*^*cko*^*;Rb*^*cko*^;*Gmnc*^*cko*^ animals. Scale bars, 50 µm. Images are representative of at least three independent experiments. **B** RNAscope analysis of *Gmnc* and *Mcidas* expression in tumor cells (arrowheads, boundary between tumor and unaffected brain region is marked by dotted line) and the CP (arrows) in *Lcre;p53*^*cko*^*;Rb*^*cko*^ and *Lcre;p53*^*cko*^*;Rb*^*cko*^;*Gmnc*^*cko*^ animals. Scale bar, 50 µm. Results were obtained from three independent experiments. **C** Representative images of immunofluorescence of ARL13B (yellow) and OTX2 (green) are shown in multiciliated epithelial cells (arrows) or monociliated tumor cells (arrowheads) in wild type and *Lcre;p53*^*cko*^*;Rb*^*cko*^ animals, respectively. Scale bar, 10 µm. Data represent three independent experiments. **D** RT-qPCR analysis of gene expression in wild type CP and CPC from *Lcre;p53*^*cko*^*;Rb*^*cko*^ and *Lcre;p53*^*cko*^*;Rb*^*cko*^;*Gmnc*^*cko*^ animals (wild type CP: *n* = 10; CPC: *n* = 11 for *Lcre;p53*^*cko*^*;Rb*^*cko*^ animals, *n* = 10 for *Lcre;p53*^*cko*^*;Rb*^*cko*^;*Gmnc*^*cko*^ animals; mean ± s.e.m., one-way ANOVA, *P < 0.05, ****P < 0.0001, NS not significant). Data represent three independent experiments. **E** Representative images of immunofluorescence of ARL13B (red) are shown in tumor cells from *Lcre;p53*^*cko*^*;Rb*^*cko*^;*Gmnc*^*cko*^ animals infected with viruses expressing GMNC-myc (green) or MCIDAS-myc (green). OTX2 (green) labels tumor cells. DAPI staining (blue) labels nuclei. Scale bar, 20 µm. Three independent experiments were conducted. **F** Kaplan–Meier curve depicting the survival of *Lcre;p53*^*cko*^*;Rb*^*cko*^, *Lcre;p53*^*cko*^*;Rb*^*cko*^;*Gmnc*^*−/+*^, and *Lcre;p53*^*cko*^*;Rb*^*cko*^;*Gmnc*^*cko*^ animals compared to wild type mice.

CPC frequently occurs in Li-Fraumeni syndrome patients and somatic *TP53* mutations in sporadic CPC predict poor outcome [37, 42]. In mice, *Trp53* deletion combined with *Rb1* loss or *Myc* overexpression leads to CPC with characteristics of their human counterparts [8–10, 42–44]. We crossed *Lmx1a-Cre* mice that express *Cre* in the roof plate/CP [41], with a mouse strain carrying conditional alleles of *Trp53* and *Rb1* (*p53*^*flox/flox*^*;Rb*^*flox/flox*^) to cause their deletion in CPC progenitors [45]. All *Lmx1a-Cre;p53*^*flox/flox*^*;Rb*^*flox/flox*^ (*Lcre;p53*^*cko*^*;Rb*^*cko*^) mice developed CPC, characterized by higher numbers of Ki-67^+^ proliferative cells (Fig. 3A). As early as 9 weeks after birth, a small population of *Gmnc*-negative cells was detected in the CP of *Lcre;p53*^*cko*^*;Rb*^*cko*^ animals, whereas OTX2^+^ monociliated cells were present among MCCs in the CP at 11 weeks of age (Fig. 3B, C). Tumor cells in these mice were monociliated and exhibited significantly reduced *Gmnc* and *Foxj1* levels (Fig. 3B–D; Supplementary Fig. S7A; Supplementary Table 1). Thus, *Rb1/Trp53*-deficient murine CPC recapitulates the multiciliation defects and GMNC program deficiencies in CPC in humans.

The loss of *Gmnc* expression at early stages of tumorigenesis suggested that this may promote the reduced multiciliation observed in *Rb1*/*Trp53*-deficient CPC. To address this, we interbred *Lcre;p53*^*cko*^*;Rb*^*cko*^ mice with *Gmnc*^*flox/−*^ animals. The resulting *Lmx1a-Cre;p53*^*cko*^*;Rb*^*cko*^;*Gmnc*^*flox/−*^ (*Lcre;p53*^*cko*^*;Rb*^*cko*^*;Gmnc*^*cko*^) mice succumbed to CPCs that expressed OTX2 and showed proliferation levels similar to that of *Lcre;p53*^*cko*^*;Rb*^*cko*^ mice (Fig. 3A; Supplementary Fig. S7B). The expression of *Ttr*, *Aqp1*, *Gdf7*, and *Foxj1* was significantly reduced, whereas TAp73 expression was more variable in CPC in these mice (Fig. 3D; Supplementary Fig. S7B; Supplementary Table 1). A non-functional *Gemc1*^*Δ4-5*^ mutant transcript was detectable in the CP of the *Lcre;p53*^*cko*^*;Rb*^*cko*^*;Gmnc*^*cko*^ mice, but its presence was dramatically reduced in tumor cells in these animals, indicating similar impairment in the upstream regulation of *Gmnc* (Fig. 3B, D).

To determine if reactivation of the GMNC-MCIDAS program would affect tumorigenesis, we expressed Myc-tagged GMNC or MCIDAS in tumor cells from *Lcre;p53*^*cko*^*;Rb*^*cko*^*;Gmnc*^*cko*^ mice. Reintroduction of either *Gmnc* or *Mcidas* activated the expression of *TAp73* and *Foxj1* and caused multiciliation of tumor cells (Fig. 3E; Supplementary Fig. S7C, D). In contrast, *Gmnc* loss failed to accelerate tumor development in *Lcre;p53*^*cko*^*;Rb*^*cko*^*;Gmnc*^*cko*^ animals, indicating that GMNC does not play a clear tumor suppressive role (Fig. 3F). Together, these data indicate that a defective GMNC-MCIDAS program mediates cilia defect in *Rb1/Trp53*-deficient CPC and facilitates tumor growth.

### SHH and NOTCH pathways drive CPC

Previous analyses revealed abnormal SHH and NOTCH pathway activities in human CP tumors, including CPC [34]. We previously used a *Rosa26-NICD1* mouse strain that exhibits Cre-mediated expression of the intracellular domain of NOTCH1 (NICD1) and green fluorescent protein (GFP) [46]. After crossing with *Lmx1a-Cre* animals, *Lmx1a-Cre;Rosa26-NICD1* (*Lcre;NICD1*) mice developed CPP that underwent transient SHH-driven proliferation [34].

**Fig. 4:**
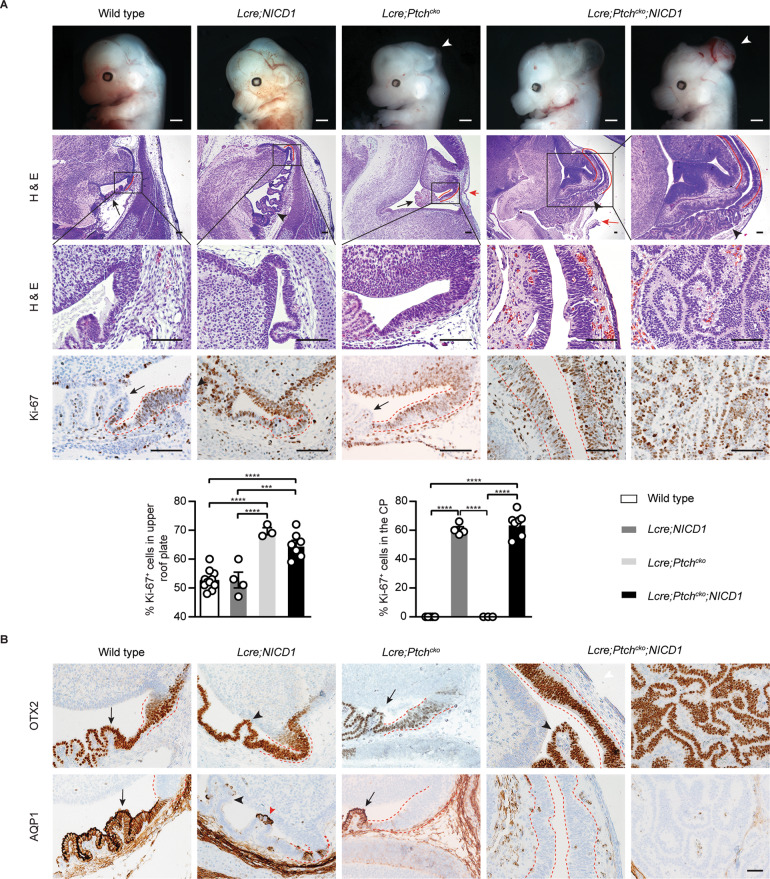
Aberrant NOTCH and SHH signaling drive CPC in mice. **A** Wild type, *Lcre;NICD1*, *Lcre;Ptch*^*cko*^, and *Lcre;Ptch*^*cko*^*;NICD1* animals are shown at day E14.5. Notice the cranium defects resulting from enlarged and folded roof plate in the midbrain-hindbrain region of *Lcre;Ptch*^*cko*^ and *Lcre;Ptch*^*cko*^*;NICD1* animals (white arrowheads). H&E staining and Ki-67 expression are shown of roof plate (upper roof plate marked by red lines) and the CP (black arrows) in the hindbrain in wild type and *Lcre;Ptch*^*cko*^ animals, and CPP and abnormal CP growth (black arrowheads) in *Lcre;NICD1* and *Lcre;Ptch*^*cko*^*;NICD1* animals, respectively. Enlarged roof plate disrupts the cranium in *Lcre;Ptch*^*cko*^ and *Lcre;Ptch*^*cko*^*;NICD1* animals (red arrows). The upper roof plate is shown in higher magnification in the right (*Lcre;Ptch*^*cko*^*;NICD1* animal) and lower (wild type, *Lcre;NICD1*, *Lcre;Ptch*^*cko*^, and *Lcre;Ptch*^*cko*^*;NICD1* animals) panels. Scale bars, 100 µm. Quantification of Ki-67 expression in the upper roof plate and CP in the hindbrain is shown (wild type mice: *n* = 11; *Lcre;NICD1* mice: *n* = 4; *Lcre;Ptch*^*cko*^ mice: *n* = 3; *Lcre;Ptch*^*cko*^*;NICD1* mice: *n* = 7 for upper roof plate, *n* = 8 for the CP; mean ± s.e.m., one-way ANOVA, ***P < 0.001; ****P < 0.0001). Data are representative of at least three independent experiments. **B** Representative results of immunohistochemical staining for OTX2, and AQP1 are shown in the upper roof plate (marked by dotted lines) and the CP (arrows) in the hindbrain at day E14.5 in wild type and *Lcre;Ptch*^*cko*^ animals, and CPP and abnormal CP growth (black arrowheads) in *Lcre;NICD1* and *Lcre;Ptch*^*cko*^*;NICD1* animals, respectively. Residual AQP1-expressing epithelial cells (red arrowhead) are mixed with tumor cells in *Lcre;NICD1* animals. Scale bar, 50 µm. Images represent at least three independent experiments.

To determine whether combined defects in SHH and NOTCH signaling were sufficient to drive more aggressive CPC-like tumors, we bred a mouse strain carrying a *Patched1* conditional allele (*Ptch*^*flox/flox*^) to *Lcre;NICD1* mice. Loss of *Patched1* constitutively activates SHH signaling in the roof plate/CP in *Lmx1a-Cre;Ptch*^*flox/flox*^ (*Lcre;Ptch*^*cko*^) animals [34, 47]. As expected, a thickened upper roof plate was observed in *Lcre;Ptch*^*cko*^ mice at day E14.5 (Fig. 4A). Though most *Lcre;NICD1* mice survived normally, all *Lmx1a-Cre;Ptch*^*flox/flox*^*;NICD1* (*Lcre;Ptch*^*cko*^*;NICD1*) animals died at birth from abnormal cellular growth in the hindbrain region (Fig. 4A). These animals displayed an enlarged upper roof plate with an increased presence of Ki-67^+^ proliferative cells, an abnormal CP growth with increased Ki-67 index, and a loss of monolayer epithelial architecture (Fig. 4A; Supplementary Fig. S8A).

Like CPP in *Lcre;NICD1* mice, the abnormal CP growth in *Lcre;Ptch*^*cko*^*;NICD1* animals exhibited elevated expression of the SHH pathway targets *Gli1* and *Mycn*, reduced *Shh* expression, as well as increased levels of NOTCH targets *Hes1* and *Hes5* (Supplementary Figs. S8B, S9A–C). Similar to *Rb1/Trp53*-deficient CPC, the abnormal CP growth in *Lcre;Ptch*^*cko*^*;NICD1* animals expressed OTX2, and showed reduced expression of AQP1, TTR, and cytokeratins, although some CP epithelial cells were mixed due to incomplete Cre-mediated activation of *NICD1* (Fig. 4B; Supplementary Fig. S8C; Supplementary Table 1). Thus, combined activation of the NOTCH and SHH pathways leads to increased proliferation and pathological cell overgrowth in the upper roof plate region, accompanied by a loss of differentiated epithelial cells in the CP. Moreover, these animals develop malignant CP tumors that closely match the characteristics of CPC in humans.

### NOTCH activation leads to reduced multiciliation in CP tumors

**Fig. 5:**
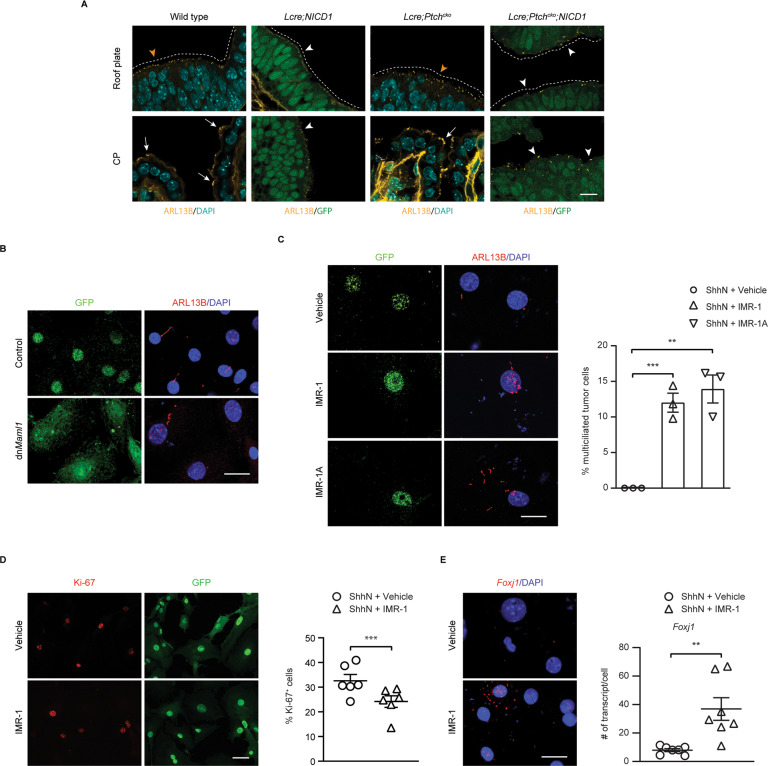
NOTCH activation leads to reduced multiciliation in CP tumors. **A** Immunofluorescent staining for ARL13B (yellow) is shown at day E14.5 in the upper roof plate progenitors (marked by dotted lines and orange arrowheads) and the CP epithelial cells (arrows) in the hindbrain in wild type and *Lcre;Ptch*^*cko*^ animals, and CPP and abnormal CP growth (white arrowheads and dotted lines) in *Lcre;NICD1* and *Lcre;Ptch*^*cko*^*;NICD1* animals, respectively. GFP (green) labels tumor cells. DAPI staining (cyan) labels nuclei. Scale bar, 10 µm. Results were obtained from at least three independent experiments. The expression of ARL13B (red) is shown in tumor cells infected with viruses expressing GFP-tagged dnMAML1 or GFP (**B**), or treated with vehicle, or IMR-1/IMR-1A (**C**). GFP (green) labels infected or treated cells. DAPI staining (blue) labels nuclei. Scale bars, 20 µm. The percentage of multiciliated tumor cells after treatment is shown (*n* = 3, mean ± s.e.m., two-tailed unpaired *t*-test, **P < 0.01, ***P < 0.001). Results were obtained from at least three independent experiments, respectively. **D** The expression of Ki*-*67 (red) is shown in GFP^+^ tumor cells treated with vehicle or IMR-1. Quantitation of Ki-67 expression is shown (*n* = 6 per treatment; mean ± s.e.m., paired *t*-test, ***P < 0.001). Results were obtained from at least three independent experiments. **E** RNAscope analysis of *Foxj1* expression (red) is shown in tumor cells treated with vehicle or IMR-1. DAPI staining (blue) labels nuclei. Scale bar, 20 µm. Quantification of *Foxj1* transcript is shown on the right (*n* = 7 per treatment; mean ± s.e.m., two-tailed unpaired *t*-test, **P < 0.01). Data are representative of three independent experiments.

In contrast to MCCs in the CP, NOTCH-driven CP tumors consisted of monociliated cells with decreased *Foxj1* expression (Fig. 5A) [34], suggesting that NOTCH might mediate reduced multiciliation in CP tumors. To address this, tumor cells from *Lcre;NICD1* mice were treated with a recombinant amino-terminal fragment of SHH (ShhN) and a small molecule Inhibitor of Mastermind Recruitment 1 (IMR-1, or IMR-1A) to block the recruitment of Mastermind-like protein 1 (MAML1) to the NOTCH transcriptional complex, or infected with viruses expressing GFP fused to dominant negative MAML1 (dnMAML1) that disrupts the complex [48, 49]. Remarkably, staining with the cilia markers ARL13B and γ-tubulin revealed GFP^+^ multiciliated tumor cells within 72 h of treatment (Fig. 5B, C; Supplementary Fig. S10A). Consistently, tumor cell proliferation was markedly reduced, and the expression of *Foxj1* was significantly increased by IMR-1 (Fig. 5D, E; Supplementary Fig. S10B).

**Fig. 6:**
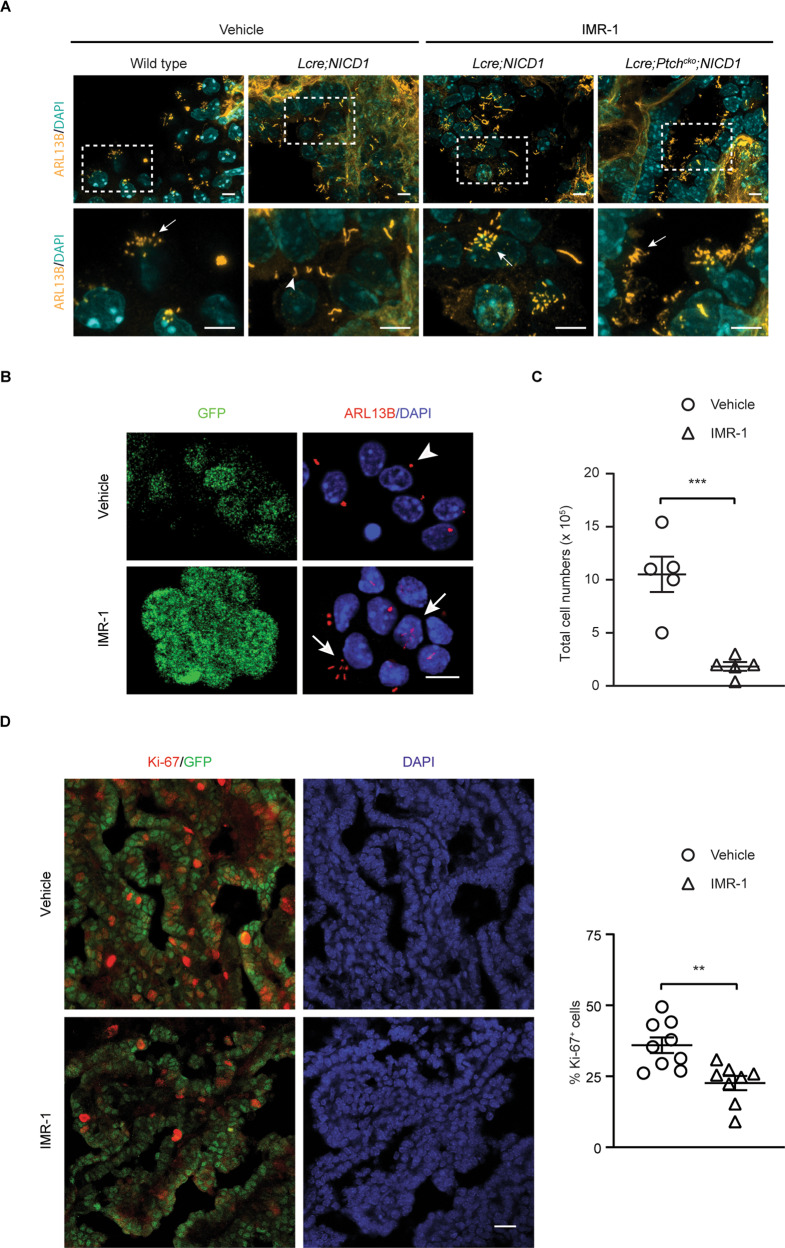
NOTCH inhibition restores multiciliation in CP tumors. Representative images of immunofluorescent staining for ARL13B (**A**, yellow; **B**, red) are shown in tumor cells at day E17.5 (**A**) and tumor cells isolated at day P7 (**B**) from *Lcre;NICD1* (**A**, **B**) and *Lcre;Ptch*^*cko*^*;NICD1* (**A**) animals treated with vehicle or IMR-1 from day E10.5 to day E16.5. Boxed region of ciliated cells is magnified in lower panel (**A**). DAPI staining (**A**, cyan; **B**, blue) labels nuclei. Scale bars, 5 µm (**A**), 10 µm (**B**). Results were obtained from at least three independent experiments. **C** Quantification total tumor cell numbers isolated at day P7 is shown in *Lcre;NICD1* animals treated as described in **A** and **B** (*n* = 5 animals per treatment; mean ± s.e.m., two-tailed unpaired *t*-test, ***P < 0.001). **D** The expression of Ki-67 (red) in NICD1^+^/GFP^+^ tumor cells at day E17.5 is shown in *Lcre;NICD1* animals treated with vehicle or IMR-1 from day E10.5 to day E16.5. DAPI staining (blue) labels nuclei. Scale bar, 20 µm. Quantification of Ki-67 expression in tumor cells is shown (right panel: *n* = 9 animals for vehicle, *n* = 8 animals for IMR-1; mean ± s.e.m., two-tailed unpaired *t*-test, **P < 0.01). Data are representative of three independent experiments.

After a 7-day in vivo IMR-1 treatment from day E10.5, multiciliated tumor cells were detected in *Lcre;NICD1* and *Lcre;Ptch*^*cko*^*;NICD1* animals at day E17.5 and day P7 (Fig. 6A, B). This was accompanied by a significant decrease in tumor cell proliferation, and a reduction of total tumor cell numbers by several folds at day P7 (Fig. 6C, D). Moreover, the expression of SOX2 in the ventricular zone, PAX6, and *Atoh1* in progenitors derived from rhombic lips was comparable between wild type animals treated with IMR-1 or vehicle (Supplementary Fig. S10C, D). Together, these results demonstrate that aberrant NOTCH signaling impairs MCC differentiation in the CP that can be rescued by NOTCH inhibition, leading to reduced tumor growth.

### Gmnc suppression by NOTCH mediates defective multiciliation in CP tumors

**Fig. 7:**
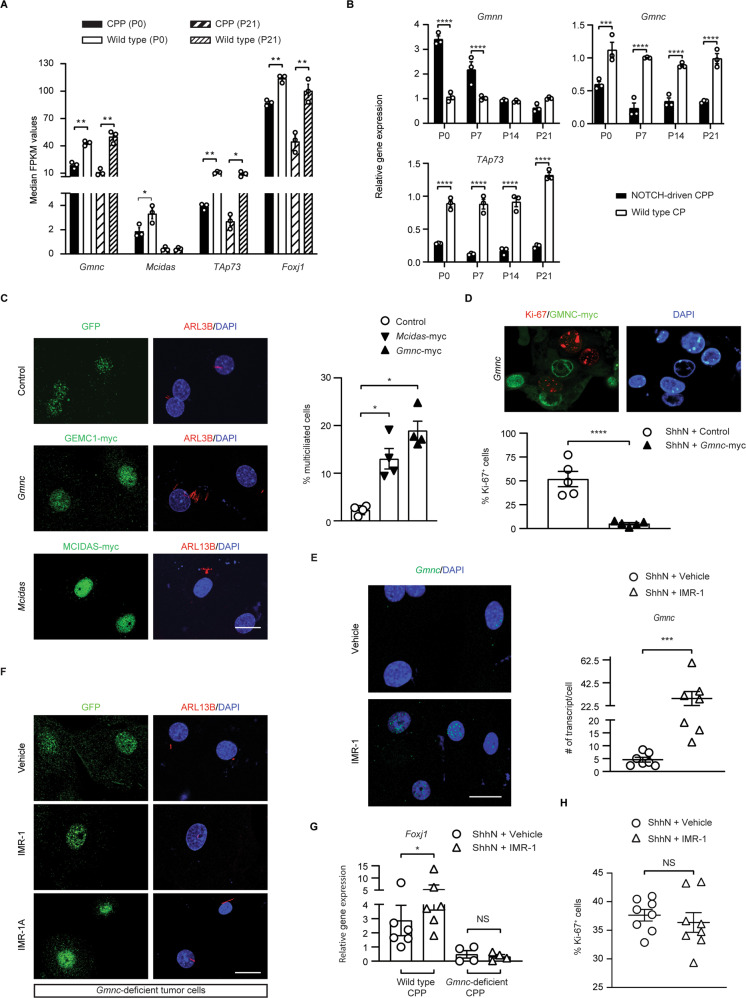
*Gmnc* suppression by NOTCH mediates multiciliation defects in CP development and tumorigenesis. **A** Median FKPM (fragments per kilobase of exon per million reads mapped) values of genes in NOTCH-driven CP tumors and wild type CPs (*n* = 3 specimens per time point, mean ± s.e.m., two-tailed unpaired *t*-test, *P < 0.05; **P < 0.01). **B** RT-qPCR analysis of NOTCH-driven CPP and wild type CP (*n* = 3 animals per time point, mean ± s.e.m., two-tailed unpaired *t*-test, ***P < 0.001, ****P < 0.0001). Three independent experiments were conducted. **C** The expression of ARL13B (red) is shown in tumor cells infected with viruses expressing GMNC-myc, MCIDAS-myc, or control only. GMNC-myc (green), or MCIDAS-myc (green) labels infected cells. DAPI staining (blue) labels nuclei. Scale bar, 20 µm. Quantification of the percentage of MCCs in infected cells is shown on the right (*n* = 4 per treatment, mean ± s.e.m., one-way ANOVA, *P < 0.05). Results were obtained from three independent experiments. **D** The expression of Ki-67 (red) is shown in tumor cells from *Lcre;NICD1* mice infected with viruses expressing GMNC-myc. GMNC-myc (green) labels infected cells. DAPI staining (blue) labels nuclei. Scale bar, 20 µm. Quantification of Ki-67 expression in tumor cells from *Lcre;NICD1* mice infected with viruses expressing GMNC-myc or control vectors is shown in the lower panel (*n* = 5 per treatment, mean ± s.e.m., paired *t*-test, ****P < 0.0001). Data represent at least three independent experiments. **E** Representative images of *Gmnc* expression (green) by RNAscope are shown in tumor cells treated with vehicle or IMR-1. DAPI staining (blue) labels nuclei. Scale bar, 20 µm. Quantification of *Gmnc* transcript is shown (*n* = 7 per treatment; mean ± s.e.m., two-tailed unpaired *t*-test, ***P < 0.001). Three independent experiments were conducted. **F** The expression of ARL13B (red) is shown in *Gmnc*-deficient tumor cells treated with vehicle or IMR-1/IMR-1A. GFP (green) labels tumor cells. DAPI staining (blue) labels nuclei. Scale bar, 20 µm. Data represent five independent experiments. **G** RT-qPCR analysis of *Foxj1* expression in tumor cells treated with vehicle or IMR-1 (tumors from *Lcre;NICD1* mice: *n* = 6 per treatment; tumors from *Lcre;NICD1;Gmnc*^*flox/−*^ mice: *n* = 4 per treatme*n*t, mean ± s.e.m., paired *t*-test, *P < 0.05, NS, not significant). Results were obtained from three independent experiments. **H** Quantification of Ki-67 expression is shown in *Gmnc*-deficient tumor cells treated with vehicle or IMR-1 (*n* = 6 per treatment, mean ± s.e.m., two-tailed unpaired *t*-test, NS not significant). Three independent experiments were conducted.

To understand the mechanisms of MCC regulation in tumor cells, we integrated RT-qPCR, RNAseq, and spatio-temporal gene expression data. Results from these assays consistently showed that both *Foxj1* and *Mcidas* were expressed in tumor cells at lower levels than observed in wild type CP epithelium (Fig. 7A; Supplementary Fig. S11A–D) [34]. As this suggested that upstream regulators of the MCC program were impaired, we examined *Gmnc* expression. Although *Gmnc* exhibited ubiquitous expression in the CP epithelium, we consistently observed decreased levels of *Gmnc* and its downstream target *TAp73* in CP tumors (Fig. 7A, B; Supplementary Fig. S11A, C, D). This was accompanied by a transient increase in the expression of *Gmnn*, a gene that is normally associated with proliferation and was shown to antagonize GMNC transcriptional functions (Fig. 7B) [15, 30]. These results demonstrate that the GMNC-MCIDAS program is profoundly repressed in NOTCH-driven CP tumors, and this can be modulated using NOTCH pathway inhibitors.

To understand the role of the GMNC-MCIDAS program in defective multiciliation of CP tumors, myc-tagged GMNC or MCIDAS was expressed in tumor cells from *Lcre;NICD1* mice using viral vectors. Enforced expression of *Gmnc* or *Mcidas* led to the formation of multiple cilia and reduced proliferation in infected tumor cells within 72 h (Fig. 7C, D; Supplementary Fig. S12A, B), phenocopying NOTCH inhibition with IMR-1 that significantly increased *Gmnc* levels in tumor cells (Fig. 7E). We subsequently eliminated *Gmnc* by crossing *Gmnc*^*flox/−*^ and *Lcre;NICD1* animals. Tumor cells from *Lcre;NICD1;Gmnc*^*flox/−*^ mice became resistant to multiciliation, *Foxj1* activation, and decreased proliferation induced by IMR-1/IMR-1A (Fig. 7F–H; Supplementary Fig. S12C, D). Conversely, overexpression of *Gmnc* increased *Foxj1* expression in tumor cells (Supplementary Fig. S12E). Therefore, these results indicate that monociliation in tumor cells is maintained through NOTCH suppression of GMNC-MCIDAS signaling and suggest that GMNC loss prevents the rescue of multiciliation defects by NOTCH inhibition.

Similar to *Rb1/Trp53*-deficient CPC, despite the suppression of the GMNC-MCIDAS program by NOTCH, combined loss of *Gmnc* and *Patched1* failed to induce CPC in *Lcre;Ptch*^*cko*^*;Gmnc*^*flox/−*^ mice (Supplementary Fig. S13; Supplementary Table 1), suggesting that loss of GMNC-driven multiciliation in the CP is insufficient to replace NOTCH or *Rb1/Trp53* deletion in CPC. Together, these data indicate that GMNC-MCIDAS program deficiencies critically mediate cilia defects in CPC to modulate tumor growth.

## Discussion

CPC clinical outcomes remain dismal, leaving patients vulnerable to devastating consequences [2, 3]. The gross genomic alterations in CP tumors have made the identification of driving events and actionable targets difficult [43, 44]. The GMNC-MCIDAS program promotes multiciliogenesis in different tissues, is required for MCC generation in mice, and mutations in both *GMNC* and *MCIDAS* have been identified in human ciliopathies [13, 14]. The observation that there is consistent disruption of multiciliogenesis program and prevalence of solitary cilia in CPC indicates that CPC has characteristics of a ciliopathy and that therapeutic strategies aimed at restoring multiciliogenesis may suppress CP tumors.

**Fig. 8:**
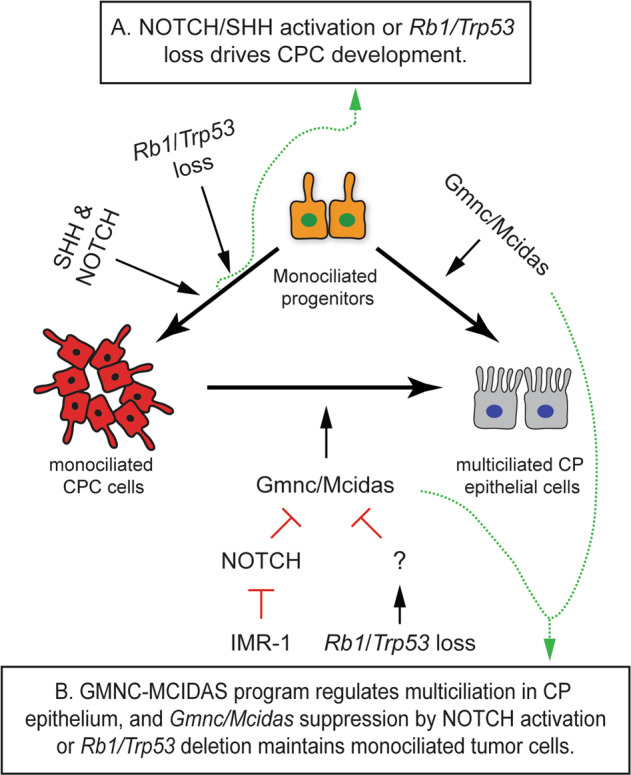
Schematic diagram of GMNC-MCIDAS program in CP development and tumorigenesis. **A** Combined activation of NOTCH and SHH signaling, or loss of *Rb1*/*Trp53* tumor suppressors drives CPC development. **B** GMNC-MCIDAS program mediates multiciliation in CP epithelium, and is repressed by NOTCH signaling in roof plate progenitors, whereas NOTCH inhibitor IMR-1 promotes GMNC-dependent multiciliation and suppresses tumor growth. GMNC-MCIDAS program suppression in *Trp53*-deficient CPC maintains monociliated tumor cells.

Our findings revealed the interaction of the multiciliogenesis program, NOTCH, and SHH pathways during CP differentiation and tumorigenesis. NOTCH suppressed multiciliation of roof plate progenitors, thereby preserving cilia-based signaling activated by SHH from postmitotic MCCs in CP epithelium [33]. Conversely, SHH signaling enhanced *Hes1* and *Hes5* expression in the roof plate in *Lcre;Ptch*^*cko*^ mice and NOTCH-driven CP tumors. The expanded upper roof plate in *Lcre;Ptch*^*cko*^*;NICD1* mice is consistent with the developmental origin and cilia defect of CPC being driven by NOTCH and SHH signaling. These animals represent an ideal therapeutic model for congenital or infantile CPC, a rare condition associated with high morbidity and mortality [50, 51]. Indeed, NOTCH inhibition by IMR-1 rescued the cilia deficit by inducing multiciliated tumor cells, whereas SHH pathway inhibitors suppressed tumor cell proliferation (Fig. 8) [34]. Thus, further study of the interactions between the SHH and NOTCH pathways in CP tumors is warranted to determine the therapeutic potential of activators of multiciliation and cilia-dependent signaling [52–56].

While the differentiation of MCCs requires NOTCH inhibition, it is unclear precisely how NOTCH impacts MCC fate during tumorigenesis [57–61]. Our data shows for the first time that NOTCH suppresses the expression of *Gmnc* and *Mcidas* to impair multiciliation during tumorigenesis. *Gmnc* is required for MCC differentiation following NOTCH inhibition, indicating that GMNC-MCIDAS signaling is required downstream of NOTCH regulation and represents a potent anti-tumor mechanism in CP tumors (Fig. 8).

Consistent with previous studies, we found that the GMNC-MCIDAS program was required for multiciliation in the CP epithelium. As progenitor cells exit the cell cycle to undergo multiciliogenesis, the expression of *Gmnc*, *Mcidas*, *Foxj1*, and *TAp73* was upregulated, as has been observed in other multiciliated tissues [26]. Ectopic expression of either GMNC and MCIDAS stimulated *Foxj1* and *TAp73* expression, whereas *Gmnc* loss prevented the activation of *Mcidas*, *TAp73*, and *Foxj1*, in contrast to *Mcidas-*deficient MCCs that showed expression of both *Foxj1* and *TAp73* [14, 25]. Although both *TAp73* and *Foxj1* are sensitive to *Gmnc* status, loss of *TAp73* failed to affect *Foxj1* expression in the CP, as it does in other MCCs, indicating that *TAp73* is not integrated into the *Gmnc*-*Foxj1* axis in the CP [26]. Consistent with this, TAp73 expression varied greatly in *Rb1/Trp53*-deficient CPC in mice, as well as CP tumors in humans. These results highlight the need to further analyze the similarities and differences between different MCC types.

Overall, this study shows that the GMNC-MCIDAS program is required for MCC differentiation in the CP. The impairment of the program by oncogenic signals including *Rb1/Trp53* defects or NOTCH activation prevents multiciliation and facilitates proliferation of CP tumor cells (Fig. 8). Therefore, activation of multiciliogenesis may serve as a potential therapeutic strategy in a subset of CP tumors. As the early events leading to the activation of the GMNC-MCIDAS program remain poorly characterized, a detailed understanding of its regulation and functions will be critical for developing strategies to target this pathway for the treatment of CPC.

## Materials and methods

### Animals

*Gt(ROSA)26Sor*^*tm1.Notch1Dam*^*/J* (*Rosa26-NICD1*) mice, B6N.129-*Ptch1*^*tm1Hahn*^/J (*Ptch*^*flox/flox*^) mice, B6.129P2-*Trp53*^*tm1Brn*^/J (*Trp53*^*flox/flox*^) mice, *Rb1*^*tm2Brn*^/J (*Rb*^*flox/flox*^) mice, and *C57BL/6* mice (all from Jackson Laboratory, Bar Harbor, ME, USA), *Tg(Lmx1a-cre)1Kjmi* (*Lmx1a-Cre*) mice, *Gmnc*^*tm1Strc*^ (*Gemc1*^*−/+*^) mice, and *Gmnc*^*tm1.1Strc*^ (*Gemc1*^*flox/+*^) mice were maintained by breeding with C57BL/6 mice (Supplementary Table 1). Animals were housed in the Animal Research Facility at New York Institute of Technology College of Osteopathic Medicine in accordance with NIH guidelines. All animal experimental procedures were approved by Institutional Animal Care and Use Committee (IACUC) and performed in compliance with national regulatory standards. *Mcidas* mutant mice were housed at the Biological Resource Center of the Agency for Science, Technology and Research (A*STAR) of Singapore, and experiments performed with these animals followed guidelines stipulated by the Singapore National Advisory Committee on Laboratory Animal Research. All experimental procedures at the Institute for Research in Biomedicine were conducted following European and National Regulation for the Protection of Vertebrate Animals used for experimental and other scientific purposes (directive 86/609), internationally established 3R principles, and guidelines established by the United Kingdom Coordinating Committee on Cancer Research.

The animal experiments were not randomized, and both male and female animals were used for experiments at different time points. For analysis of the *Gmnc*-*Mcidas* program in MCC differentiation in the CP, animals analyzed included: *Gmnc*^−^^*/*−^ *Mcidas*^−^^*/*−^, and wild type animals (*n* = 3 for each at genotype at each time point). The investigators were blinded to group allocation during experiments and assessment of tumor development in wild type (*n* = 16), *Lmx1a-Cre;p53*^*flox/flox*^*;Rb*^*flox/flox*^ (*n* = 23), *Lmx1a-Cre;p53*^*flox/flox*^*;Rb*^*flox/flox*^;*Gmnc*^*−/+*^ (*n* = 17), and *Lmx1a-Cre;p53*^*flox/flox*^*;Rb*^*flox/flox*^;*Gmnc*^*flox/−*^ (*n* = 36) mice. For NOTCH-driven CP tumors, animals analyzed used included: wild type (*n* = 11), *Lmx1a-Cre;NICD1* (*n* = 4), *Lmx1a-Cre;Ptch*^*flox/flox*^ (*n* = 3), and *Lmx1a-Cre;Ptch*^*flox/flox*^*;NICD1* (*n* = 8) mice. For analysis of the role of *Gmnc* in NOTCH-driven CP tumors, 3 animals were used for wild type, *Lmx1a-Cre;Ptch*^*flox/+*^*;Gmnc*^*flox/−*^, *Lmx1a-Cre;Ptch*^*flox/flox*^*;Gmnc*^*flox/−*^ mice. Experimental animals were administered 15 mg/kg IMR-1 (SML1812, Sigma-Aldrich, St. Louis, MO, USA) or vehicle by intraperitoneal injection of pregnant females. Animals used included: wild type (*n* = 3 for vehicle or IMR-1, respectively), *Lmx1a-Cre;NICD1* (vehicle *n* = 14; IMR-1: *n* = 13), and *Lmx1a-Cre;Ptch*^*flox/flox*^*;NICD1* (*n* = 5 for vehicle or IMR-1, respectively).

### Human samples

CP specimens were procured with informed consent from patients following the requirements by institutional review boards at Shanghai East Hospital, Sanford Burnham Prebys Medical Discovery Institute, and University Medical Center Hamburg-Eppendorf. All CP specimens from Boston Children’s Hospital were obtained under an approved institutional review board protocol. All tissues were handled in accordance with guidelines and regulations for the research use of human brain tissue set forth by the NIH (http://osp.od.nih.gov/o_ce-clinical-research-and-bioethics-policy). Diagnoses of human CP specimens from Boston Children’s Hospital were reviewed by two neuropathologists (HGWL, S. Santagata) using standard WHO criteria [62].

### Cell culture

Multiple sets of tissue specimens were collected from animals of appropriate genotype and maintained in culture. Gender information is not available for animals collected at day P7. Primary CP tumor cells were cultured as described previously [34]. Dissected specimens were dissociated and digested at 37 °C for 20 min with pronase (2 mg/ml, 537088, Calbiochem, San Diego, CA, USA) in Hank’s balanced salt solution (14170-112; Thermo Fisher Scientific, Waltham, MA, USA) supplemented with 2 mM glucose. Dissociated tumor cells were centrifuged at 100 *g* for 2 min at 4 °C. Cells were resuspended and cultured in Dulbecco’s modified Eagle’s medium/Nutrient Mixture F-12 Ham’s-Liquid Media (DMEM:F12, SH30261; HyClone Laboratories, Waltham, MA, USA) supplemented with 10% fetal bovine serum (FBS, R&D Systems, Inc., Minneapolis, MN, USA) and 100 IU/mL penicillin/streptomycin (Thermo Fisher Scientific). Cultured cells were treated with ShhN, with vehicle or IMR-1 (25 μM). HEK293 human embryonic kidney cells (ATCC, CRL-1573, Manassas, VA, USA), and AD-293 cell line (Agilent Technologies, Santa Clara, CA, USA) were cultured in Dulbecco’s modified Eagle’s medium (DMEM; 5.5 mM glucose, Thermo Fisher Scientific) supplemented with 10% FBS (R&D Systems, Inc.), and 100 IU/ml penicillin/streptomycin (Thermo Fisher Scientific). Mouse inner medullary collecting duct cells (mIMCD3, ATCC, CRL-2123) were cultured in DMEM:F12 Medium (HyClone Laboratories) supplemented with 10% FBS (R&D Systems, Inc.), and 100 IU/mL penicillin/streptomycin (Thermo Fisher Scientific). These cells were maintained in a humidified atmosphere with 5% CO2 at 37 °C in a cell culture incubator.

Analyses of gene expression, cell proliferation, and signal transduction were performed using CP tumor cells from *Lmx1a-Cre;NICD1* (*n* = 13), *Lmx1a-Cre;NICD1;Gmnc*^*flox/−*^ (*n* = 8), and *Lmx1a-Cre;p53*^*flox/flox*^*;Rb*^*flox/flox*^;*Gmnc*^*flox/−*^ mice (*n* = 3). Results from these studies confirmed their identity (Figs. 3E, 5B–E, 7C–H; Supplementary Figs. S4B–D, S7C, D, S10A, B, S11D, S12A–E). HEK293, AD-293, and mIMCD3 cells were tested regularly for mycoplasma. Given the short time (<8–10 days) during which CP cells were maintained as primary cultures supplemented with antibiotics, we did not test for mycoplasma contamination.

### Viruses

PmeI-linearized pShuttle-vectors carrying different cDNA fragments were introduced into the replication-deficient adenoviral vector pAdEasy-1 through homologous recombination in BJ5183 cells (Agilent Technologies). Successfully recombined adenoviral vector was verified by sequencing. The adenoviral plasmid was linearized by PacI digest and transfected into AD-293 cells (Agilent Technologies) to produce recombinant viral particles. All the procedures of production, purification, and use of adenoviruses were approved by Institutional Biosafety Committee.

### Immunohistochemistry, immunofluorescence, and immunocytochemistry

Multiple sets of tissue specimens were collected from animals. Gender information is not available for animals collected during embryonic development and at days P0 and P7. Immunostaining was carried out as previously described [34]. Primary antibodies used and dilution ratios are: mouse monoclonal anti-Acetylated α-Tubulin (1:500, ab24610, clone 6-11B-1, abcam, Cambridge, MA, USA), mouse monoclonal anti-Acetylated α-Tubulin (1:500, T7451, clone 6-11B-1, Sigma-Aldrich), mouse monoclonal anti-ARL13B (1:500, clone N295B/66, NeuroMab, Davis, CA, USA), rabbit anti-ARL13B (1:500, 17711-1-AP, Proteintech, Chicago, IL, USA), mouse monoclonal anti-γ-Tubulin (1:10000, T6557, clone GTU-88, Sigma-Aldrich), chicken anti-GFP (1:1000, GFP-1010, Aves Lab, Tigard, OR, USA), rabbit monoclonal anti-Ki-67 (1:100, clone SP6, ab16667, abcam), mouse monoclonal anti-Aquaporin 1 (1:1000, clone 1/22, ab9566, abcam), rabbit anti-Aquaporin 1 (1:1000, AB2219, MilliporeSigma, Burlington, MA, USA), and rabbit anti-OTX2 (1:500, AB9566, MilliporeSigma), rabbit anti-Cytokeratins (1:100, Z0622, Dako, Carpinteria, CA, USA), mouse monoclonal anti-FOXJ1 (1:50, 14-9965, Clone 2A5, eBioscience, San Diego, CA, USA), and rabbit anti-TAp73 (1:200, ab40658, abcam), and sheep anti-Transthyretin (1:200, ab9015, abcam), and goat anti-Myc (1:1000, ab9132, abcam).

The investigators were blinded to group allocation in the following experiments. For analysis of TAp73 expression in human tissue samples, 9 CPPs, 8 CPCs, and 5 CPs were used. Four distinct tissue regions and 5 fields of view in each region were assessed to determine the average scores for each sample: percent positive fields of view were calculated by scoring each 40× field as “1” if there were TAp73^+^ cells present and “0” if there were none. For staining of primary cilia in CP tumors, the cilia pattern was assessed by analyzing five distinct tissue regions of each sample. Human tissues used included: 17 CPPs from 16 individuals, 6 CPCs from 6 individuals. Animals examined included: wild type and *Mcidas*^−^^*/*−^ animals, *Lmx1a-Cre;p53*^*flox/flox*^*;Rb*^*flox/flox*^, *Lmx1a-Cre;NICD1*, *Lmx1a-Cre;Ptch*^*flox/flox*^, and *Lmx1a-Cre;Ptch*^*flox/flox*^*;NICD1* mice, and wild type, *Lmx1a-Cre;NICD1*, and *Lmx1a-Cre;Ptch*^*flox/flox*^*;NICD1* mice treated with vehicle or IMR-1. For analysis of the expression of *Atoh1*, SOX2, and PAX6 in embryonic cerebellum after treatment with vehicle or IMR-1, wild type animals were used.

For analysis of cell proliferation in tissues, the number of Ki-67^+^ cells in 200 cells was assessed from three distinct regions for each sample. The percentage of Ki-67^+^ cells was determined by calculating the number of Ki-67^+^ cells per 100 cells in each sample for each genotype or each treatment. For proliferation analysis of cultured cells, Ki-67 expression in 100–200 tumor cells or infected cells was assessed by analyzing three distinct fields. The percentage of Ki-67^+^ cells was determined by calculating the number of Ki-67^+^ cells per 100 tumor cells or infected cells in each sample for each treatment.

For analysis of multiciliation of cultured cells, primary cilia in 100–150 tumor cells or infected cells were assessed by analyzing ARL13B or γ-tubulin expression in three distinct fields. The percentage of MCCs was determined by calculating the number of MCCs per 100 tumor cells or infected cells of each sample for each treatment.

### Immunoblotting

Immunoblotting was carried out as described previously [34]. Primary antibodies used included: mouse monoclonal anti-β-Actin (1:1000, clone AC-15, A5441, Sigma-Aldrich), and rabbit anti-TAp73 (1:1000, ab40658, abcam), and mouse monoclonal anti-FLAG (1:1000, clone M2, F3165, Sigma-Aldrich).

### RT-qPCR, in situ hybridization and RNAscope

Multiple sets of tissue specimens were collected from animals. Gender information is not available for animals collected at days P0 and P7. Total RNA was extracted from tumor samples using Trizol (Thermo Fisher Scientific) and RNA Clean & Concentrator kits (Zymo Research, Irvine, CA, USA). Total RNA samples from normal human tissues were purchased (BioChain Institute, Inc., Newark, CA, USA). cDNA was synthesized using GoScript Reverse Transcription System (Promega, Madison, WI, USA). All reactions were run on a QuantStudio 3 Real-Time PCR System (Thermo Fisher Scientific). Gene-specific primers and probes were used (*Gdf7*, Mm.PT.58.12400445; *GMNC*, Hs.PT.58.15454001; *MCIDAS*, Hs.PT.58.22676071; *FOXJ1*, Hs.PT.58.40371261, Integrated DNA Technologies, Inc., Coralville, IA, USA) (Supplementary Table 2) [34]. Transcript levels were determined as the number of transcripts of genes of interest relative to those of *Actb* (mouse) or *GAPDH* (human) and normalized to the mean value of control samples. The results for each set of specimens were obtained by averaging transcript levels of technical triplicates and used for subsequent analyses. Exclusion was applied when one of the triplicates was a significant outlier, and the assay was repeated in independent experiments to validate the exclusion. For analysis of *GMNC, MCIDAS,* and *FOXJ1* expression, human samples used included 10 CPPs, 8 CPCs, and 1 for brain, trachea, lung, testis, and epididymis. For analysis of *Gmnc*-deficient CP samples, animals included: 11 *Lmx1a-Cre;Gmnc*^*flox/−*^ and wild type animals, respectively. For CP tumor analysis, samples used included: wild type CP: *n* = 10; CPC: *n* = 11 from *Lmx1a-Cre;p53*^*flox/flox*^*;Rb*^*flox/flox*^ mice, *n* = 10 for *Lmx1a-Cre;p53*^*flox/flox*^*;Rb*^*flox/flox*^;*Gmnc*^*flox/−*^ animals. For NOTCH-driven CP tumors, animals examined included: wild type, *Lmx1a-Cre;NICD1* (*n* = 3 for each at genotype at each time point). For gene expression analysis of infected cells, 3 independent samples for infected and control cells were used, respectively.

In situ hybridization was performed as described at the In Situ Hybridization Core facility at Baylor College of Medicine [63]. Riboprobes for *Gli1*, *Mycn*, *Shh*, *Hes1*, *Hes5*, *Gmnc*, *Mcidas*, and *Foxj1* were used. For RNAscope, *Gmnc* (510421), *Mcidas* (510401), *Myb* (510411), *Ccno* (546521), *Foxj1* (317091), *GMNC* (566231), and *FOXJ1* (430921) probes were used according to manufacturer’s instructions (Advanced Cell Diagnostics, Newark, CA, USA). For analysis of *GMNC/FOXJ1* expression in human tissue samples, the investigator was blinded to group allocation. Human tissues used included: 31 CPPs from 31 individuals; 11 CPCs from 11 individuals. *GEMC1* and *FOXJ1* expression was assessed in five distinct tissue regions: the percentage of *GEMC1*- or *FOXJ1*-expressing cells was calculated by averaging the numbers of *GEMC1*^+^ or *FOXJ1*^+^ cells per 100 cells in five distinct tissue regions of each specimen. For RNAscope in cultured or infected tumor cells, 7 samples were used for each treatment. mRNA transcript copy number was assessed by counting the number of positive fluorescent spots in 50–100 tumor cells or infected cells in three distinct regions. The transcript levels were determined by averaging the transcript copy numbers of all cells for each treatment.

### Electron microscopy and image acquisition

Transmission electron microscopy was performed as described previously [34]. The investigator was blinded to group allocation. A whole-mount bright field was obtained using a Nikon SMZ1000 Stereomicroscope. Light and fluorescent microscopic images were obtained by a Nikon Eclipse 90i microscope system, a Nikon confocal microscope system A1^+^ (Nikon Instruments, Melville, NY, USA), and a ZEISS LSM 980 with Airyscan 2 confocal microscope (Carl Zeiss Microscopy, LLC, White Plains, NY, USA).

### Statistical analysis and reproducibility

Multiple specimens were collected from independent samples or animals for each treatment or genotype. Pilot studies were conducted, and results from these studies were used to determine the choice of sample size for the experiment. A group size of *n* = 10 (5 experimental, 5 control) will provide 90% power to detect a 22% change in assay results. No randomization was used to determine how samples were allocated to experimental groups. Both male and female animals were used for experiments. Experiments were repeated with similar results to eliminate the effects of gender and age on experimental findings. Information on experiment replication is provided in legends for figures and supplemental figures. Statistical analyses were performed with GraphPadPrism 9.0 (GraphPad Software Inc., La Jolla, CA, USA). All pooled data were expressed as the mean ± standard error of the mean (SEM). Variation within each group of data was examined based on the differences between each data point and the mean of the group. The Kolmogorov–Smirnov test was used to test the normal distribution of the data. Differences between two groups were compared using paired *t*-test or unpaired two-tailed *t*-test. Differences between multiple groups were analyzed with ANOVA followed by Tukey’s multiple comparisons test. Results were considered significant at *P < 0.05; **P < 0.01; ***P < 0.001; ****P < 0.0001.

### Accession numbers

Published data sets of human CP tumors (GSE14098, GSE60886) were downloaded from the GEO database. Hierarchical clustering was performed using Genesis (http://genome.tugraz.at/genesisclient/genesisclient_description.shtml). Pathway analysis using the GeneGoMetaCore Analytical Suite (http://genego.com; GeneGo) was used to score and rank pathways enriched in data sets by the proportion of pathway-associated genes with significant expression values. RNA-seq data (BioProject ID, PRJNA282889) were analyzed.

## Supplementary information

Supplementary figures and legends

Supplementary Table 1

Supplementary Table 2

Original Data File - uncropped images of western blots

Signed - Pre-Authorship form

Reproducibility Checklist

## Data Availability

All data generated or analyzed during this study are included in this published article and its supplementary information files.
