# Supplementary material for: Disruption of GMNC-MCIDAS multiciliogenesis program is critical in choroid plexus carcinoma development
Source: Cell Death Differ. 2022 Mar 23;29(8):1596–610. doi: 10.1038/s41418-022-00950-z (PMC9345885; doi:10.1038/s41418-022-00950-z)
Supplement: Supplementary file 1 — Supplementary figures and legends [file 41418_2022_950_MOESM1_ESM.docx]

**
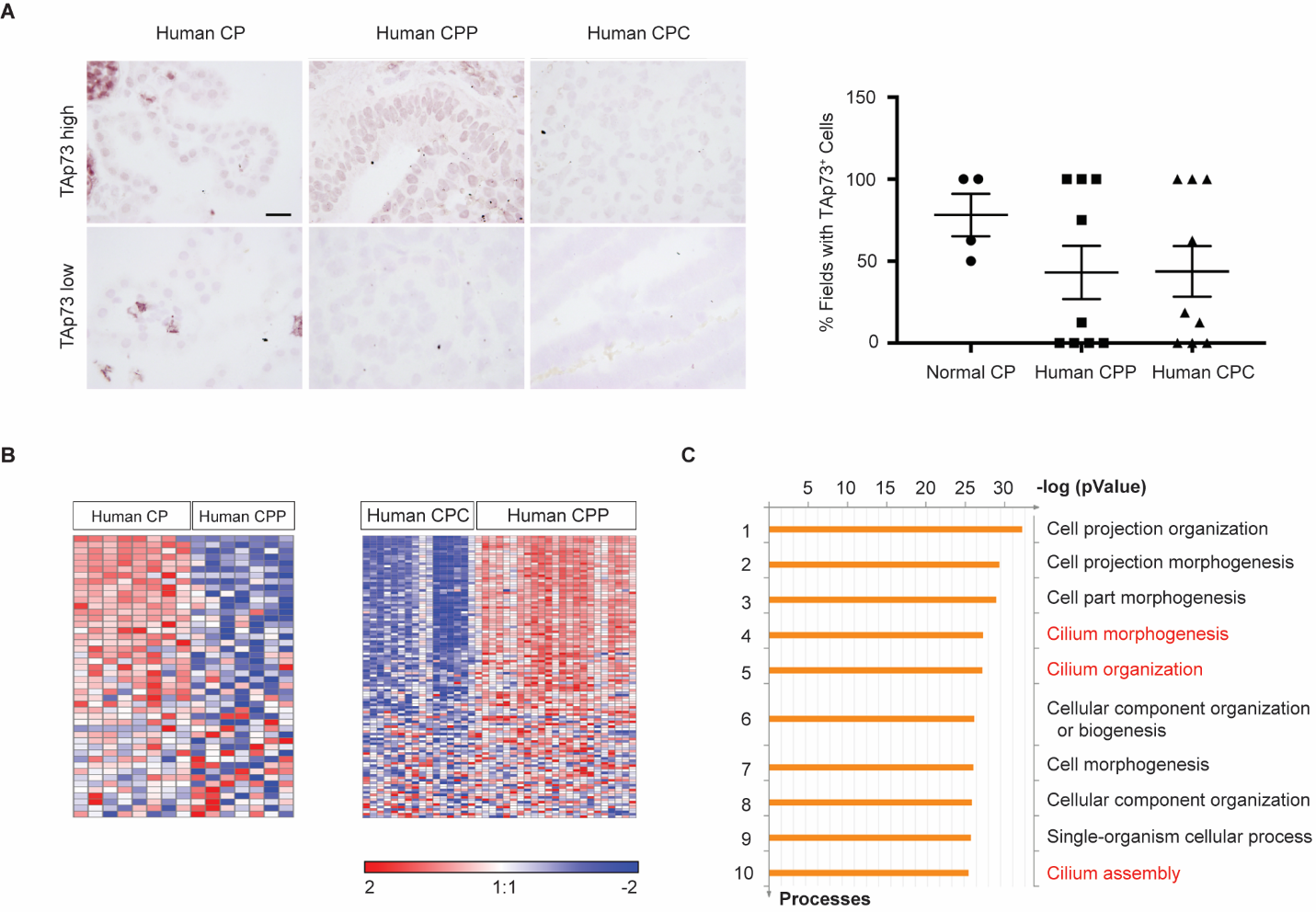
**

**Supplementary Figure S1. Analysis of gene expression in CP tumors in humans. A** Human CP epithelial cells express TAp73, with 50-100% of high magnification fields (100 ×) imaged presenting with TAp73 expression. Human CP tumors displayed variable TAp73 expressions. About half of all CP tumors exhibited TAp73 expression in > 50% of tumor cell population (TAp73 high); the remaining CP tumors display TAp73 expression in small subpopulation of tumor cells (TAp73 low). No difference is observed in TAp73 expression between CPP and CPC. Each point represents one individual and is an average of 4 high-magnification frames across the tumor. Scale bar, 20 µm. Results were obtained from three independent experiments. **B** Left: hierarchical clustering of human CPPs and normal CPs based on 46 genes involved in cilia differentiation (CPP: *n* = 7 tumors from 7 individuals; normal CP: *n* = 8 CPs from 8 individuals; one-way ANOVA, FDR < 0.05, fold change is shown); right: hierarchical clustering of human CPCs and CPPs with 115 genes involved in ciliogenesis (CPP: *n* = 24 tumors from 24 individuals; CPC: *n* = 15 tumors from 15 individuals; one-way ANOVA, FDR < 0.05, fold change is shown). **C** MetaCore gene enrichment analysis of differentially expressed genes between CPPs and CPCs in humans (CPP: *n* = 8 tumors from 8 individuals; CPC: *n* = 15 tumors from 15 individuals). Significantly enriched signaling networks including cilium morphogenesis, organization, and assembly pathways (red) are shown.

**
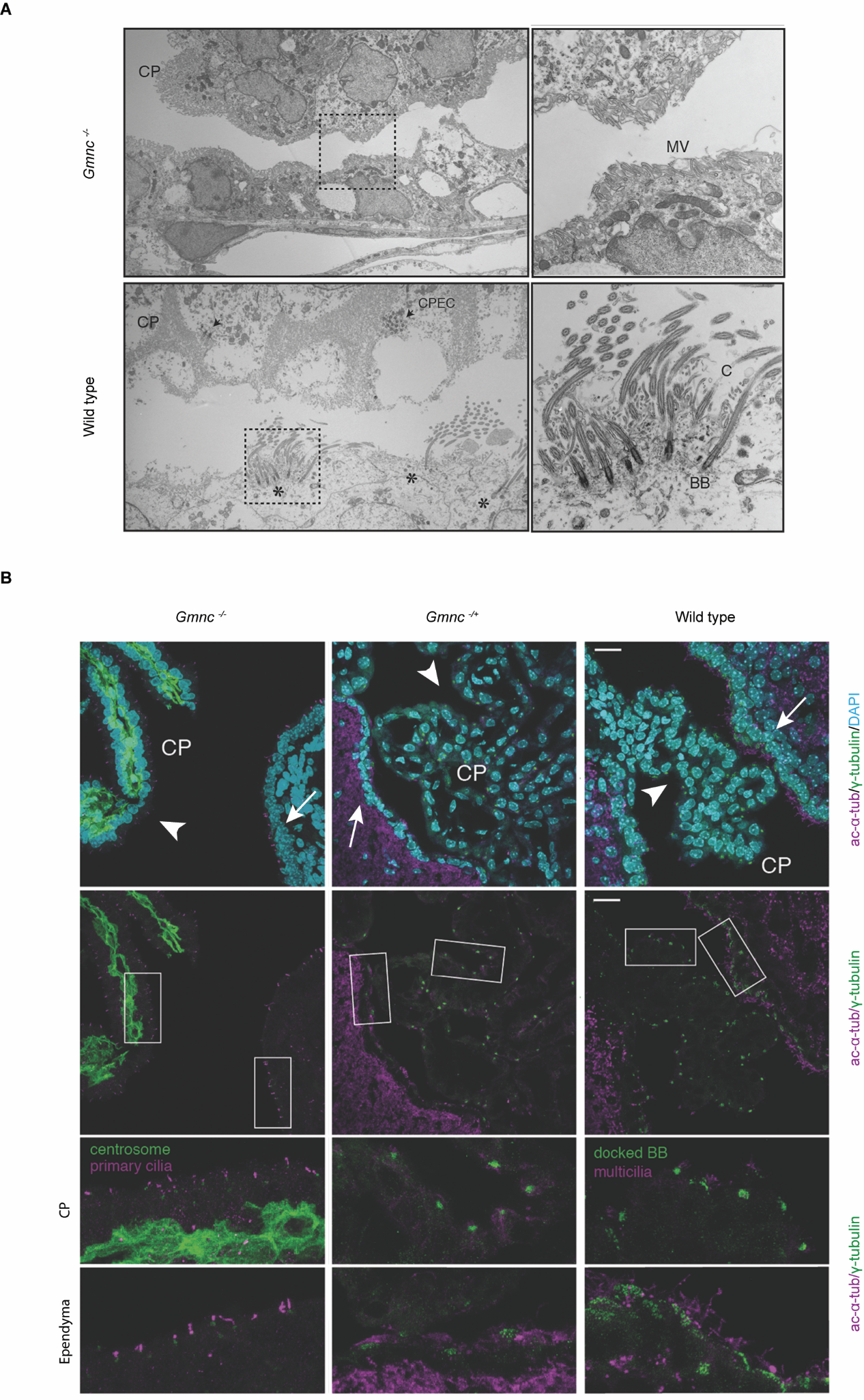
**

**Supplementary Figure S2.** **Loss of multiciliated cells in *Gmnc^-/-^* brain.** **A** Transmission electron micrographs reveal a lack of cilia clusters of CP epithelial cells and motile cilia of ependymal cells at day P9 in *Gmnc^-/-^* animals. In contrast, wild type animals possess multiciliated cells in the CP epithelium (arrows) and ependyma (asterisks). Boxed regions are magnified on the right. CPEC, CP epithelial cells; BB, basal body; C, cilia; MV, microvilli. Images represent three independent experiments. **B** The expression of acetylated α-tubulin (ac-α-tub, magenta) and γ-tubulin (green) is shown in the CP epithelium (arrowheads) and ependyma (arrows) at day P6 in *Gmnc^-/-^* and wild type animals. Boxed regions in the CP and ependyma are shown in higher magnification in lower two panels. DAPI staining (cyan) labels nuclei. BB, basal body. Scale bars, 20 µm. Images are representative of at least three independent experiments.

**
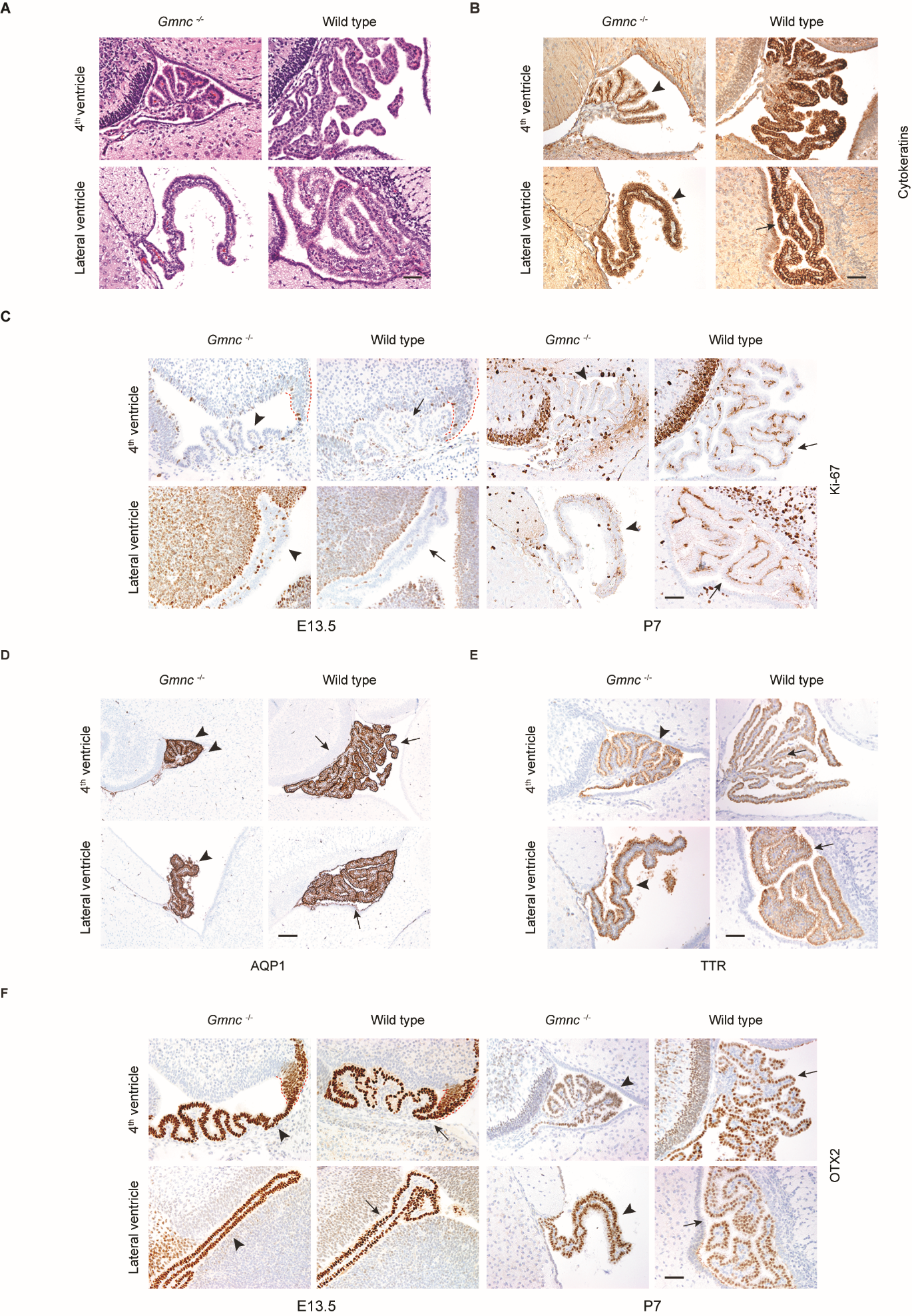
**

**Supplementary Figure S3.** **Analysis of gene expression in *Gmnc^-/-^* CP.** **A** H&E staining of CP in the 4^th^ and lateral ventricles is shown at day P7 in *Gmnc^-/-^* and wild type animals. Scale bar, 50 µm. Images are representative of at least three independent experiments. **B - F** Representative images of immunohistochemical staining for cytokeratins (B), Ki-67 (C), and AQP1 (D), TTR (E), and OTX2 (F) are shown in roof plate (upper roof plate marked by dotted lines) and CP in the 4^th^ and lateral ventricles at day E13.5 (C and F), and day P7 (B-F) in the CP in the 4^th^ and lateral ventricles in *Gmnc^-/-^* (black arrowheads) and wild type (black arrows) animals. Three independent experiments were conducted.


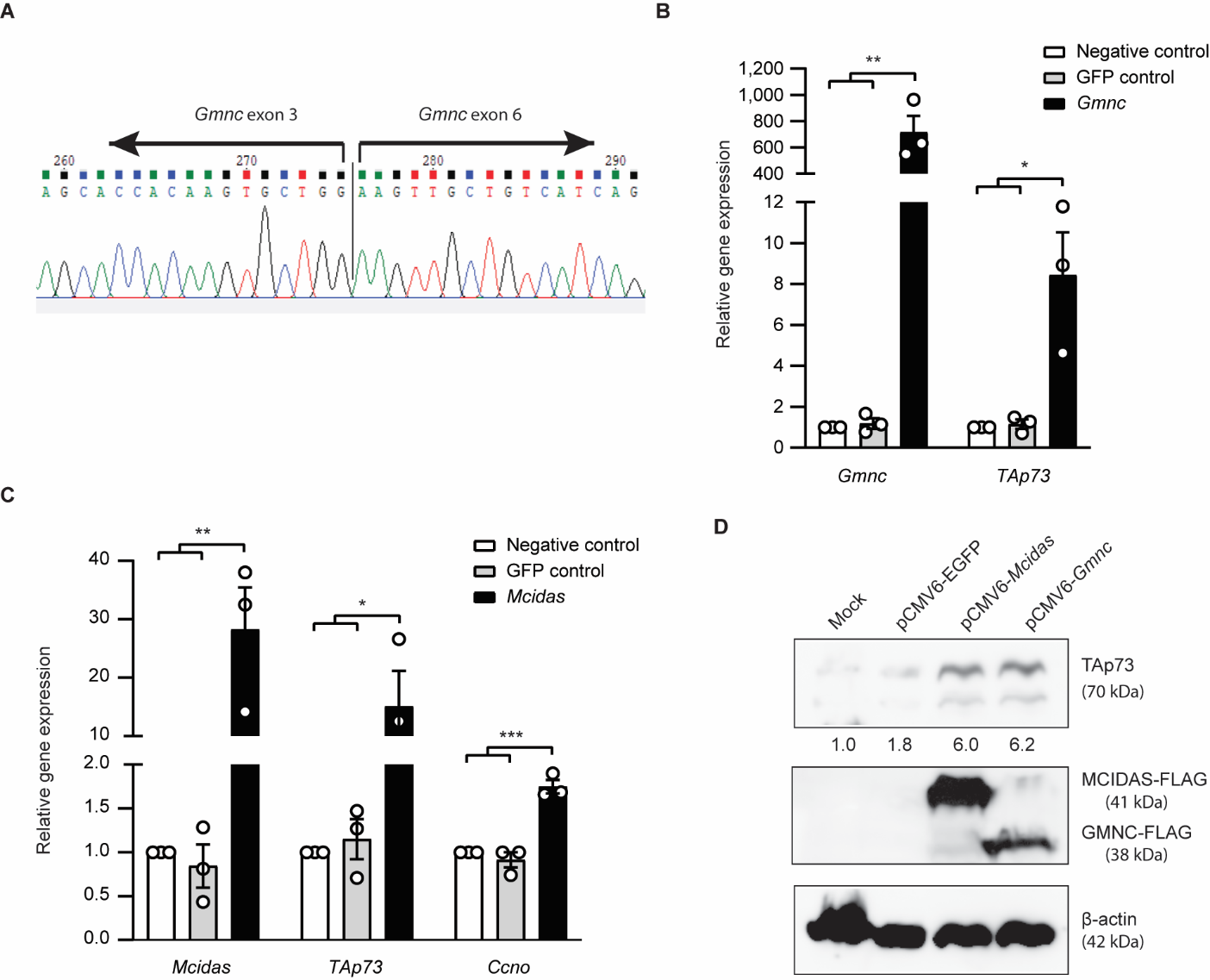


**Supplementary Figure S4.** **Analysis of GMNC-MCIDAS driven gene expression. A** Sequencing trace image of *Gmnc* mutant transcript in the CP of *Lcre;Gmnc^flox/-^* animals. Notice that exon 3 is spliced next to exon 6 in mutant transcript following the deletion of exons 4 and 5. **B**, **C** RT-qPCR analysis of the expression of *Gmnc*, *Mcidas*, *TAp73* and *Ccno* in mouse Inner Medullary Collecting Duct cells infected with viruses expressing GMNC-myc, MCIDAS-myc, or GFP only (*n* = 3 samples per treatment, mean ± s.e.m., one-way ANOVA, **P* < 0.05; ***P* < 0.01; ****P* < 0.001). Three independent experiments were conducted. **D** Immunoblot analysis of HEK293 cells transfected with plasmids expressing FLAG-tagged MCIDAS or GMNC, or GFP only. The expression of GMNC-FLAG, MCIDAS-FLAG, TAp73, and β-actin was shown. The value of each band indicates relative expression level normalized by loading control β-actin. Results were obtained from three independent experiments. Uncropped images of immunoblots are shown in Supplementary Figure S14.


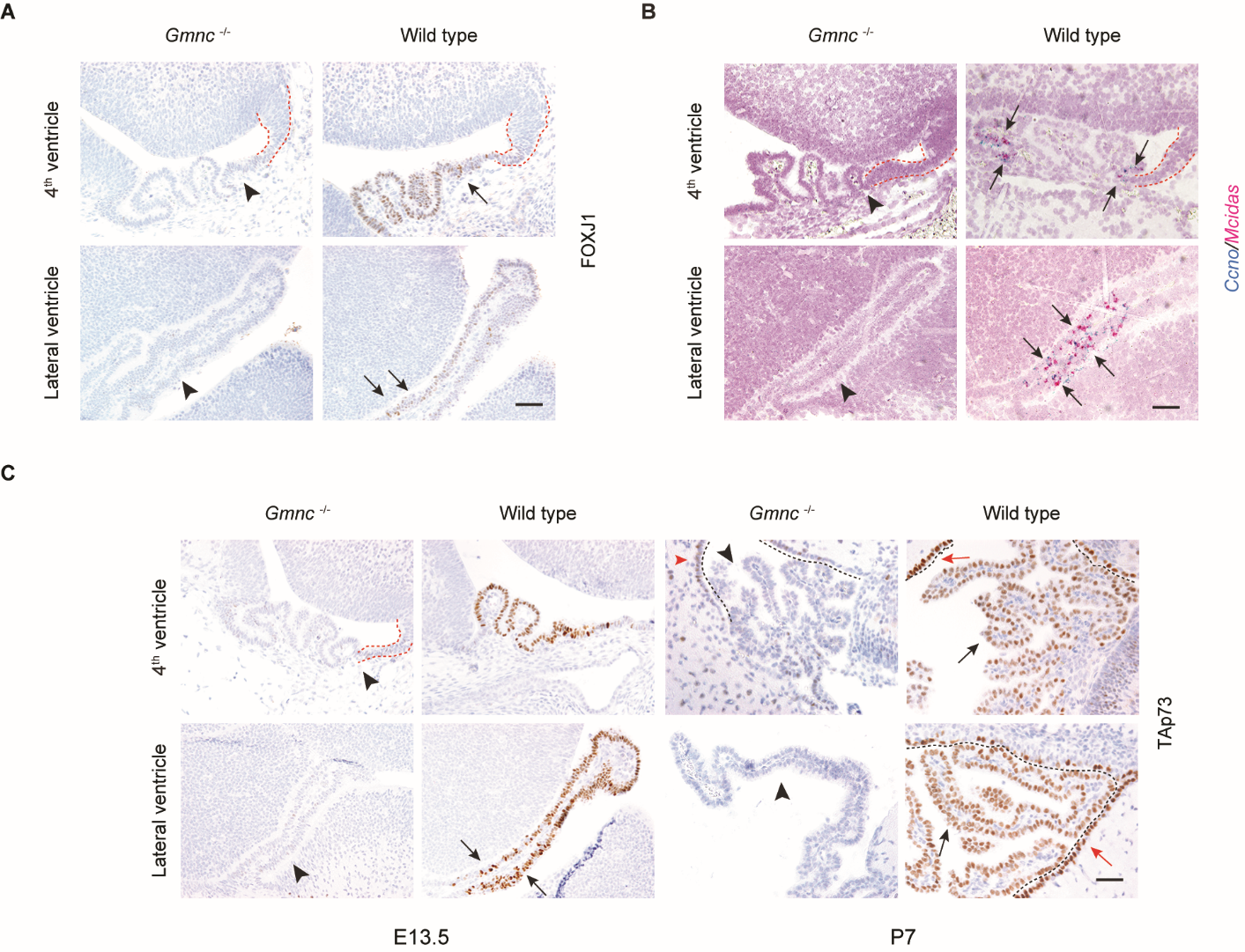


**Supplementary Figure S5.** **Defective multiciliation network in *Gmnc^-/-^* CP.** **A** **- C** Representative images of expression of cytokeratins (A, immunohistochemistry), *Mcidas* and *Ccno* (B, RNAscope), and TAp73 (C, immunohistochemistry) are shown in roof plate (upper roof plate marked by dotted lines) and CP in the 4^th^ and lateral ventricles at day E13.5 (A-C), and day P7 (C) in the CP in the 4^th^ and lateral ventricles in *Gmnc^-/-^* (black arrowheads) and wild type (black arrows) animals. Ependymal cells lining the ventricles are shown in *Gmnc^-/-^* (C, red arrowheads) and wild type (C, red arrows) animals. Scale bars, 50 µm. Three independent experiments were conducted.


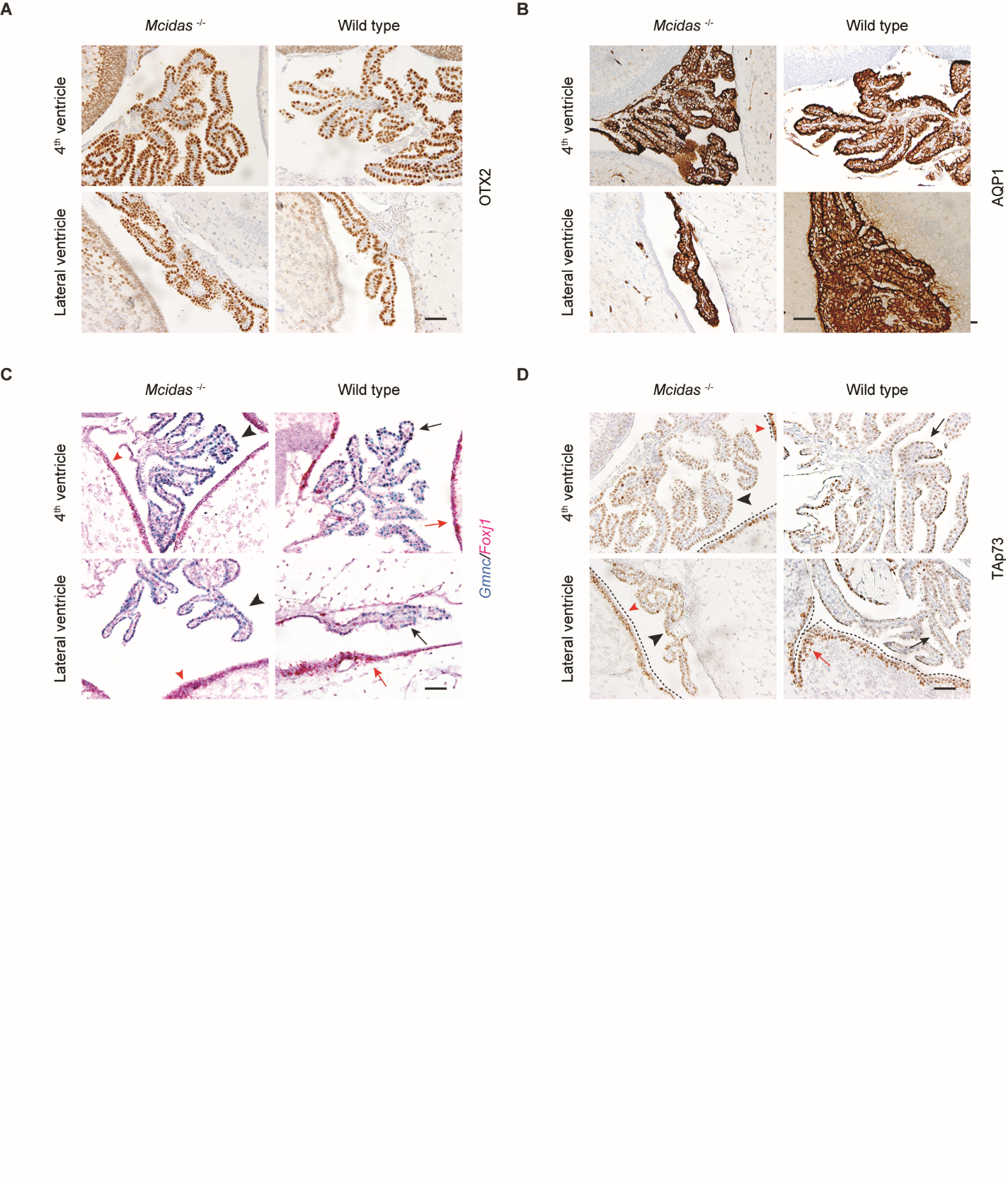


**Supplementary Figure S6.** **Defective multiciliation in *Mcidas*-null CP. A-D** Representative images of the expression of OTX2 (A), AQP1 (B), *Gmnc* and *Foxj1* (C) and TAp73 (D) by immunohistochemistry (A, B, D) and RNAscope (C) are shown at days P7 in the CP in the 4^th^ and lateral ventricles in *Mcidas^-/-^* (black arrowheads, C and D) and wild type (black arrows, C and D) animals. Ependymal cells lining the ventricles (marked by dotted lines) are shown in *Mcidas ^-/-^* (red arrowheads, C and D) and wild type (red arrows, C and D) animals. Scale bars, 50 µm. Results were obtained from three independent experiments.

**
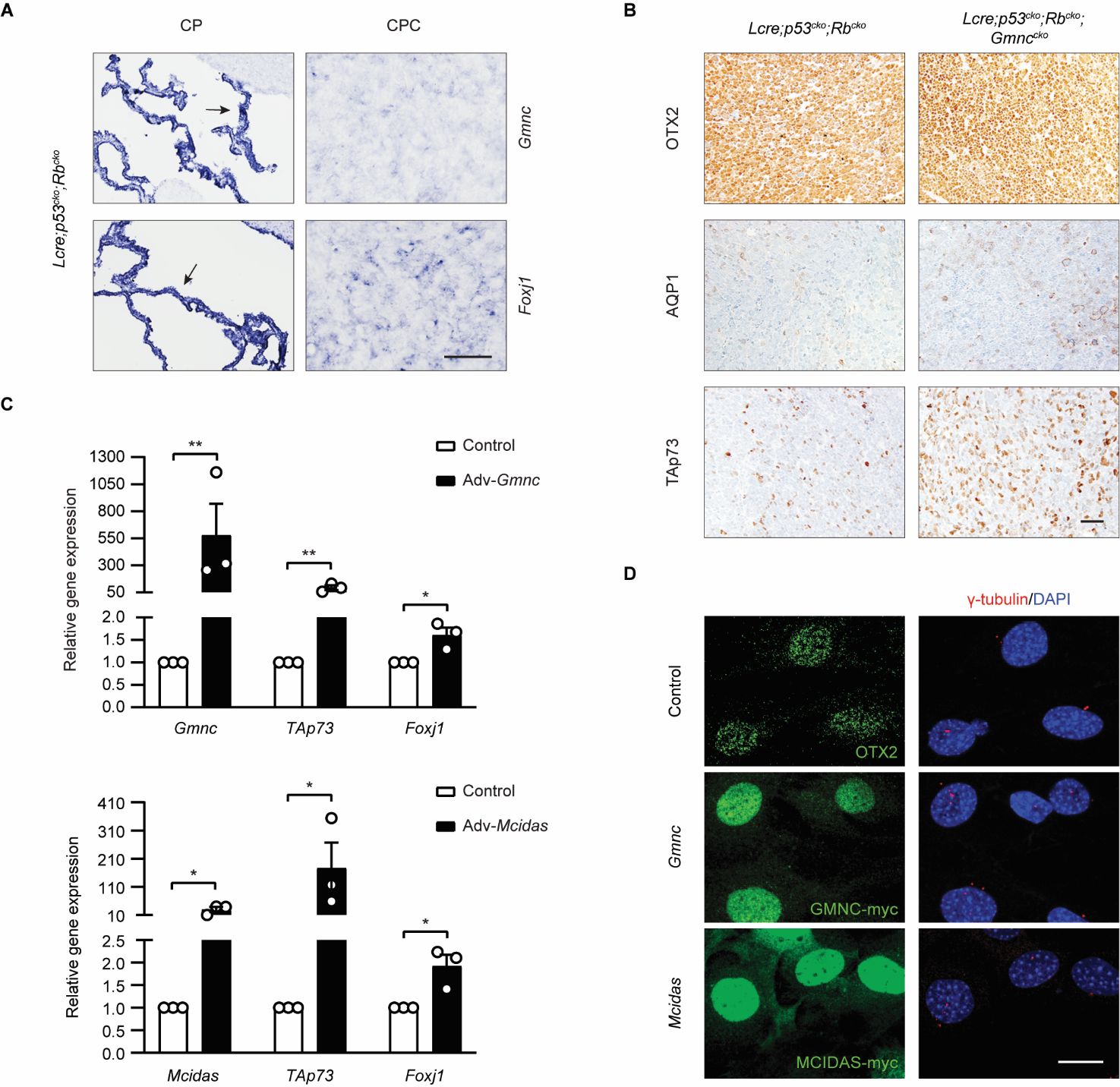
**

**Supplementary Figure S7.** **Analysis of gene expression in *Trp53*-deficient CPC. A**, **B** Representative images of *in situ* hybridization of *Gmnc* and *Foxj1* mRNAs (A), and immunohistochemical staining of OTX2, AQP1, and TAp73 (B) are shown in the CP (A, arrows) and CPC (A, B) from *Lcre;p53^cko^;Rb^cko^* and *Lcre;p53^cko^;Rb^cko^;Gmnc^cko^* animals. Scale bars, 25 µm. Data represent at least three independent experiments. **C** RT-qPCR analysis of tumor cells from *Lcre;p53^cko^;Rb^cko^*;*Gmnc^cko^* animals infected with control viruses, or viruses expressing GMNC-myc, or viruses expressing MCIDAS-myc (*n* = 3 samples per treatment, mean ± s.e.m., paired *t*-test, **P* < 0.05; ***P* < 0.01). Data represent three independent experiments. **D** The expression of ARL13B (red) is shown in tumor cells from *Lcre;p53^cko^;Rb^cko^*;*Gmnc^cko^* infected with viruses expressing GMNC-myc or MCIDAS-myc. OTX2 (green) or myc (green) labels tumor cells. DAPI staining (blue) labels nuclei. Scale bars, 20 µm. Three independent experiments were conducted.

**
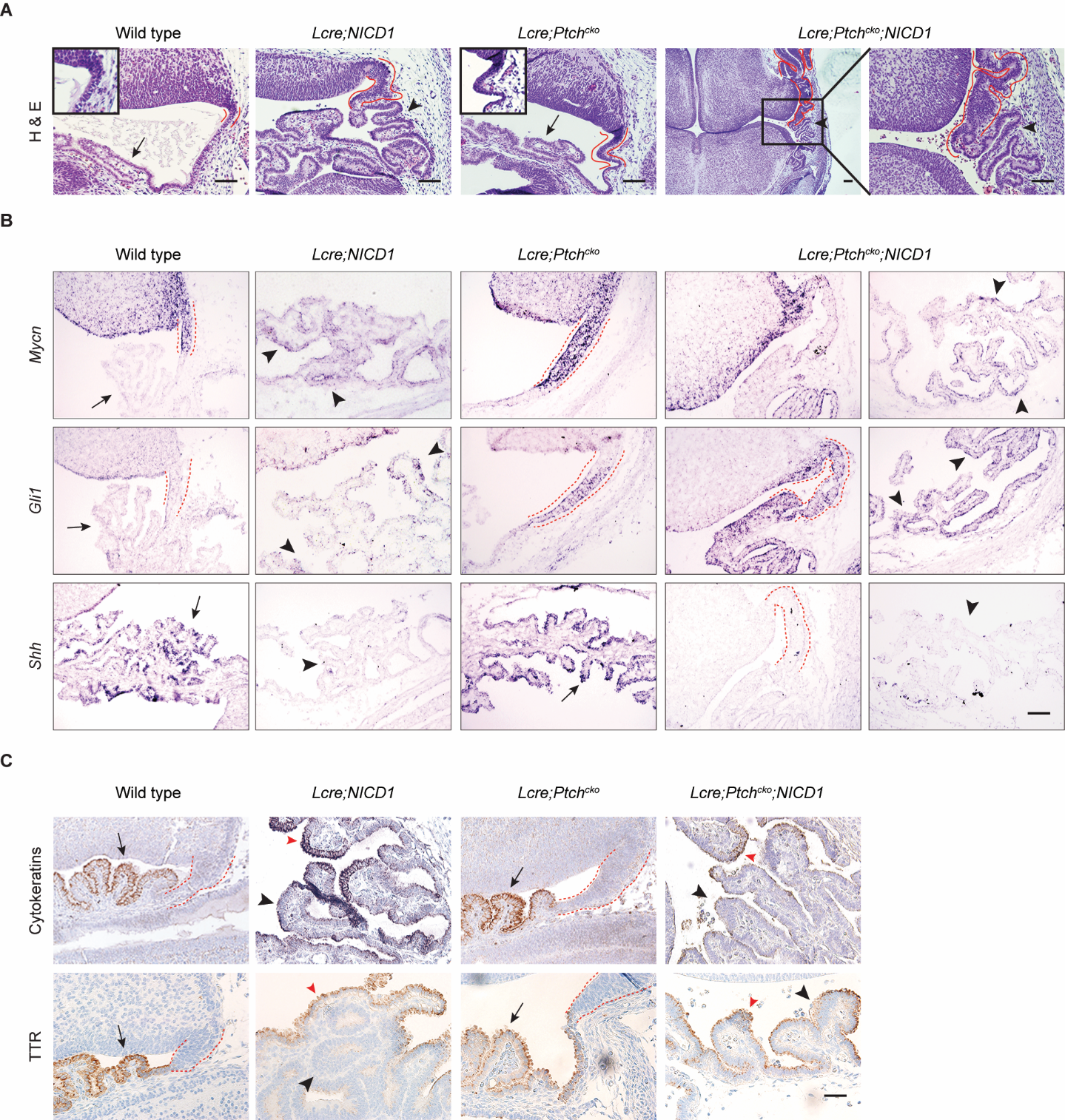
**

**Supplementary Figure S8.** **Morphology and gene expression analysis of CP tumors driven by aberrant NOTCH and SHH signaling.** **A** H&E staining is shown of coronal sections of upper roof plate/CP (arrows) in the hindbrain at day E14.5 in wild type and *Lcre;Ptch^cko^* animals, CPP and abnormal CP growth (arrowheads) in *Lcre;NICD1* and *Lcre;Ptch^cko^;NICD1* mice, respectively. Red lines mark the roof plate magnified in inset images. Boxed region of roof plate/CP in a *Lcre;Ptch^cko^;NICD1* animal is shown in higher magnification on the right. Scale bars,100 µm. Images represent at least three independent experiments. **B, C** Representative results of *in situ* hybridization of *Mycn*. *Gli1* and *Shh* mRNAs (B) and immunohistochemical staining cytokeratins and TTR (C) are shown in upper roof plate (marked by dotted lines) and CP (arrows) at day E14.5 in the hindbrain in wild type and *Lcre;Ptch^cko^* animals, CPP and abnormal CP growth (black arrowheads) in *Lcre;NICD1* and *Lcre;Ptch^cko^;NICD1* animals, respectively. Cytokeratins-expressing and TTR^+^ epithelial cells (C, red arrowheads) are mixed in abnormal CP growth in *Lcre;NICD1* and *Lcre;Ptch^cko^;NICD1* animals, respectively. Scale bars, 50 µm. Data represent at least three independent experiments.

**
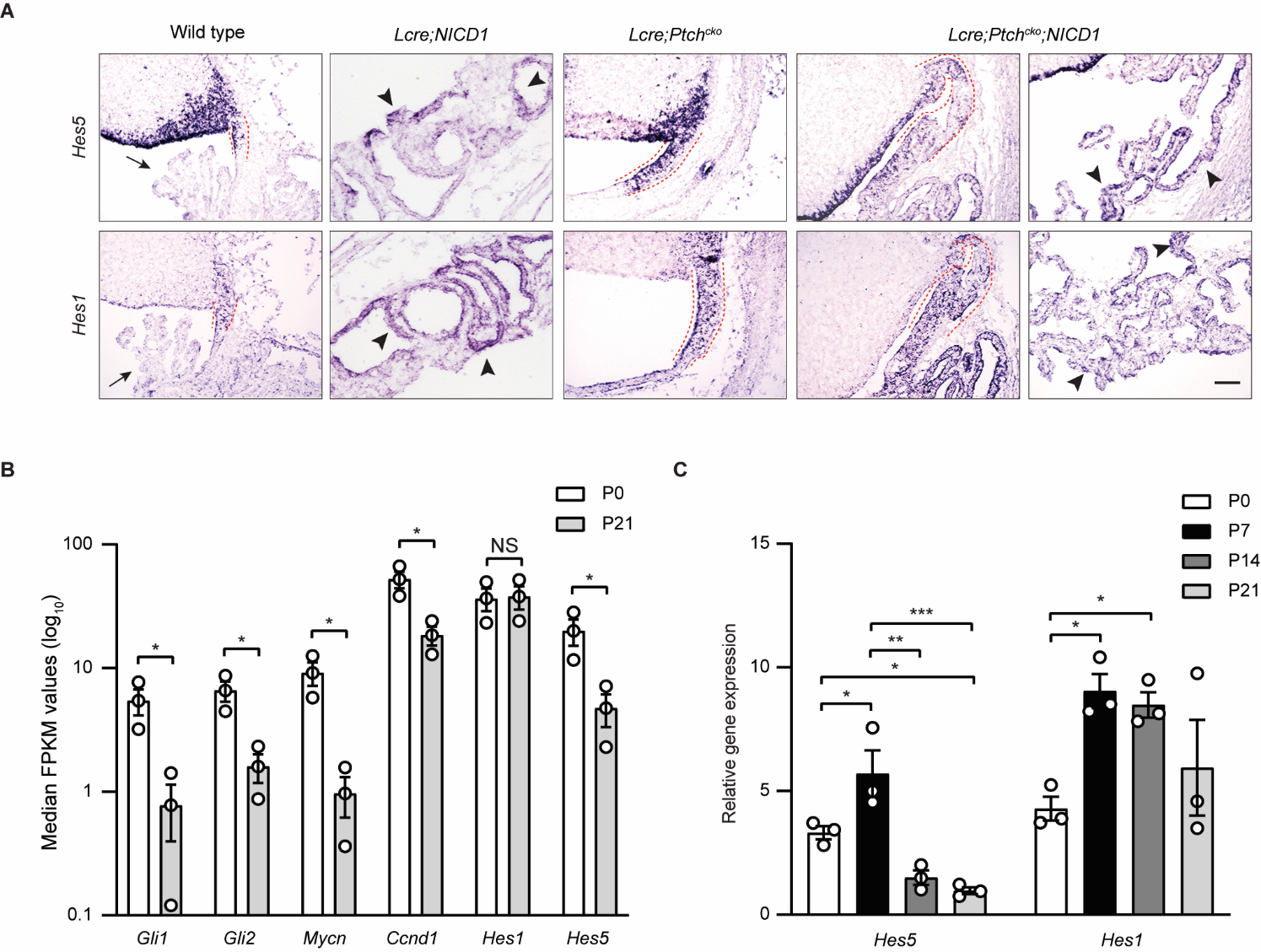
**

**Supplementary Figure S9.** **Analysis of gene expression in CP tumors driven by aberrant NOTCH and SHH signaling.** **A** Representative images of *in situ* hybridization of *Hes1* and *Hes5* are shown in upper roof plate (marked by dotted lines) and CP (arrows) in the hindbrain at day E14.5 in wild type and *Lcre;Ptch^cko^* animals, CPP and abnormal CP growth (arrowheads) in *Lcre;NICD1* and *Lcre;Ptch^cko^;NICD1* animals, respectively. Scale bar, 50 µm. Results were obtained from at least three independent experiments. **B** Median FKPM (fragments per kilobase of exon per million reads mapped) values of genes in CPP in *Lcre;NICD1* mice at day P0 and P21 (*n* = 3 specimens per time point, mean ± s.e.m., two-tailed unpaired *t-*test, **P* < 0.05, NS, not significant). **C** RT-qPCR analysis of gene expression in CPP in *Lcre;NICD1* mice at days P0, P7, P14, and P21 (*n* = 3 animals per time point, mean ± s.e.m., one way ANOVA, **P* < 0.05, ***P* < 0.01, ****P* < 0.001). Three independent experiments were conducted.


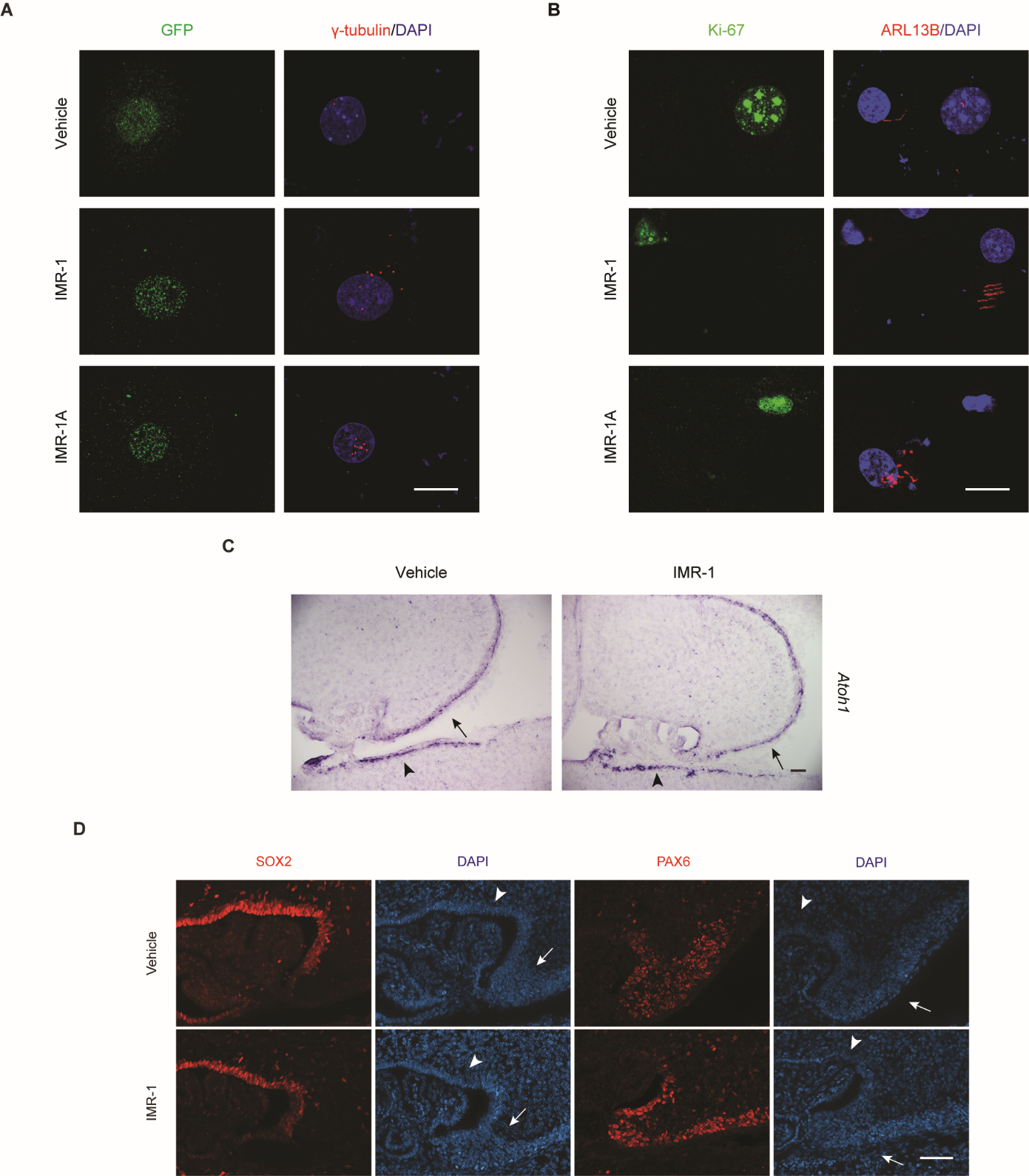


**Supplementary Figure S10.** **Analysis of gene expression in CP tumor cells and embryonic cerebellum after IMR-1 treatment. A, B** The expression of γ-tubulin (A, red), Ki-67 (B, green) and ARL13B (B, red) is shown in tumor cells treated with IMR-1/IMR-1A, or vehicle. GFP (B, green) labels tumor cells, DAPI staining (blue) labels nuclei. Scale bars, 20 µm. Results were obtained from five (A) and three (B) independent experiments, respectively. **C** Representative images of *in situ* hybridization of *Atoh1* in progenitors derived from upper rhombic lip (arrows) and lower rhombic lip (arrowheads) at day E17.5 is shown in wild type animals treated with vehicle or IMR-1 from day E10.5 to day E16.5. Scale bar, 50 µm. Images are representative of two independent experiments. **D** The expression of SOX2 (red) and PAX6 (red) in the ventricular zone (arrowheads) and upper rhombic lip (arrows) at day E17.5 is shown in wild type animals treated with vehicle or IMR-1 from day E10.5 to day E16.5. DAPI staining (blue) labels nuclei. Scale bar, 50 µm. Data represent two independent experiments.

**
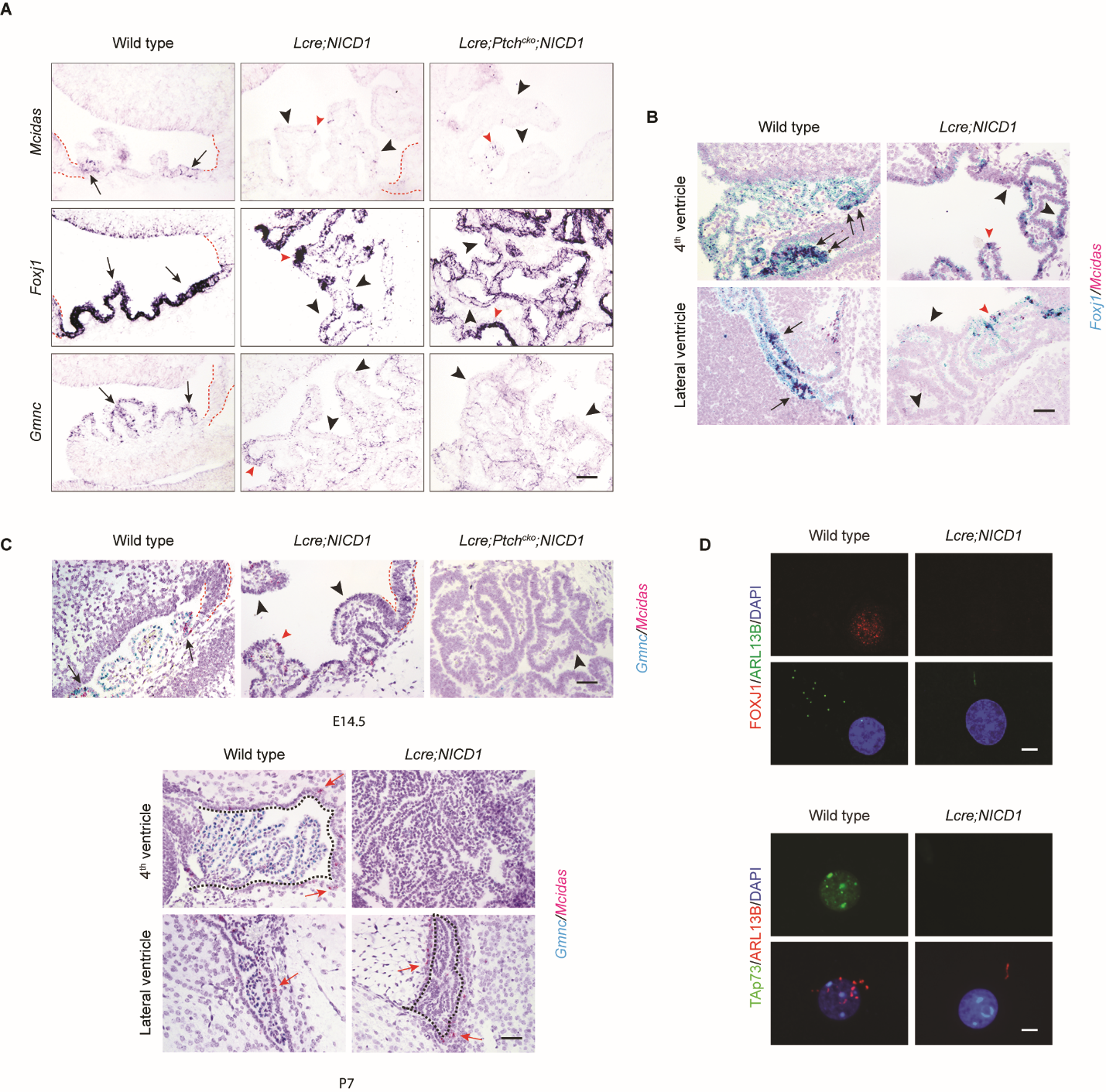
**

**Supplementary Figure S11.** **Repression of** ***Gmnc-Mcidas* signaling in NOTCH-driven CP tumor.** **A-C** Representative results of *in situ* hybridization of *Mcidas*, *Foxj1*, and *Gmnc* mRNAs (A), RNAscope study of *Foxj1* and *Mcidas* (B), or *Gmnc* and *Mcidas* (C), are shown at day E14.5 (A, and upper panel in C) in the upper roof plate (marked by dotted lines) and CP (arrows) of the hindbrain in wild type animals, CPP and abnormal CP growth (black arrowheads) in *Lcre;NICD1* and *Lcre;Ptch^cko^;NICD1* animals, respectively. *Gmnc^+^*, *Mcidas^+^*, or *Foxj1^+^* epithelial cells (red arrowheads) are mixed with tumor cells and abnormal cellular growth in *Lcre;NICD1* and *Lcre;Ptch^cko^;NICD1* animals, respectively. RNAscope analysis of the expression of *Gmnc* and *Mcidas* is shown at day P7 (lower panel in C) in CP in the 4^th^ and lateral ventricles in wild type animals, and CPP in *Lcre;NICD1* animals. *Mcidas*-positive ependymal cells (red arrows) in the walls lining the ventricles (marked by dotted lines) are shown. Scale bars, 50 µm. Results were obtained from at least three independent experiments. **D** The expression of FOXJ1 (upper panel, red), TAp73 (lower panel, green), and ARL13B is shown in cultured wild type CP epithelial cells and tumor cells from *Lcre;NICD1* animals. DAPI staining (blue) labels nuclei. Scale bars, 5 µm. Images are representative of at least three independent experiments.

**
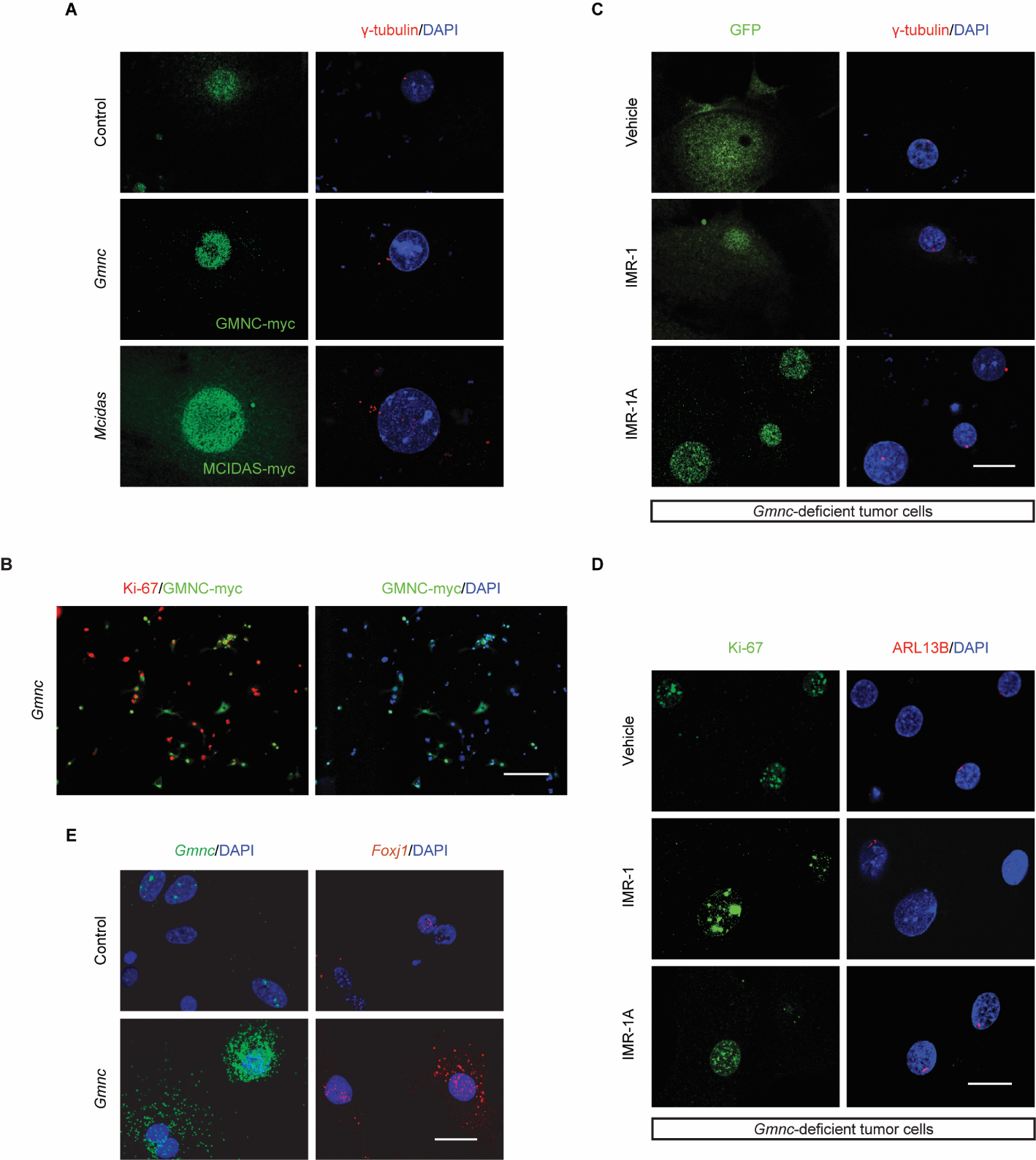
**

**Supplementary Figure S12.** ***Gmnc* suppression mediates multiciliation defects in NOTCH-driven CP tumor.** **A**, **B** The expression of γ-tubulin (A, red) and Ki-67 (B, red) is shown in tumor cells infected with viruses expressing GMNC-myc or MCIDAS-myc. GMNC-myc (green), MCIDAS-myc (green), or GFP (green) labels infected or control tumor cells, respectively. DAPI staining (blue) labels nuclei. Scale bars, 20 µm. Images are representative of at least three independent experiments. **C**, **D** The expression of γ-tubulin (C, red), Ki-67 (D, green) and ARL13B (D, red) is shown in *Gmnc*-deficient tumor cells treated with IMR-1/IMR-1A, or vehicle. GFP (C, green) labels tumor cells. DAPI staining (blue) labels nuclei. Scale bars, 20 µm. Data represent five (C) and three (D) independent experiments, respectively. **E** RNAscope analysis of *Gmnc* (green) and *Foxj1* (red) expression is shown in *Gmnc*-deficient tumor cells infected with viruses expressing GMNC-myc, or GFP only. DAPI staining (blue) labels nuclei. Scale bar, 20 µm. Results were obtained from three independent experiments.

***
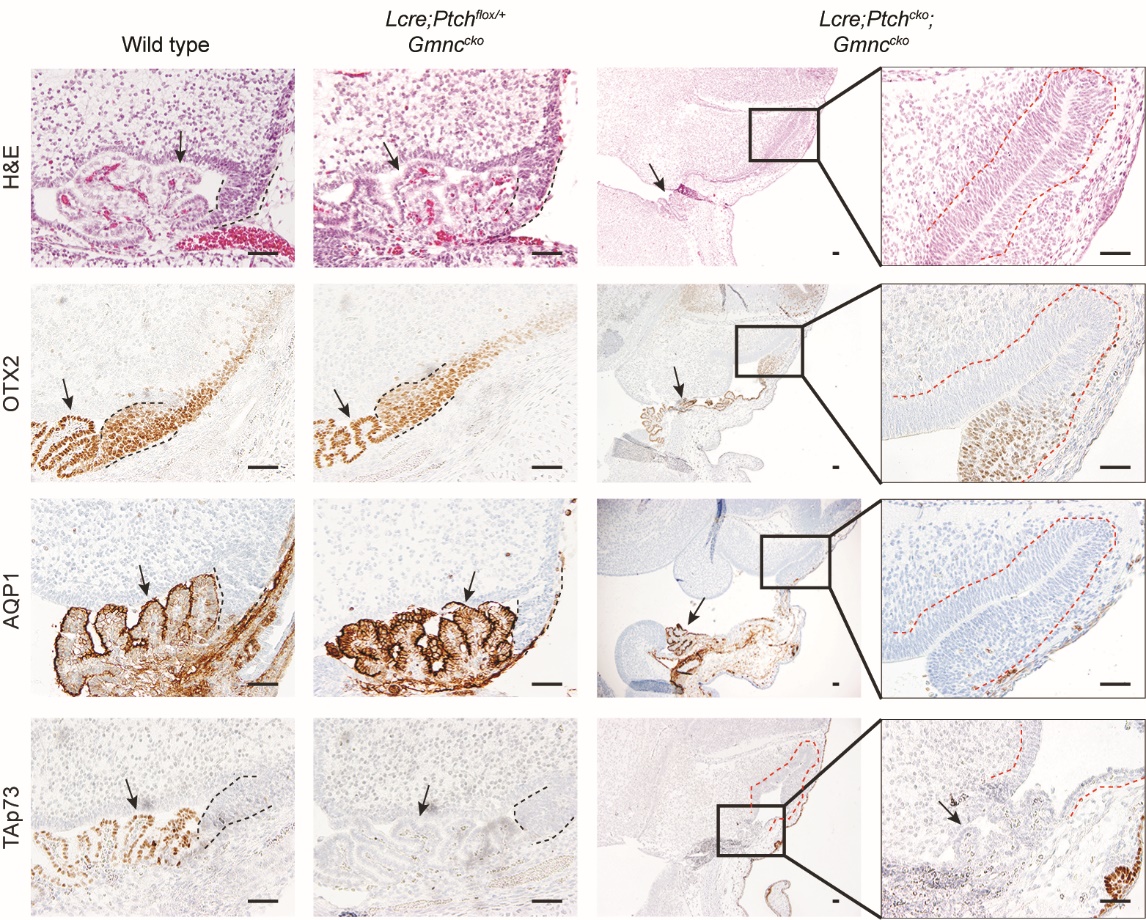
***

**Supplementary Figure S13.** **Aberrant SHH signaling and *Gmnc* loss fail to drive CP tumor in mice.** H&E staining and the expression of OTX2, AQP1 and TAp73 are shown of upper roof plate (marked by black dotted lines) and CP (arrows) in the hindbrain in wild type and *Lcre;Ptch^flox/+^;Gmnc^cko^* animals, and upper roof plate (marked by red dotted lines) and CP (arrows) in an *Lcre;Ptch^cko^;Gmnc^cko^* animal at day E14.5. Boxed regions are shown in higher magnification on the right. Notice the enlarged and folded roof plate in the midbrain-hindbrain region of *Lcre;Ptch^cko^;Gmnc^cko^* animals. Scale bars, 50 µm. Images are representative of at least three independent experiments.


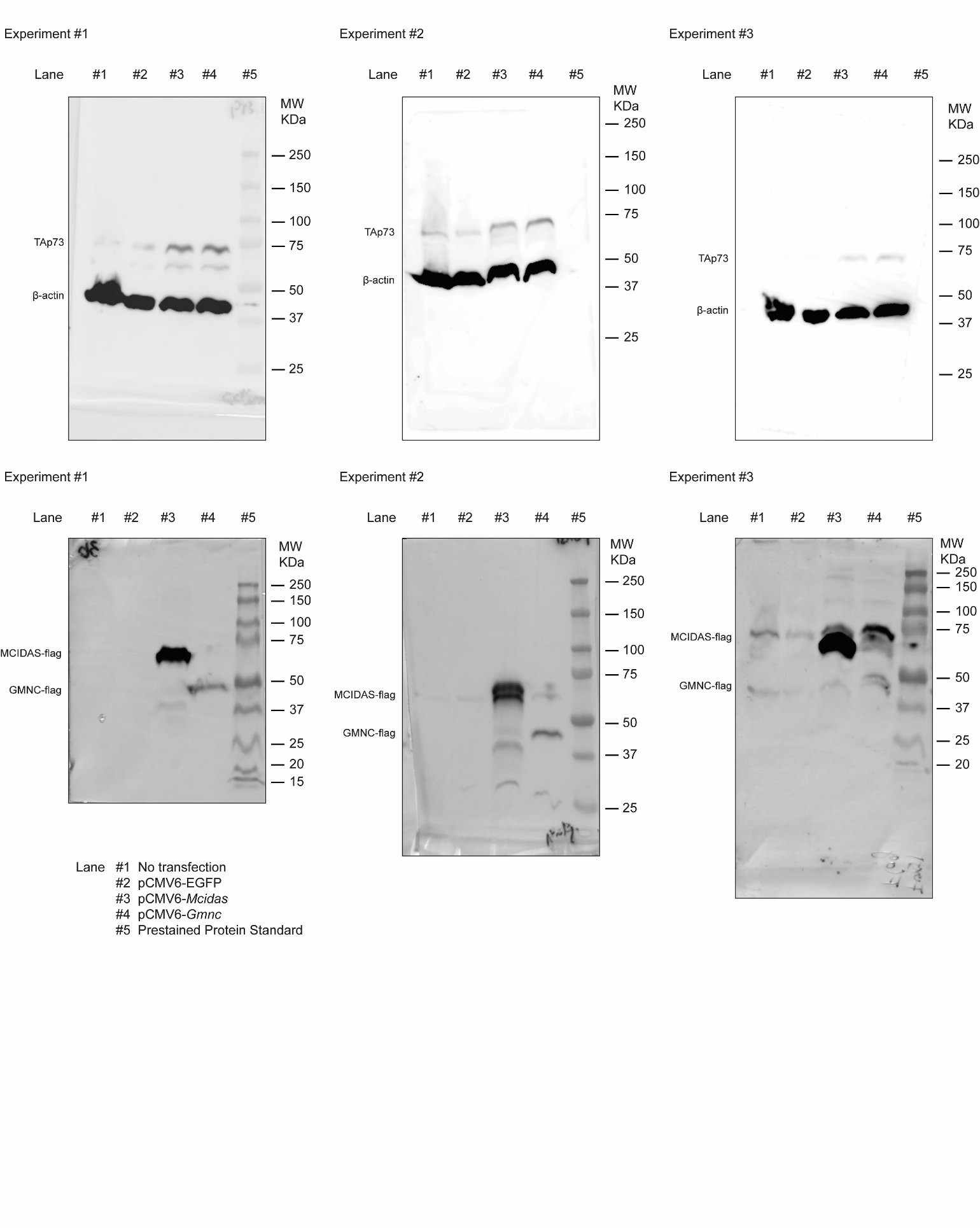


**Supplementary Figure S14.** **Analysis of GMNC-MCIDAS driven gene expression. A**  Immunoblot analysis of HEK293 cells transfected with plasmids expressing FLAG-tagged MCIDAS or GMNC, or GFP only. The expression of GMNC-FLAG, MCIDAS-FLAG, TAp73, and β-actin was shown in three independent experiments.
