# Supplementary material for: Disruption of GMNC-MCIDAS multiciliogenesis program is critical in choroid plexus carcinoma development
Source: Cell Death Differ. 2022 Mar 23;29(8):1596–610. doi: 10.1038/s41418-022-00950-z (PMC9345885; doi:10.1038/s41418-022-00950-z)
Supplement: Supplementary file 2 — Supplementary Table 1 [file 41418_2022_950_MOESM2_ESM.docx]

**Supplementary Table 1. Summary of phenotypic characteristics of mouse strains used for the study.**

| **Phenotype** | ***Lcre;Gmnc^flox/-^*** | ***Mcidas*^-/-^** | ***Lcre;p53^cko^;Rb^cko^* or** | ***Lcre;NICD1*** | ***Lcre;Ptch^cko^*** | ***Lcre;Ptch^cko^;NICD1*** | ***Lcre;Ptch^cko^;Gmnc^cko^*** |
| --- | --- | --- | --- | --- | --- | --- | --- |
|  | ***or Gmnc-/-*** |  | ***Lcre;p53^cko^;Rb^cko^;Gmnc^cko^*** |  |  |  |  |
| NOTCH pathway activation |  |  |  | + | - | + |  |
| *Hes1* expression in tumor |  |  |  | + | - | + |  |
| *Hes5* expression in tumor |  |  |  | + | - | + |  |
| SHH pathway activation |  |  |  | - | + | + |  |
| *Mycn* expression in tumor |  |  |  | + | - | + |  |
| *Gli1* expression in tumor |  |  |  | + | - | + |  |
| *Shh* expression in the CP |  |  |  | - | + | - |  |
| Cilia status in CP | monociliated | monociliated | monociliated & multiciliated |  | multiciliated |  | monociliated |
| Cilia status in tumor cells |  |  | monociliated | monociliated |  | monociliated |  |
| *Gmnc* | - | normal | - | - | normal | - |  |
| *Mcidas* | - |  | - | - | normal | - |  |
| TAp73 | - | normal | variable | - | normal | - | - |
| *Foxj1* | - | normal | - | - | normal | - |  |
| *Myb* | - |  |  |  |  |  |  |
| *Ccno* | - |  |  |  |  |  |  |
| Ki-67 expression in roof plate | normal |  |  | normal | + | + |  |
| Ki-67 expression in the CP | no |  | + | + | no | + |  |
| OTX2 expression in the CP | normal | normal | normal | normal | normal | normal | normal |
| AQP1 expression in the CP | + | normal | - | - | normal | - | normal |
| Cytokeratins expression in the CP | normal |  |  | - | normal | - |  |
| TTR expression in the CP | normal |  | - | - | normal | - |  |
| *Gdf7* expression in the CP |  |  | - |  |  |  |  |
