# Supplementary material for: Disruption of GMNC-MCIDAS multiciliogenesis program is critical in choroid plexus carcinoma development
Source: Cell Death Differ. 2022 Mar 23;29(8):1596–610. doi: 10.1038/s41418-022-00950-z (PMC9345885; doi:10.1038/s41418-022-00950-z)
Supplement: Supplementary file 3 — Supplementary Table 2 [file 41418_2022_950_MOESM3_ESM.docx]

**Supplementary Table 2. Sequence of primers and probes for RT-qPCR.**

| **Gene** | **Forward primer** | **Reverse primer** | **Probe** |
| --- | --- | --- | --- |
| mouse *Actb* | 5’-CAGCAAGCAGGAGTACGATGAG-3’ | 5’-CAGTAACAGTCCGCCTAGAAGCA-3’ | 5’-CCCTCCATCGTGCACCGCAA-3’ |
| mouse *Gmnc*  (exons 2/3) | 5’-ACCAGATTCTGACGTTGTAGTG-3’ | 5’-GAGGCTATGGAAGATGCTGT-3’ | 5'-AGGAGGCCAGAGTTATAATTGCCCG-3' |
| mouse *Gmnc*  (exons 4/5) | 5’-GAATCGCCCTCTGAGTTAAG-3’ | 5’-GTAGAGTATCCTGGAGCTGTTTATT-3’ | 5’-ATGTTCATGGGAAGAGGCTCAGCT-3’ |
| mouse *Gmnn* | 5’-TCTGCCAACAAGAGATCCATC-3’ | 5’-GGAGCCCAAGAGAATGTGAAG-3’ | 5'-TGTCCCAAGGAGAACGCTGAAGATG-3' |
| Mouse *TAp73* | 5’-AGCAATCTGACAGTACAACTTCT-3’ | 5’-CATCCCTTCCAATACCGACTAC-3’ | 5'-TCACCTTCCAGCAGTCGAGCAC-3' |
| mouse *Aqp1* | 5’-CACCTCCTCCCTAGTCGACAATT-3’ | 5’-TACCAGCTGCAGAGTGCCAAT-3’ | 5’-CTTGGCCGCAATGACCTGGCTCAC-3’ |
| mouse *Ttr* | 5’-GACAGGATGGCTTCCCTTCG-3’ | 5’-CCAGGACTTTGACCATCAGAGG-3’ | 5’-CCTCCTTTGCCTCGCTGGACTGGT-3’ |
| Human *GAPDH* | 5’-GGGTGTGAACCATGAGAAGTAT-3’ | 5’-CTAAGCAGTTGGTGGTGCAGG-3’ | 5'-CAAGATCATCAGCAATGC-3' |
|  | | | |
