# Supplementary material for: Disruption of GMNC-MCIDAS multiciliogenesis program is critical in choroid plexus carcinoma development
Source: Cell Death Differ. 2022 Mar 23;29(8):1596–610. doi: 10.1038/s41418-022-00950-z (PMC9345885; doi:10.1038/s41418-022-00950-z)
Supplement: Supplementary file 4 — Original Data File - uncropped images of western blots [file 41418_2022_950_MOESM4_ESM.docx]

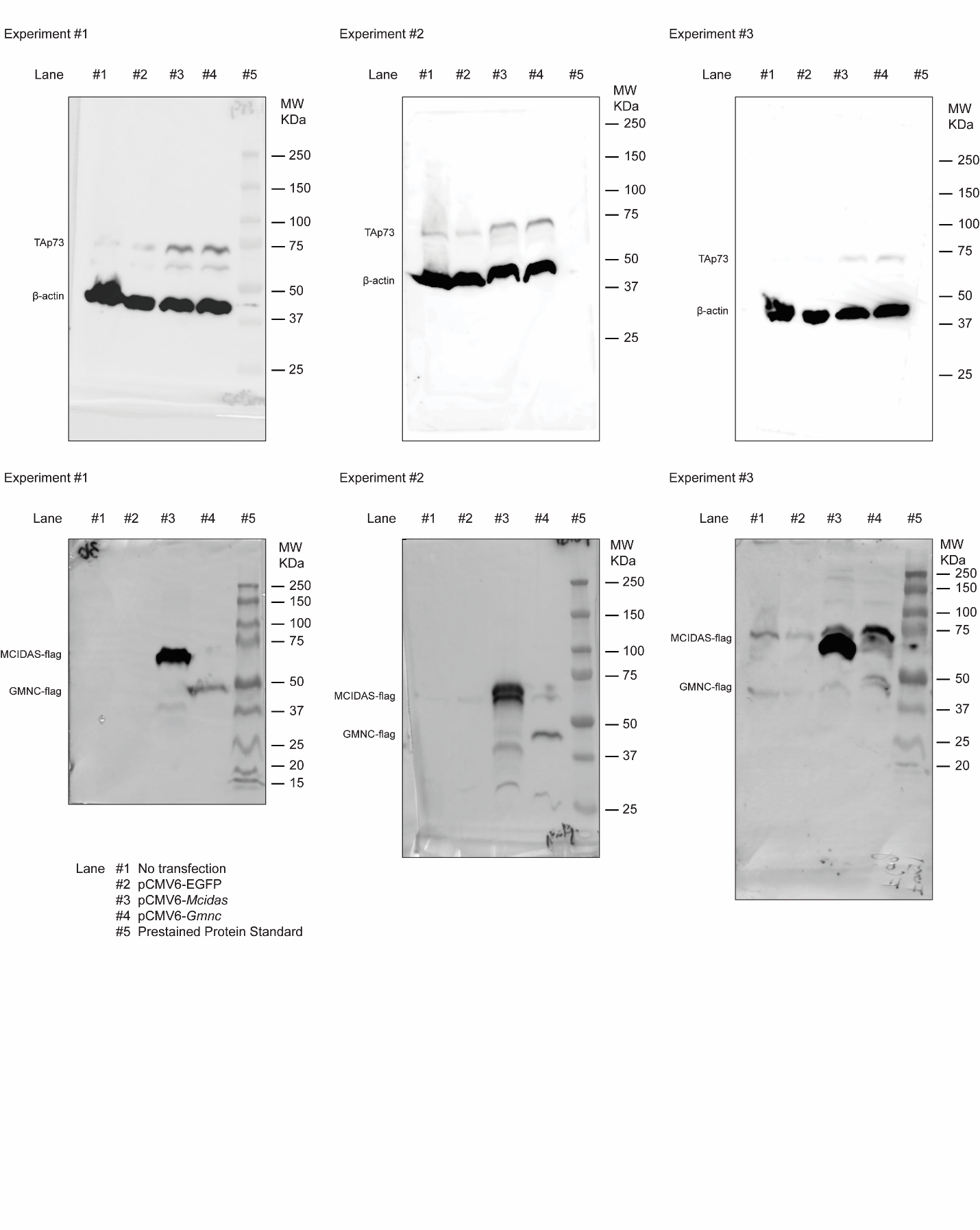


**Supplementary Figure S14.** **Analysis of GMNC-MCIDAS driven gene expression. A**  Immunoblot analysis of HEK293 cells transfected with plasmids expressing FLAG-tagged MCIDAS or GMNC, or GFP only. The expression of GMNC-FLAG, MCIDAS-FLAG, TAp73, and β-actin was shown in three independent experiments.
