# Supplementary material for: Disruption of GMNC-MCIDAS multiciliogenesis program is critical in choroid plexus carcinoma development
Source: Cell Death Differ. 2022 Mar 23;29(8):1596–610. doi: 10.1038/s41418-022-00950-z (PMC9345885; doi:10.1038/s41418-022-00950-z)
Supplement: Supplementary file 5 — Signed - Pre-Authorship form [file 41418_2022_950_MOESM5_ESM.pdf]

SPRINGER NATURE Change of authorship request form - Journals (pre-acceptance)

Important information. Please read.

- This form should be used by authors to request any change in authorship (adding/deleting authors) including changes in corresponding authors. This form should not be used for name changes. Please fully complete all sections. Use black ink and block capitals and provide each author's full name with the given name first followed by the family name.

- By signing this declaration, all authors guarantee that the order of the authors are in accordance with their scientific contribution, if applicable as different conventions apply per discipline, and that only authors have been added who made a meaningful contribution to the work.

- Please note, in author collaborations where there is formal agreement for representing the collaboration, it is sufficient for the representative or legal guarantor (usually the corresponding author) to complete and sign the Authorship Change Form on behalf of all authors, **next to the added/removed author(s)**. **(Complete Section 3, followed by Section 6.)**
  In author collaborations where there is no formal agreement for representing the collaboration and **there are more than 10 authors**, one may sign for all, provided the signer appends correspondence that attests that each of the authors have agreed to the change **and the added/removed authors sign the form**. **(Complete Section 3, followed by Section 6.)**

- Please note, we cannot investigate or mediate any authorship disputes. If you are unable to obtain agreement from all authors (including those who you wish to be removed) you must refer the matter to your institution(s) for investigation. Please inform us if you need to do this.

- If you are not able to return a fully completed form within **30 days** of the date that it was sent to the author requesting the change, we may have to withdraw your manuscript. We cannot publish manuscripts where authorship has not been agreed by all authors (including those who have been removed).

- Incomplete forms will be rejected.

- Please return/upload this form, fully completed, to the Journals Editorial Office. The Journal and/or Publisher will consider the information you have provided to decide whether to approve the proposed change in authorship. We may decide to contact your institution for more information or undertake a further investigation, if appropriate, before making a final decision.

SPRINGER NATURE Springer Nature is one of the world's leading global research, educational and professional publishers, created in May 2015 through the combination of Nature Publishing Group, Palgrave Macmillan, Macmillan Education and Springer Science+Business Media.

SPRINGER NATURE

Change of authorship request form - Journals (pre-acceptance)

Section 1: Please provide the current title of manuscript

Manuscript ID no.: CDD-21-0809RR

Title: Disruption of GMNC-MCIDAS multiciliogenesis program is critical in choroid plexus carcinoma development

Section 2: Please provide the previous authorship, in the order shown on the manuscript before the changes were introduced. Please indicate the corresponding author by adding (CA) behind the name.

	First name(s)	Family name	ORCID or SCOPUS id, if available
1st author	Qun	Li	
2nd author	Zhiyuan	Han	
3rd author	Navleen	Singh	
4th author	Berta	Terré	0000-0002-0382-6129
5th author	Ryann	Fame	0000-0002-8244-2624
6th author	Uzayr	Arif	
7th author	Thomas	Page	
8th author	Tasneem	Zahran	
9th author	Ahmed	Abdeltawab	
10th author	Yuan	Huang	

Please use an additional sheet if there are more than 10 authors.

SPRINGER NATURE Springer Nature is one of the world’s leading global research, educational and professional publishers, created in May 2015 through the combination of Nature Publishing Group, Palgrave Macmillan, Macmillan Education and Springer Science+Business Media.

SPRINGER NATURE

Change of authorship request form - Journals (pre-acceptance)

Section 1: Please provide the current title of manuscript

Manuscript ID no.: CDD-21-0809RR

Title: Disruption of GMNC-MCIDAS multiciliogenesis program is critical in choroid plexus carcinoma development

Section 2: Please provide the previous authorship, in the order shown on the manuscript before the changes were introduced. Please indicate the corresponding author by adding (CA) behind the name.

	First name(s)	Family name	ORCID or SCOPUS id, if available
1st author	Ping	Cao	
2nd author	Jun	Wang	
3rd author	Hao	Lu	
4th author	Hart	Lidov	
5th author	Kameswaran	Surendran	
6th author	Lizhao	Wu	
7th author	Ulrich	Schüller	
8th author	Robert	Wechsler-Reya	
9th author	Maria	Lehtinen	
10th author	Sudipto	Roy	

Please use an additional sheet if there are more than 10 authors.

SPRINGER NATURE Springer Nature is one of the world's leading global research, educational and professional publishers, created in May 2015 through the combination of Nature Publishing Group, Palgrave Macmillan, Macmillan Education and Springer Science+Business Media.

SPRINGER NATURE

Change of authorship request form - Journals (pre-acceptance)

Section 1: Please provide the current title of manuscript

Manuscript ID no.: CDD-21-0809RR

Title: Disruption of GMNC-MCIDAS multiciliogenesis program is critical in choroid plexus carcinoma development

Section 2: Please provide the previous authorship, in the order shown on the manuscript before the changes were introduced. Please indicate the corresponding author by adding (CA) behind the name.

	First name(s)	Family name	ORCID or SCOPUS id, if available
1st author	Zhongmin	Liu, (CA)	0000-0003-4709-4027
2nd author	Travis	Stracker	0000-0002-8650-2081
3rd author	Haotian	Zhao, (CA)	0000-0003-2315-8452
4th author			
5th author			
6th author			
7th author			
8th author			
9th author			
10th author			

Please use an additional sheet if there are more than 10 authors.

SPRINGER NATURE Springer Nature is one of the world's leading global research, educational and professional publishers, created in May 2015 through the combination of Nature Publishing Group, Palgrave Macmillan, Macmillan Education and Springer Science+Business Media.

SPRINGER NATURE

Change of authorship request form - Journals (pre-acceptance)

Section 3: Please provide a justification for change. Please use this section to explain your reasons for changing the authorship of your manuscript, e.g. what necessitated the change in authorship? Please refer to the (journal) policy pages for more information about authorship. Please explain why omitted authors were not originally included and/or why authors were removed on the submitted manuscript.

During the revision process, Mr. James Virga and Dr. Ying-Tao Zhao have contributed to the study through acquisition and analysis of data. They were added as co-authors in the revised manuscript. Dr. Stracker made major contribution to the revisions and was added as co-corresponding author.

Section 4: Proposed new authorship. Please provide your new authorship list in the order you would like it to appear on the manuscript. Please indicate the corresponding author by adding (CA) behind the name. <u>If the Corresponding Author has changed, please indicate the reason under section 3</u>.

	First name(s)	Family name (this name will appear in full on the final publication and will be searchable in various abstract and indexing databases)	Affiliated institute	E-mail address
1st author	Qun	Li	Shanghai East Hospital	liqun@fudan.edu.cn
2nd author	Zhiyuan	Han	New York Institute of Technology	zac_zhiyuan.han@hotmail.com
3rd author	Navleen	Singh	New York Institute of Technology	nsingh36@nyit.edu
4th author	Berta	Terré	Institute for Research in Biomedicine	berta.terre@crick.ac.uk
5th author	Ryann	Fame	Boston Children's Hospital	Ryan.Fame@childrens.harvard.edu
6th author	Uzayr	Arif	New York Institute of Technology	uzrif@nyit.edu
7th author	Thomas	Page	New York Institute of Technology	tpage01@nyit.edu
8th author	Tasneem	Zahran	New York Institute of Technology	tzahran@nyit.edu
9th author	Ahmed	Abdeltawab	New York Institute of Technology	aabdel20@nyit.edu
10th author	Yuan	Huang	New York Institute of Technology	yhuang45@nyit.edu

Please use an additional sheet if there are more than 10 authors.

SPRINGER NATURE Springer Nature is one of the world's leading global research, educational and professional publishers, created in May 2015 through the combination of Nature Publishing Group, Palgrave Macmillan, Macmillan Education and Springer Science+Business Media.

SPRINGER NATURE

Change of authorship request form - Journals (pre-acceptance)

Section 3: Please provide a justification for change. Please use this section to explain your reasons for changing the authorship of your manuscript, e.g. what necessitated the change in authorship? Please refer to the (journal) policy pages for more information about authorship. Please explain why omitted authors were not originally included and/or why authors were removed on the submitted manuscript.

During the revision process, Mr. James Virga and Dr. Ying-Tao Zhao have contributed to the study through acquisition and analysis of data. They were added as co-authors in the revised manuscript. Dr. Stracker made major contribution to the revisions and was added as co-corresponding author.

Section 4: Proposed new authorship. Please provide your new authorship list in the order you would like it to appear on the manuscript. Please indicate the corresponding author by adding (CA) behind the name. If the Corresponding Author has changed, please indicate the reason under section 3.

	First name(s)	Family name (this name will appear in full on the final publication and will be searchable in various abstract and indexing databases)	Affiliated institute	E-mail address
1st author	Ping	Cao	New York Institute of Technology	pcao06@nyit.edu
2nd author	Jun	Wang	Sanford Burnham Prebys Medical Institute	junw@sbpdiscovery.org
3rd author	Hao	Lu	Institute of Molecular and Cell Biology	luhao@imcb.a-star.edu.sg
4th author	Hart	Lidov	Boston Children's Hospital	Hart.Lidov@childrens.harvard.edu
5th author	Kameswaran	Surendran	Sanford Research	kameswaran.surendran@sanfordhealth.org
6th author	Lizhao	Wu	China Medical University	lizhaowu@mail.cmu.edu.cn
7th author	James	Virga	New York Institute of Technology	jvirga01@nyit.edu
8th author	Ying-Tao	Zhao	New York Institute of Technology	yzhao47@nyit.edu
9th author	Ulrich	Schüller	University Medical Center Hamburg-Eppendorf	u.schueller@uke.de
10th author	Robert	Wechsler-Reya	Sanford Burnham Prebys Medical Discovery Institute	rwreya@SBPdiscovery.org

Please use an additional sheet if there are more than 10 authors.

SPRINGER NATURE Springer Nature is one of the world's leading global research, educational and professional publishers, created in May 2015 through the combination of Nature Publishing Group, Palgrave Macmillan, Macmillan Education and Springer Science+Business Media.

Change of authorship request form - Journals (pre-acceptance)

Section 3: Please provide a justification for change. Please use this section to explain your reasons for changing the authorship of your manuscript, e.g. what necessitated the change in authorship? Please refer to the (journal) policy pages for more information about authorship. Please explain why omitted authors were not originally included and/or why authors were removed on the submitted manuscript.

During the revision process, Mr. James Virga and Dr. Ying-Tao Zhao have contributed to the study through acquisition and analysis of data. They were added as co-authors in the revised manuscript. Dr. Stracker made major contribution to the revisions and was added as co-corresponding author.

Section 4: Proposed new authorship. Please provide your new authorship list in the order you would like it to appear on the manuscript. Please indicate the corresponding author by adding (CA) behind the name. If the Corresponding Author has changed, please indicate the reason under section 3.

	First name(s)	Family name (this name will appear in full on the final publication and will be searchable in various abstract and indexing databases)	Affiliated institute	E-mail address
1st author	Maria	Lehtinen	Boston Children's Hospital	Maria.Lehtinen@childrens.harvard.edu
2nd author	Sudipto	Roy	Institute of Molecular and Cell Biology	sudiptor@imcb.a-star.edu.sg
3rd author	Zhongmin	Liu, (CA)	Shanghai East Hospital	liu.zhongmin@tongji.edu.cn
4th author	Travis	Stracker, (CA)	Institute for Research in Biomedicine	travis.stracker@nih.gov
5th author	Haotian	Zhao, (CA)	New York Institute of Technology	hzhao10@nyit.edu
6th author				
7th author				
8th author				
9th author				
10th author				

Please use an additional sheet if there are more than 10 authors.

SPRINGER NATURE Springer Nature is one of the world's leading global research, educational and professional publishers, created in May 2015 through the combination of Nature Publishing Group, Palgrave Macmillan, Macmillan Education and Springer Science+Business Media.

SPRINGER NATURE

Change of authorship request form - Journals (pre-acceptance)

Section 5: Author contribution, Acknowledgement and Disclosures. Please use this section to provide a new disclosure statement and, if appropriate, acknowledge any contributors who have been removed as authors and ensure you state what contribution any new authors made (if applicable per the journal or book (series) policy). Please ensure these are updated in your manuscript - after approval of the change(s) - as our production department will not transfer the information in this form to your manuscript.

New acknowledgements:

We are grateful to Drs. Roger Packer, Huizhen Zhang, Brian Rood, William Weiss, Joanna Philips, Michael Taylor, James Loukides, and Sandro Santagata for providing tissue samples, and Monica Calicchio for assisting with tissue specimens from Boston Children's Hospital (BCH). We thank Lars Udo-Bellner, Melanie Schweitzer, Amanda Chiang, Tamanna Sarowar, Brightlyn Kwa, Maheen Umer, Mariam Zahran, Navjot Guru, and Claire Evans for technical assistance.

New Disclosures (financial and non-financial interests, funding):

The authors declare that they have no competing financial interest.

New Author Contributions statement (if applicable per the journal policy):

Q.L., Z.H., Z.L., and H.Z. conceived and designed the study; S.R., Z.L., T.H.S., and H.Z. performed development of methodology and writing, review, editing, and revision of the manuscript; Q.L., Z.H., N.S., B.T., R.M.F., M.K.L., H.G.W.L., U.A., T.D.P., J.Q.V., and Y.Z. provided acquisition, analysis and interpretation of data, and statistical analysis; T.Z., A.A., Y.H., P.C., H.L., S.R., J.W., R.J.W., K.S., L.W., U.S., and Z.L. provided technical and material support. All authors read and approved the final paper.

State 'Not applicable' if there are no new authors.

SPRINGER NATURE Springer Nature is one of the world's leading global research, educational and professional publishers, created in May 2015 through the combination of Nature Publishing Group, Palgrave Macmillan, Macmillan Education and Springer Science+Business Media.

SPRINGER NATURE

Change of authorship request form - Journals (pre-acceptance)

Section 6: Declaration of agreement. *All* authors, unchanged, new and removed *must* sign this declaration.
(NB: Please print the form, (docu)-sign and return/upload a scanned copy. Please note that signatures that have been inserted as an image file are acceptable as long as it is handwritten. Typed names in the signature box are unacceptable.) *** Please delete as appropriate. Delete all of the bold if you were on the original authorship list and are remaining as an author.**

	First name	Family name		Signature	Date
1st author	Qun	Li	I agree to the proposed new authorship shown in section 4 and the proposed change in corresponding author	[illegible]	01/20/22
2nd author	Zhiyuan	Han	I agree to the proposed new authorship shown in section 4 and the proposed change in corresponding author	[illegible]	21/1/22
3rd author	Navleen	Singh	I agree to the proposed new authorship shown in section 4 and the proposed change in corresponding author	[illegible]	1/24/22
4th authors	Berta	Terré	I agree to the proposed new authorship shown in section 4 and the proposed change in corresponding author	[illegible]	01/26/22
5th author	Ryann	Fame	I agree to the proposed new authorship shown in section 4 and the proposed change in corresponding author	[illegible]	01/26/22
6th author	Uzayr	Arif	I agree to the proposed new authorship shown in section 4 and the proposed change in corresponding author	[illegible]	01/[illegible]/22
7th author	Thomas	Page	I agree to the proposed new authorship shown in section 4 and the proposed change in corresponding author	[illegible]	1/21/22

SPRINGER NATURE Springer Nature is one of the world's leading global research, educational and professional publishers, created in May 2015 through the combination of Nature Publishing Group, Palgrave Macmillan, Macmillan Education and Springer Science+Business Media.

SPRINGER NATURE Change of authorship request form - Journals (pre-acceptance)

	First name	Family name		Signature	Date
8th author	Tasneem	Zahran	I agree to the proposed new authorship shown in section 4 and the proposed change in corresponding author	Tasneem Zahran	01/21/2022
9th author	Ahmed	Abdeltawab	I agree to the proposed new authorship shown in section 4 and the proposed change in corresponding author	[illegible]	01/26/22
10th author	Yuan	Huang	I agree to the proposed new authorship shown in section 4 and the proposed change in corresponding author	[illegible]	01/21/2022

Please use an additional sheet if there are more than 10 authors.

In case of author collaborations with formal agreement:

	Name of consortium/consortia	First name	Family name		Signature	Date
Representative/legal guarantor				I agree to the proposed new authorship shown in section 4 **/and the addition/removal*of my name to the authorship list** /and the proposed change in corresponding author		

Both added/removed authors should complete the information in the first table under Section 6.

---- End of form ----

SPRINGER NATURE Springer Nature is one of the world's leading global research, educational and professional publishers, created in May 2015 through the combination of Nature Publishing Group, Palgrave Macmillan, Macmillan Education and Springer Science+Business Media.

SPRINGER NATURE

Change of authorship request form - Journals (pre-acceptance)

Section 6: Declaration of agreement. *All* authors, unchanged, new and removed *must* sign this declaration.

(NB: Please print the form, (docu)-sign and return/upload a scanned copy. Please note that signatures that have been inserted as an image file are acceptable as long as it is handwritten. Typed names in the signature box are unacceptable.) *** Please delete as appropriate. Delete all of the bold if you were on the original authorship list and are remaining as an author.**

	First name	Family name		Signature	Date
1st author	Ping	Cao	I agree to the proposed new authorship shown in section 4 and the proposed change in corresponding author	[illegible]	01/21/2022
2nd author	Jun	Wang	I agree to the proposed new authorship shown in section 4 and the proposed change in corresponding author	[illegible]	01/26/22
3rd author	Hao	Lu	I agree to the proposed new authorship shown in section 4 and the proposed change in corresponding author	[illegible]	01/26/22
4th authors	Hart	Lidov	I agree to the proposed new authorship shown in section 4 and the proposed change in corresponding author	[illegible]	01/26/22
5th author	Kameswaran	Surendran	I agree to the proposed new authorship shown in section 4 and the proposed change in corresponding author	[illegible]	01/26/22
6th author	Lizhao	Wu	I agree to the proposed new authorship shown in section 4 and the proposed change in corresponding author	[illegible]	01/26/22
7th author	James	Virga	I agree to the proposed new authorship shown in section 4 **/and the addition of my name to the authorship list** /and the proposed change in corresponding author	James Virga	01/21/2022

SPRINGER NATURE Springer Nature is one of the world's leading global research, educational and professional publishers, created in May 2015 through the combination of Nature Publishing Group, Palgrave Macmillan, Macmillan Education and Springer Science+Business Media.

SPRINGER NATURE

Change of authorship request form - Journals (pre-acceptance)

	First name	Family name		Signature	Date
8th author	Ying-Tao	Zhao	I agree to the proposed new authorship shown in section 4 **/and the addition of my name to the authorship list** /and the proposed change in corresponding author		1/21/2022
9th author	Ulrich	Schüller	I agree to the proposed new authorship shown in section 4 and the proposed change in corresponding author		01/26/22
10th author	Robert	Wechsler-Reya	I agree to the proposed new authorship shown in section 4 and the proposed change in corresponding author		01/26/2022

Please use an additional sheet if there are more than 10 authors.

In case of author collaborations with formal agreement:

	Name of consortium/consortia	First name	Family name		Signature	Date
Representative/legal guarantor				I agree to the proposed new authorship shown in section 4 **/and the addition/removal*of my name to the authorship list** /and the proposed change in corresponding author		

Both added/removed authors should complete the information in the first table under Section 6.

---- End of form ----

SPRINGER NATURE Springer Nature is one of the world's leading global research, educational and professional publishers, created in May 2015 through the combination of Nature Publishing Group, Palgrave Macmillan, Macmillan Education and Springer Science+Business Media.

SPRINGER NATURE

Change of authorship request form - Journals (pre-acceptance)

Section 6: Declaration of agreement. *All* authors, unchanged, new and removed *must* sign this declaration.
(NB: Please print the form, (docu)-sign and return/upload a scanned copy. Please note that signatures that have been inserted as an image file are acceptable as long as it is handwritten. Typed names in the signature box are unacceptable.) *** Please delete as appropriate. Delete all of the bold if you were on the original authorship list and are remaining as an author.**

	First name	Family name		Signature	Date
1st author	Maria	Lehtinen	I agree to the proposed new authorship shown in section 4 and the proposed change in corresponding author	[illegible]	01/26/22
2nd author	Sudipto	Roy	I agree to the proposed new authorship shown in section 4 and the proposed change in corresponding author	[illegible]	01/26/22
3rd author	Zhongmin	Liu	I agree to the proposed new authorship shown in section 4 and the proposed change in corresponding author	[illegible]	25/1/2022
4th authors	Travis	Stracker	I agree to the proposed new authorship shown in section 4 and the proposed change in corresponding author	[illegible]	01/26/22
5th author	Haotian	Zhao	I agree to the proposed new authorship shown in section 4 and the proposed change in corresponding author	[illegible]	01/26/22
6th author			I agree to the proposed new authorship shown in section 4 **/and the addition/removal*of my name to the authorship list** /and the proposed change in corresponding author		
7th author			I agree to the proposed new authorship shown in section 4 **/and the addition/removal*of my name to the authorship list** /and the proposed change in corresponding author		

SPRINGER NATURE Springer Nature is one of the world's leading global research, educational and professional publishers, created in May 2015 through the combination of Nature Publishing Group, Palgrave Macmillan, Macmillan Education and Springer Science+Business Media.

**From:**	Berta Terre Torras
**To:**	Haotian Zhao
**Subject:**	Re: Manuscript
**Date:**	Monday, January 24, 2022 5:42:12 AM

Sure, I am happy to approve the changes. However, I don't know how to do it. Could you please guide me how to do it?

Thanks

From: Haotian Zhao <hzhao10@nyit.edu>
Date: Friday, 21 January 2022 at 20:27
To: Berta Terre Torras <berta.terre@crick.ac.uk>
Subject: RE: Manuscript

External Sender: Use caution.

Hi Berta,

Thanks for confirming. During revision, Mr. James Virga and Dr. Yingtao Zhao have contributed to the study through acquisition and analysis data. They were added as co-authors in the revised manuscript. Dr. Stracker made major contribution to the revisions and was added as co-corresponding author. Can you please approve of these changes? Thanks.

Haotian.

From: Berta Terre Torras <berta.terre@crick.ac.uk>
Sent: Friday, January 21, 2022 4:33 AM
To: Haotian Zhao <hzhao10@nyit.edu>; Stracker, Travis (NIH/NCI) [E] <travis.stracker@nih.gov>
Subject: Re: Manuscript

Caution: This message originated from an **External Source**. Use proper judgment when opening attachments, clicking links or responding to this email.

Dear Haotian,

These are really good news, good job team!

An update of my information (I am the one working at the Crick):

Institute for Research in Biomedicine (IRB Barcelona), The Barcelona Institute of Science and Technology, C/ Baldiri Reixac 10, Barcelona 08028, Spain.

Present address: The Francis Crick Institute, 1 Midland Road, London NW1 1AT, UK

Kind regards,

B

From: Haotian Zhao <hzhao10@nyit.edu>
Date: Friday, 21 January 2022 at 01:33
To: Stracker, Travis (NIH/NCI) [E] <travis.stracker@nih.gov>
Cc: Berta Terre Torras <berta.terre@crick.ac.uk>
Subject: Manuscript

External Sender: Use caution.

Dear Travis and Berta,

I hope everything is well with you. It took a long time in the making, but I am glad to tell you that our manuscript "Disruption of GMNC-MCIDAS multiciliogenesis program is critical in choroid plexus carcinoma development" has been provisionally accepted for publication in Cell Death and Differentiation. I am currently finalizing the manuscript for acceptance and production. Here is what I have for your and Berta's information:

Institute for Research in Biomedicine (IRB Barcelona), The Barcelona Institute of Science and Technology, C/ Baldiri Reixac 10, Barcelona 08028, Spain.

The National Cancer Institute, Center for Cancer Research, Radiation Oncology Branch, Bethesda MD, 20892, USA.

Present address: The Francis Crick Institute, 1 Midland Road, London NW1 1AT, UK

T.H.S. was supported by the Spanish Ministry of Science, Innovation and Universities (MCIU: PGC2018-095616-B-I00/GINDATA and FEDER), the Centres of Excellence Severo Ochoa award and the CERCA Programme and is currently supported by the NCI Intramural program.

Please let me know if you would like to include your other information. In addition, during the revision process, Mr. James Virga and Dr. Yingtao Zhao have contributed to the study through acquisition and analysis data. They were added as co-authors in the revised manuscript. Dr. Stracker made major contribution to the revisions and was added as co-corresponding author. Please approve of these changes so I could update the author list. Thanks.

Haotian.

The Francis Crick Institute Limited is a registered charity in England and Wales no. 1140062 and a company registered in England and Wales no. 06885462, with its registered office at 1 Midland Road London NW1 1AT

The Francis Crick Institute Limited is a registered charity in England and Wales no. 1140062 and a company registered in England and Wales no. 06885462, with its registered office at 1 Midland Road London NW1 1AT

Haotian Zhao

**From:**	Fame, Ryan <Ryan.Fame@childrens.harvard.edu>
**Sent:**	Thursday, January 20, 2022 8:08 PM
**To:**	Haotian Zhao
**Cc:**	Lidov, Hart; Lehtinen, Maria
**Subject:**	Re: Manuscript [EXTERNAL]

Caution: This message originated from an **External Source**. Use proper judgment when opening attachments, clicking links or responding to this email.

Dear Haiotian~

Congratulations on the acceptance to publish this important work! It has been a pleasure working with your team and I thank you for including me in this investigation. I would love to read the accepted version when it is convenient to send.

The affiliation and support look correct to me and I approve the author changes you have included. You likely already have it, but if you need my ORCID it is: 0000-0002-8244-2624

Sincerely
Ryann

From: Haotian Zhao <hzhao10@nyit.edu>
Sent: Thursday, January 20, 2022 8:00 PM
To: Lehtinen, Maria
Cc: Fame, Ryan; Lidov, Hart
Subject: Manuscript [EXTERNAL]

*** External Email - Caution ***

Dear Maria, Ryann and Hart,

I hope everything is well with you. It took a long time in the making, but I am very glad to tell you that our manuscript "Disruption of GMNC-MCIDAS multiciliogenesis program is critical in choroid plexus carcinoma development" has been provisionally accepted for publication in Cell Death and Differentiation. I am currently finalizing the manuscript for acceptance and production. Here is what I have for your information:

Department of Pathology, Boston Children's Hospital, Boston, Massachusetts 02115, USA.

This project was supported by NIH T32 HL110852 and BCH Faculty Development Fellowship (R.M.F.); NIH R01 NS088566 (MKL) and the New York Stem Cell Foundation (M.K.L.). M.K. Lehtinen is a New York Stem Cell Foundation – Robertson Investigator.

Please let me know if you would like to include your other information. In addition, during the revision process, Mr. James Virga and Dr. Yingtao Zhao have contributed to the study through acquisition and analysis data. They were added

as co-authors in the revised manuscript. Dr. Stracker made major contribution to the revisions and was added as co-corresponding author. Please approve of these changes so I could update the author list. Thanks.

Haotian.

Haotian Zhao

From: Uzayr Arif <uarif@nyit.edu>
Sent: Friday, January 21, 2022 6:05 PM
To: Haotian Zhao
Subject: Re: Uzayr, accepting co-authorship

I approve these changes
-Uzayr Arif

On Fri, Jan 21, 2022 at 2:53 PM Haotian Zhao <hzhao10@nyit.edu> wrote:

> Hi Uzayr,
>
> I hope everything is well with you. It took a long time in the making, but I am glad to tell you that our manuscript "Disruption of GMNC-MCIDAS multiciliogenesis program is critical in choroid plexus carcinoma development" has been provisionally accepted for publication in Cell Death and Differentiation. I am currently finalizing the manuscript for official acceptance.
>
> During the revision process, Mr. James Virga and Dr. Yingtao Zhao have contributed to the study through acquisition and analysis data. They were added as co-authors in the revised manuscript. Dr. Stracker made major contribution to the revisions and was added as co-corresponding author. As a co-author, can you please approve of these changes in authors. Thanks.
>
> Haotian.
>
> **From:** Uzayr Arif <uarif@nyit.edu>
> **Sent:** Friday, January 21, 2022 1:10 PM
> **To:** Haotian Zhao <hzhao10@nyit.edu>
> **Subject:** Uzayr, accepting co-authorship
>
> Good afternoon Dr Zhao
>
> I gladly approve being a co-author regarding GemC1-MCIDAS transcriptional network in choroid plexus as a barrier for tumorigenesis
>
> I will likely end up in pathology residency by July but if I can help in any future projects with figures or anything else, please feel free to contact me.

All the best,

Uzayr

Haotian Zhao

From: Ahmed Abdeltawab <aabdel20@nyit.edu>
Sent: Saturday, January 22, 2022 5:08 AM
To: Haotian Zhao
Subject: Re: Manuscript

Hi Dr. Zhao,

Thank you, hope all is well with you too. That is great news to start off the new year. I approve of the change stated in the email.

Best Regards,

Ahmed Abdeltawab

Sent from my iPhone

> On Jan 21, 2022, at 2:52 PM, Haotian Zhao <hzhao10@nyit.edu> wrote:
>
> Hi Ahmed,
>
> I hope everything is well with you. It took a long time in the making, but I am glad to tell you that our manuscript "Disruption of GMNC-MCIDAS multiciliogenesis program is critical in choroid plexus carcinoma development" has been provisionally accepted for publication in Cell Death and Differentiation. I am currently finalizing the manuscript for official acceptance.
>
> During the revision process, Mr. James Virga and Dr. Yingtao Zhao have contributed to the study through acquisition and analysis data. They were added as co-authors in the revised manuscript. Dr. Stracker made major contribution to the revisions and was added as co-corresponding author. As a co-author, can you please approve of these changes in authors. Thanks.
>
> Haotian.
>
> -----Original Message-----
> From: Ahmed Abdeltawab <aabdel20@nyit.edu>
> Sent: Friday, January 21, 2022 2:03 PM
> To: Haotian Zhao <hzhao10@nyit.edu>
> Subject: Ahmed
>
> Hi Dr.Zhao,
>
> Do you need me to email you anything specific?
>
> Sent from my iPhone

Haotian Zhao

From: Jun Wang <jwang@explorabiolabs.com>
Sent: Friday, January 21, 2022 2:24 PM
To: Haotian Zhao
Subject: Re: Manuscript

Hi Haotian,

Yes, I approve these changes.

Thanks,
Jun

--
Jun Wang, PhD
Associate Director of Research
Explora Biolabs
11175 Flintkote Avenue, Suite B
San Diego, CA 92121
Office: (858)768-2100 ext 7010
Mobile: (626)780-6031
jwang@explorabiolabs.com

explora biolabs
YOUR SCIENCE:
POWERED BY EXPLORA
17+ YEARS OF VIVARIUM & CONTRACT RESEARCH MANAGEMENT

From: Haotian Zhao <hzhao10@nyit.edu>
Date: Friday, January 21, 2022 at 11:13 AM
To: Jun Wang <jwang@explorabiolabs.com>
Subject: RE: Manuscript

Hi Jun,

I will update your current contact in the manuscript. Mr. James Virga and Dr. Yingtao Zhao have contributed to the study through acquisition and analysis data. They were added as co-authors in the revised manuscript. Dr. Stracker made major contribution to the revisions and was added as co-corresponding author. Please approve of these changes. Thanks.

Haotian.

From: Jun Wang <jwang@explorabiolabs.com>
Sent: Friday, January 21, 2022 1:28 PM
To: Haotian Zhao <hzhao10@nyit.edu>; Robert Wechsler-Reya <rwreya@sbpdiscovery.org>
Subject: Re: Manuscript

Caution: This message originated from an **External Source**. Use proper judgment when opening attachments, clicking links or responding to this email.

Hi Haotian,

Congratulations on the new paper. My current contact information is in my email signature.

Thanks,
Jun

--
Jun Wang, PhD
Associate Director of Research
Explora Biolabs
11175 Flintkote Avenue, Suite B
San Diego, CA 92121
Office: (858)768-2100 ext 7010
Mobile: (626)780-6031
jwang@explorabiolabs.com

explora biolabs

YOUR SCIENCE:
POWERED BY EXPLORA
17+ YEARS OF VIVARIUM & CONTRACT RESEARCH MANAGEMENT

From: Robert Wechsler-Reya <rwreya@sbpdiscovery.org>
Date: Thursday, January 20, 2022 at 8:50 PM
To: Haotian Zhao <hzhao10@nyit.edu>
Cc: Jun Wang <jwang@explorabiolabs.com>
Subject: Re: Manuscript

Congratulations Haotian! The address you have listed for me is correct. I'm copying Jun so he is aware of this, and can provide you with whatever information you need.

I approve of the addition of James Virga and Yingtao Zhao as co-authors, and of Dr. Stracker as co-corresponding author.

Rob

Sanford Burnham Prebys

Robert Wechsler-Reya, Ph.D.
Director, Tumor Initiation & Maintenance Program
Professor, NCI-Designated Cancer Center

SANFORD BURNHAM PREBYS Medical Discovery Institute
[illegible] Torrey Pines Scenic Drive La Jolla, CA 92037
Phone: [illegible]
Fax: [illegible]
Email: [illegible]

Assistant: [illegible]
Phone: [illegible]
Email: [illegible]

From: Haotian Zhao <hzhao10@nyit.edu>
Date: Thursday, January 20, 2022 at 5:06 PM
To: Robert Wechsler-Reya <rwreya@sbpdiscovery.org>
Subject: Manuscript

You don't often get email from hzhao10@nyit.edu. Learn why this is important

[EXTERNAL EMAIL: This message may not be safe, use caution, and please be suspicious of any attachments contained in this email.]

Dear Rob,

I hope everything is well with you. It took a long time in the making, but I am glad to tell you that our manuscript "Disruption of GMNC-MCIDAS multiciliogenesis program is critical in choroid plexus carcinoma development" has been provisionally accepted for publication in Cell Death and Differentiation. I am currently finalizing the manuscript for acceptance and production. Here is what I have for your and Jun's information:

Tumor Initiation and Maintenance Program, Sanford Burnham Prebys Medical Discovery Institute, 10901 North Torrey

Pines Road, La Jolla, California 92037, USA.

Can you please forward the message to Jun since I don't have his current contact. Please let me know if you would like to include your other information.

In addition, during the revision process, Mr. James Virga and Dr. Yingtao Zhao have contributed to the study through acquisition and analysis data. They were added as co-authors in the revised manuscript. Dr. Stracker made major contribution to the revisions and was added as co-corresponding author. Please approve of these changes so I could update the author list. Thanks.

Haotian.

**From:**	Hao LU
**To:**	Haotian Zhao
**Cc:**	Sudipto ROY
**Subject:**	Re: Manuscript
**Date:**	Tuesday, January 25, 2022 6:32:28 AM

Hi, Haotian,
It is great to hear this good news. Yes, I am pleased to accept the manuscript changes.

Best regards,

Lu Hao

From: Haotian Zhao <hzhao10@nyit.edu>
Sent: Tuesday, 25 January 2022 8:58 am
To: Hao LU <luhao@imcb.a-star.edu.sg>
Cc: Sudipto ROY <sudiptor@imcb.a-star.edu.sg>
Subject: RE: Manuscript

Hi Hao,

Can you approve the changes in authors to include Mr. James Virga and Dr. Ying-Tao Zhao as co-authors in the revised manuscript, and Dr. Stracker as co-corresponding author, so I could update the author list and report to the editor of the journal. Thanks.

Haotian

From: Haotian Zhao
Sent: Sunday, January 23, 2022 12:40 PM
To: Hao LU <luhao@imcb.a-star.edu.sg>
Subject: FW: Manuscript

Hi Hao,

Can you approve the changes in authors to include Mr. James Virga and Dr. Ying-Tao Zhao as co-authors in the revised manuscript, and Dr. Stracker as co-corresponding author, so I could update the author list. Thanks.

Haotian.

From: Sudipto ROY <sudiptor@imcb.a-star.edu.sg>
Sent: Saturday, January 22, 2022 1:05 AM
To: Haotian Zhao <hzhao10@nyit.edu>
Cc: Hao LU <luhao@imcb.a-star.edu.sg>
Subject: RE: Manuscript

Caution: This message originated from an **External Source**. Use proper judgment when opening attachments,

clicking links or responding to this email.

Congratulations Haotian!! Yes, I approve these changes to the manuscript.

Best wishes,
Sudipto.

From: Haotian Zhao <hzhao10@nyit.edu>
Sent: Friday, 21 January 2022 9:34 am
To: Sudipto ROY <sudiptor@imcb.a-star.edu.sg>
Cc: Hao LU <luhao@imcb.a-star.edu.sg>
Subject: Manuscript

Dear Sudipto and Hao,

I hope everything is well with you. It took a long time in the making, but I am glad to tell you that our manuscript "Disruption of GMNC-MCIDAS multiciliogenesis program is critical in choroid plexus carcinoma development" has been provisionally accepted for publication in Cell Death and Differentiation. I am currently finalizing the manuscript for acceptance and production. Here is what I have for your and Hao's information:

Institute of Molecular and Cell Biology, Proteos, 61 Biopolis Drive, Singapore 138673.

Department of Pediatrics, Yong Loo Lin School of Medicine, National University of Singapore, 1E Kent Ridge Road, Singapore 119288.

Department of Biological Sciences, National University of Singapore, 14 Science Drive 4, Singapore 117543.

S.R. is supported by funds from the A*STAR, Singapore.

Please let me know if you would like to include your other information. In addition, during the revision process, Mr. James Virga and Dr. Yingtao Zhao have contributed to the study through acquisition and analysis of data. They were added as co-authors in the revised manuscript. Dr. Stracker made major contribution to the revisions and was added as co-corresponding author. Please approve of these changes so I could update the author list. Thanks.

Haotian.

This e-mail and any attachments are only for the use of the intended recipient and may contain material that is confidential, privileged and/or protected by the Official Secrets Act. If you are not the intended recipient, please delete it or notify the sender immediately. Please do not copy or use it for any purpose or disclose the contents to any other person.
This e-mail and any attachments are only for the use of the intended recipient and may contain

material that is confidential, privileged and/or protected by the Official Secrets Act. If you are not the intended recipient, please delete it or notify the sender immediately. Please do not copy or use it for any purpose or disclose the contents to any other person.

Haotian Zhao

From: Lidov, Hart <Hart.Lidov@childrens.harvard.edu>
Sent: Friday, January 21, 2022 10:15 AM
To: Haotian Zhao
Subject: RE: Manuscript [EXTERNAL]

Yes I approve --- tx Hart

From: Haotian Zhao <hzhao10@nyit.edu>
Sent: Friday, January 21, 2022 9:37 AM
To: Lidov, Hart <Hart.Lidov@childrens.harvard.edu>
Subject: RE: Manuscript [EXTERNAL]

*** External Email - Caution ***

Thank you Hart. Would you also please approve of the changes in the authors to reflect their contributions during the revision process. Thanks.

From: Lidov, Hart <Hart.Lidov@childrens.harvard.edu>
Sent: Friday, January 21, 2022 8:24 AM
To: Haotian Zhao <hzhao10@nyit.edu>
Cc: Fame, Ryan <Ryan.Fame@childrens.harvard.edu>; Lehtinen, Maria <Maria.Lehtinen@childrens.harvard.edu>
Subject: RE: Manuscript [EXTERNAL]

Caution: This message originated from an **External Source**. Use proper judgment when opening attachments, clicking links or responding to this email.

Congratulations.. good work..

(credit line looks right to me)

tx Hart

From: Haotian Zhao <hzhao10@nyit.edu>
Sent: Thursday, January 20, 2022 8:01 PM
To: Lehtinen, Maria <Maria.Lehtinen@childrens.harvard.edu>
Cc: Fame, Ryan <Ryan.Fame@childrens.harvard.edu>; Lidov, Hart <Hart.Lidov@childrens.harvard.edu>
Subject: Manuscript [EXTERNAL]

*** External Email - Caution ***

Dear Maria, Ryann and Hart,

I hope everything is well with you. It took a long time in the making, but I am very glad to tell you that our manuscript "Disruption of GMNC-MCIDAS multiciliogenesis program is critical in choroid plexus carcinoma development" has been provisionally accepted for publication in Cell Death and Differentiation. I am currently finalizing the manuscript for acceptance and production. Here is what I have for your information:

Department of Pathology, Boston Children's Hospital, Boston, Massachusetts 02115, USA.

This project was supported by NIH T32 HL110852 and BCH Faculty Development Fellowship (R.M.F.); NIH R01 NS088566 (MKL) and the New York Stem Cell Foundation (M.K.L.). M.K. Lehtinen is a New York Stem Cell Foundation – Robertson Investigator.

Please let me know if you would like to include your other information. In addition, during the revision process, Mr. James Virga and Dr. Yingtao Zhao have contributed to the study through acquisition and analysis data. They were added as co-authors in the revised manuscript. Dr. Stracker made major contribution to the revisions and was added as co-corresponding author. Please approve of these changes so I could update the author list. Thanks.

Haotian.

Haotian Zhao

From: Surendran,Kameswaran <Kameswaran.Surendran@SanfordHealth.org>
Sent: Tuesday, January 25, 2022 10:35 AM
To: Haotian Zhao
Subject: RE: Manuscript

Hi Haotian,

I am fine with the authors changes, you know best.

Thanks,

Kamesh

From: Haotian Zhao <hzhao10@nyit.edu>
Sent: Tuesday, January 25, 2022 9:27 AM
To: Surendran,Kameswaran <Kameswaran.Surendran@SanfordHealth.org>
Subject: [EXTERNAL] Manuscript

Hi Kamesh,

Can you approve of the author change so I could update with the journal editorial office. Thanks.

Haotian.

From: Haotian Zhao
Sent: Friday, January 21, 2022 9:34 AM
To: Surendran,Kameswaran <Kameswaran.Surendran@SanfordHealth.org>
Subject: RE: Manuscript

Hi Kamesh,

Sorry I forgot to send the manuscript files. Here you go. I will update your information in the final version. In addition, during the revision process, Mr. James Virga and Dr. Yingtao Zhao have contributed to the study through acquisition and analysis data. They were added as co-authors in the revised manuscript. Dr. Stracker made major contribution to the revisions and was added as co-corresponding author. Please approve of these changes. Thanks.

Haotian.

From: Surendran,Kameswaran <Kameswaran.Surendran@SanfordHealth.org>
Sent: Friday, January 21, 2022 9:17 AM
To: Haotian Zhao <hzhao10@nyit.edu>
Subject: RE: Manuscript

Caution: This message originated from an **External Source**. Use proper judgment when opening attachments, clicking links or responding to this email.

Hi Haotian,

It is good to hear from you. Hope all is well for you and your family.
We are doing okay.

Thanks for including me on your paper. Please do send me the recent version of the provisionally accepted paper.
Please use the following if possible as my information:

Pediatrics and Rare Diseases Group, Sanford Research, 2301 E 60th Street North, Sioux Falls, South Dakota 57104, USA.

Please let me know if I can help with anything research related.

Best wishes,

Kamesh

Kamesh Surendran Ph.D.
Scientist, Pediatrics and Rare Diseases Group,
Sanford Research
2301 E. 60th Street North
Sioux Falls, SD 57104

Telephone: 605-312-6415
Fax: 605-312-6071

Website: Surendran Lab
Vdqirug#Uhvhdufk#Z hevlwh#kwwsv=22uhvhdufk1vdqirugkhdowk1ruj2#
Vdqirug#Khdowk#Z hevlwh#kwwsv=22z z z 1vdqirugkhdowk1ruj2#

SANFORD RESEARCH

From: Haotian Zhao <hzhao10@nyit.edu>
Sent: Thursday, January 20, 2022 6:49 PM
To: Surendran,Kameswaran <Kameswaran.Surendran@SanfordHealth.org>
Subject: [EXTERNAL] Manuscript

Hi Kamesh,

I hope everything is well with you and family. I am very glad to tell you that our manuscript "Disruption of GMNC-MCIDAS multiciliogenesis program is critical in choroid plexus carcinoma development" has been provisionally accepted for publication in Cell Death and Differentiation. I am currently finalizing the manuscript for acceptance and production. Here is what I have for your information:

Children's Health Research Center, Sanford Research, 2301 E 60th Street North, Sioux Falls, South Dakota 57104, USA.

Please let me know if you would like to include your other information. In addition, during the revision process, Mr. James Virga and Dr. Yingtao Zhao have contributed to the study through acquisition and analysis data. They were added

as co-authors in the revised manuscript. Dr. Stracker made major contribution to the revisions and was added as co-corresponding author. Please approve of these changes so I could update the author list. Thanks.

Haotian.

Confidentiality Notice: This e-mail message, including any attachments,
is for the sole use of the intended recipient(s) and may contain
privileged and confidential information. Any unauthorized review, use,
disclosure or distribution is prohibited. If you are not the intended
recipient, please contact the sender by reply e-mail and destroy
all copies of the original message.

Confidentiality Notice: This e-mail message, including any attachments,
is for the sole use of the intended recipient(s) and may contain
privileged and confidential information. Any unauthorized review, use,
disclosure or distribution is prohibited. If you are not the intended
recipient, please contact the sender by reply e-mail and destroy
all copies of the original message.

From: 吴力钊
To: Haotian Zhao
Subject: Re:Manuscript
Date: Monday, January 24, 2022 7:47:58 AM

Caution: This message originated from an **External Source**. Use proper judgment when opening attachments, clicking links or responding to this email.

Dear Haotian,

Congratulations on the job well done!

I fully support and approve your proposed addition of Mr. Virga and Dr. Zhao as co-authors, and Dr. Stracker as a co-corresponding author.

Best,

Lizhao

------------------ Original ------------------

From: "Haotian Zhao"<hzhao10@nyit.edu>;
Date: Mon, Jan 24, 2022 07:45 AM
To: "吴力钊"<lzwu@cmu.edu.cn>;
Subject: Manuscript

Dear Lizhao,

I hope everything is well with you. It took a long time in the making, but I am glad to tell you that our manuscript "Disruption of GMNC-MCIDAS multiciliogenesis program is critical in choroid plexus carcinoma development" has been provisionally accepted for publication in Cell Death and Differentiation. I am currently finalizing the manuscript for acceptance and production. Here is what I have for your and Jun's information:

Department of Pathophysiology, College of Basic Medical Sciences, China Medical University,

Shenyang 110122, People's Republic of China.

Please let me know if you would like to include your other information. In addition, during the revision process, Mr. James Virga and Dr. Yingtao Zhao have contributed to the study through acquisition and analysis data. They were added as co-authors in the revised manuscript. Dr. Stracker made major contribution to the revisions and was added as co-corresponding author. Please approve of these changes so I could update the author list. Thanks.

Haotian.

Haotian Zhao

From: Ulrich Schüller <u.schueller@uke.de>
Sent: Friday, January 21, 2022 6:28 PM
To: Haotian Zhao
Subject: Re: Manuscript

Dear Haotian,
Approved!
Ulrich

Am 22.01.2022 um 00:07 schrieb Haotian Zhao <hzhao10@nyit.edu>:

Dear Ulrich,

Mr. James Virga and Dr. Yingtao Zhao have contributed to the study through acquisition and analysis data. They were added as co-authors in the revised manuscript. Dr. Stracker made major contribution to the revisions and was added as co-corresponding author. Can you please approve of these changes in authors. I will relay the information to the journal. Thanks.

Haotian.

From: Prof. Dr. Ulrich Schüller <u.schueller@uke.de>
Sent: Friday, January 21, 2022 2:56 AM
To: Haotian Zhao <hzhao10@nyit.edu>
Subject: RE: Manuscript

Caution: This message originated from an **External Source**. Use proper judgment when opening attachments, clicking links or responding to this email.

Dear Haotian,
what a coincidence! I just wrote you yesterday, but realized today that this was obviously the wrong address. Please see attached my message.
Congratulations to the paper, that fantastic news! My affiliations are correct, and I will be excited to see the paper in print.
Looking forward to hearing back from you,
Ulrich

Ulrich Schüller, MD
Professor for Molecular Pediatric Neurooncology
Consultant Neuropathologist

University Medical Center Hamburg-Eppendorf
Research Institute Children's Cancer Center
Martinistrasse 52, N63 (HPI), D-20251 Hamburg, Germany
Phone: ++49-40-426051240; Fax: ++49-40-741040350
E-mail: u.schueller@uke.de

http://www.kinderkrebs-forschung.de/en/research/schueller-research-group/

From: Haotian Zhao [mailto:hzhao10@nyit.edu]
Sent: Freitag, 21. Januar 2022 02:01
To: Prof. Dr. Ulrich Schüller
Subject: Manuscript

Dear Ulrich,

I hope everything is well with you. I am very glad to tell you that our manuscript "Disruption of GMNC-MCIDAS multiciliogenesis program is critical in choroid plexus carcinoma development" has been provisionally accepted for publication in Cell Death and Differentiation. I am currently finalizing the manuscript for acceptance and production. Here is what I have for your information:

Research Institute Children's Cancer Center, University Medical Center Hamburg-Eppendorf, 20246

Hamburg, Germany.

Institute of Neuropathology, University Medical Center Hamburg-Eppendorf, 20246 Hamburg,

Germany.

Department of Pediatric Hematology and Oncology, University Medical Center Hamburg-Eppendorf,

20246 Hamburg, Germany.

U.S. is supported by the Fördergemeinschaft Kinderkrebszentrum Hamburg.

Please let me know if you would like to include your other information. In addition, during the revision process, Mr. James Virga and Dr. Yingtao Zhao have contributed to the study through acquisition and analysis data. They were added as co-authors in the revised manuscript. Dr. Stracker made major contribution to the revisions and was added as co-corresponding author. Please approve of these changes so I could update the author list. Thanks.

Haotian.

Universitätsklinikum Hamburg-Eppendorf; Körperschaft des öffentlichen Rechts; Gerichtsstand: Hamburg
| www.uke.de
Vorstandsmitglieder: Prof. Dr. Burkhard Göke (Vorsitzender), Joachim Prölß, Prof. Dr. Blanche Schwappach-Pignataro, Marya Verdel

SAVE PAPER - THINK BEFORE PRINTING

Universitätsklinikum Hamburg-Eppendorf; Körperschaft des öffentlichen Rechts; Gerichtsstand: Hamburg | www.uke.de
Vorstandsmitglieder: Prof. Dr. Burkhard Göke (Vorsitzender), Joachim Prölß, Prof. Dr. Blanche Schwappach-Pignataro, Marya Verdel

SAVE PAPER - THINK BEFORE PRINTING

Haotian Zhao

From: Lehtinen, Maria <Maria.Lehtinen@childrens.harvard.edu>
Sent: Thursday, January 20, 2022 9:28 PM
To: Fame, Ryan; Haotian Zhao
Cc: Lidov, Hart
Subject: Re: Manuscript [EXTERNAL]

Caution: This message originated from an **External Source**. Use proper judgment when opening attachments, clicking links or responding to this email.

Dear Haotian,

Wonderful news to start off the new year - congratulations!

I approve the author updates - we are happy to have been able to contribute and participate in the study. Please share the final accepted version and proofs when you get them.

The funding support looks good to me. Could please adjust my initials after the NIH grant to have periods between the letters, just as you have for the other grants.

Best,

-Maria.

From: Fame, Ryan
Sent: Thursday, January 20, 2022 8:08 PM
To: Haotian Zhao
Cc: Lidov, Hart; Lehtinen, Maria
Subject: Re: Manuscript [EXTERNAL]

Dear Haiotian~

Congratulations on the acceptance to publish this important work! It has been a pleasure working with your team and I thank you for including me in this investigation. I would love to read the accepted version when it is convenient to send.

The affiliation and support look correct to me and I approve the author changes you have included. You likely already have it, but if you need my ORCID it is: 0000-0002-8244-2624

Sincerely
Ryann

From: Haotian Zhao <hzhao10@nyit.edu>
Sent: Thursday, January 20, 2022 8:00 PM
To: Lehtinen, Maria
Cc: Fame, Ryan; Lidov, Hart
Subject: Manuscript [EXTERNAL]

*** External Email - Caution ***

Dear Maria, Ryann and Hart,

I hope everything is well with you. It took a long time in the making, but I am very glad to tell you that our manuscript "Disruption of GMNC-MCIDAS multiciliogenesis program is critical in choroid plexus carcinoma development" has been provisionally accepted for publication in Cell Death and Differentiation. I am currently finalizing the manuscript for acceptance and production. Here is what I have for your information:

Department of Pathology, Boston Children's Hospital, Boston, Massachusetts 02115, USA.

This project was supported by NIH T32 HL110852 and BCH Faculty Development Fellowship (R.M.F.); NIH R01 NS088566 (MKL) and the New York Stem Cell Foundation (M.K.L.). M.K. Lehtinen is a New York Stem Cell Foundation – Robertson Investigator.

Please let me know if you would like to include your other information. In addition, during the revision process, Mr. James Virga and Dr. Yingtao Zhao have contributed to the study through acquisition and analysis data. They were added as co-authors in the revised manuscript. Dr. Stracker made major contribution to the revisions and was added as co-corresponding author. Please approve of these changes so I could update the author list. Thanks.

Haotian.

**From:**	Stracker, Travis (NIH/NCI) [E]
**To:**	Haotian Zhao
**Subject:**	Re: [EXTERNAL] RE: CAN-20-3750 sent out for external peer review by Cancer Research
**Date:**	Thursday, January 20, 2022 9:21:16 PM

Hi Haotian,

Congrats on the paper! That is great news!

My ORCID is: **0000-0002-8650-2081**
Berta's ORCID is: **0000-0002-0382-6129**

The other info you have for Berta and I is correct, although she is leaving the Crick and not sure if the email is still in effect, I will text her tomorrow if there is no response. Please make some minor corrections if possible:

Affiliations:
Institute for Research in Biomedicine (IRB Barcelona), The Barcelona Institute of Science and Technology, C/ Baldiri Reixac 10, Barcelona 08028, Spain. *this is the same as what you had

Radiation Oncology Branch, Center for Cancer Research, National Cancer Institute, NIH, 9000 Rockville Pike, Building 10, Bethesda, MD 20892, USA
***This is the full address if needed**

Present address: The Francis Crick Institute, 1 Midland Road, London NW1 1AT, UK *this is the same as what you had

Funding:
T.H.S. was funded by the Spanish Ministry of Science, Innovation and Universities (MCIU: PGC2018-095616-B-I00/GINDATA and FEDER) and **the Intramural Research Program of the National Institutes of Health, National Cancer Institute.**

I am fine with any of the author changes you feel are appropriate.

Best regards,

Travis

From: Haotian Zhao <hzhao10@nyit.edu>
Date: Thursday, January 20, 2022 at 9:06 PM
To: "Stracker, Travis (NIH/NCI) [E]" <travis.stracker@nih.gov>
Subject: [EXTERNAL] RE: CAN-20-3750 sent out for external peer review by Cancer Research

Haotian Zhao

From: Haotian Zhao
Sent: Thursday, January 20, 2022 8:33 PM
To: Stracker, Travis (NIH/NCI) [E]
Cc: berta.terre@crick.ac.uk
Subject: Manuscript

Dear Travis and Berta,

I hope everything is well with you. It took a long time in the making, but I am glad to tell you that our manuscript "Disruption of GMNC-MCIDAS multiciliogenesis program is critical in choroid plexus carcinoma development" has been provisionally accepted for publication in Cell Death and Differentiation. I am currently finalizing the manuscript for acceptance and production. Here is what I have for your and Berta's information:

Institute for Research in Biomedicine (IRB Barcelona), The Barcelona Institute of Science and Technology, C/ Baldiri

Reixac 10, Barcelona 08028, Spain.

The National Cancer Institute, Center for Cancer Research, Radiation Oncology Branch, Bethesda MD, 20892, USA.

Present address: The Francis Crick Institute, 1 Midland Road, London NW1 1AT, UK

T.H.S. was supported by the Spanish Ministry of Science, Innovation and Universities (MCIU: PGC2018-095616-B-

I00/GINDATA and FEDER), the Centres of Excellence Severo Ochoa award and the CERCA Programme and is currently

supported by the NCI Intramural program.

Please let me know if you would like to include your other information. In addition, during the revision process, Mr. James Virga and Dr. Yingtao Zhao have contributed to the study through acquisition and analysis data. They were added as co-authors in the revised manuscript. Dr. Stracker made major contribution to the revisions and was added as co-corresponding author. Please approve of these changes so I could update the author list. Thanks.

Haotian.
